# Synergistic Catalysis in Heterobimetallic Complexes for Homogeneous Carbon Dioxide Hydrogenation

**DOI:** 10.3390/molecules28062574

**Published:** 2023-03-12

**Authors:** Zeno B. G. Fickenscher, Peter Lönnecke, Anna K. Müller, Oldamur Hollóczki, Barbara Kirchner, Evamarie Hey-Hawkins

**Affiliations:** 1 Institute of Inorganic Chemistry, Universität Leipzig, Johannisallee 29, 04103 Leipzig, Germany; 2 Mulliken Center for Theoretical Chemistry, Institute for Physical and Theoretical Chemistry, Beringstr. 4, 53115 Bonn, Germany; 3 Department of Physical Chemistry, Faculty of Science and Technology, University of Debrecen, Egyetem ter 1, H-4010 Debrecen, Hungary

**Keywords:** bimetallic complex, carbon dioxide, hydrogenation, synergistic catalysis

## Abstract

Two heterobimetallic Mo,M’ complexes (M’ = Ir^III^, Rh^III^) were synthesized and fully characterized. Their catalytic activity in homogeneous carbon dioxide hydrogenation to formate was studied. A pronounced synergistic effect between the two metals was found, most notably between Mo and Ir, leading to a fourfold increase in activity compared with a binary mixture of the two monometallic counterparts. This synergism can be attributed to spatial proximity of the two metals rather than electronic interactions. To further understand the nature of this interaction, the mechanism of the CO_2_ hydrogenation to formate by a monometallic Ir^III^ catalyst was studied using computational and spectroscopic methods. The resting state of the reaction was found to be the metal-base adduct, whereas the rate-determining step is the inner-sphere hydride transfer to CO_2_. Based on these findings, the synergism in the heterobimetallic complex is beneficial in this key step, most likely by further activating the CO_2_.

## 1. Introduction

**Figure 1:**
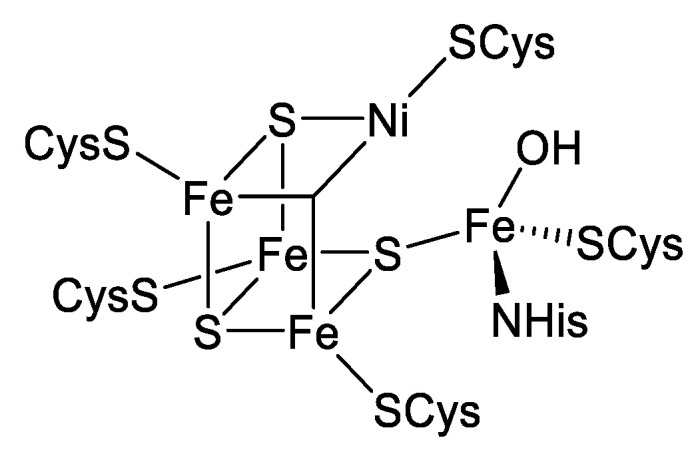
Active site of Ni-CODH [6].

As society is faced with ever-increasing atmospheric CO_2_ levels and its impact on the world climate, chemists strive to develop novel catalysts that can create value-added products from abundant and environmentally benign substrates and reagents [1,2]. A common approach is taking inspiration from nature—more specifically, enzymes which have fast conversion rates, high selectivity, and low activation energies [3,4]. Several me-talloproteins, capable of redox transformations of small molecules such as H_2_, CO_2_, N_2_ and CO, employ multi- and bimetallic assemblies [5]. One example is the metalloenzyme carbon monoxide dehydrogenase (Ni-CODH, Figure 1), in which the cooperation of the Fe and the Ni center enable a fast and selective reduction of CO_2_ to CO [6].

Inspired by this approach, a plethora of heterobimetallic complexes have been developed, which show superior catalytic behavior compared with their monometallic counterparts. In most of these cases, the increase in activity can be attributed to the beneficial electronic interaction between the two metals, which means that exclusively one metal center performs all substrate transformations [7,8,9,10,11,12,13]. A different approach is to utilize the reactivity of each metal to facilitate tandem or one-pot reactions, meaning both metals interact directly with substrates [14,15,16,17,18,19,20]. However, in biological systems such as Ni-CODH, both metals are used to perform a single reaction [6]. Synergistic effects within a heterobimetallic complex have only recently been recognized and utilized. For example, heterobimetallic Mg^II^–Zn^II^ complexes are very active catalysts for ring-opening copolymerization reactions between CO_2_ and epoxides [21,22,23]. Synergistic effects are also vital in the activity of heterobimetallic catalysts for olefin hydrogenation and formate dehydrogenation [24].

Herein, we report the utilization of heterobimetallic Mo^0^,M’ complexes (M’ = Rh^III^ (**C2**), Ir^III^ (**C1**)) for homogeneous CO_2_ hydrogenation to formate salts. Homogeneous hydrogenation of CO_2_ to formic acid and formate salts is a fast-developing field, ever since the pioneering works of Inoue [25]. While formic acid is widely used as silage acid, it is also interesting for potential application in chemical H_2_ storage [25,26]. Currently, there are multiple examples of precious metal catalysts with very high turnover numbers (TON) and turnover frequency (TOF), as well as several base metal catalysts with similarly high TON and TOF [27,28,29,30]. Recently, heterobimetallic Ni^0^–Ga^III^ and Co^-I^–Ga^III^ complexes with outstanding performance have been reported. However, in both examples, the second metal (Ga) is used to impart nobility on the first (Co/Ni) through a metal–metal bond [31,32,33].

## 2. Results and Discussion

### 2.1. Complex Synthesis

**Scheme 1:**
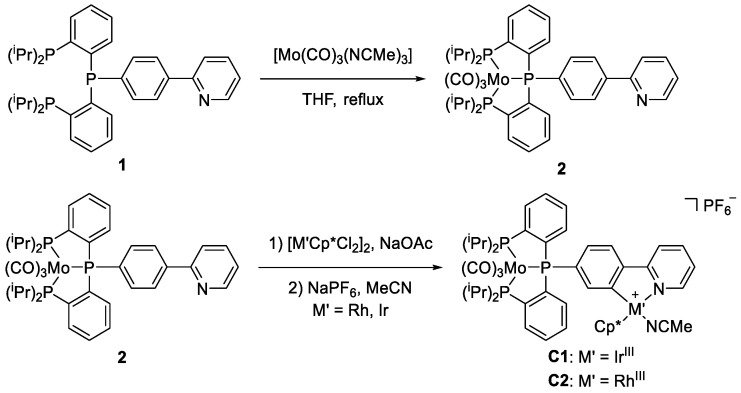
Synthesis of heterobimetallic complexes **C1** and **C2** (Cp* = C_5_Me_5_).

In the synthesis of heterobimetallic complexes, two main challenges arise: selectivity and stability. For a complex with two different catalytic sites, the metals must be selectively bound and kinetically stable within each respective coordination sphere. In the ditopic ligand **1** (Scheme 1), two distinctively different coordination environments are available. Through a stepwise coordination, complexes **C1** and **C2** can be obtained in good to excellent yields. [Mo^0^(CO)_3_(NCMe)_3_] was chosen as a precursor as the resulting complex **2** (Scheme 1) is stable enough to undergo subsequent cyclometallation with [M^III^Cp*Cl_2_]_2_ (M = Ir or Rh, Cp* = C_5_Me_5_). The resulting complexes **C1** and **C2** were fully characterized, including single crystal structure determinations. Additionally, the kinetic stability was tested using solutions of each complex in acetonitrile-d_3_, which showed no change over the course of a week in the ^1^H- and ^31^P{^1^H}-NMR spectra. Furthermore, a 1:1 mixture of the monometallic complexes **M1** and **M3** (see Table 1) was studied, showing no reaction. This indicates a high kinetic stability of the metals in their respective coordinative environments.

### 2.2. Catalysis

All complexes were tested for catalytic activity towards CO_2_ hydrogenation (Table 1). It is essential to activate the complexes, by first applying H_2_ for 30 min and then adding CO_2_. If both CO_2_ and H_2_ are added simultaneously, no catalytic activity can be observed. Different conditions (solvents, base, H_2_/CO_2_ pressure, and reaction time) were investigated (for a comprehensive list of the tested conditions see electronic supporting information). Since **C1** outperformed **C2** significantly, the focus of this study is on the Mo,Ir complex **C1**. The system performed best with acetonitrile as solvent and a strong base, such as 1,8-diazabicyclo[5.4.0]undec-7-ene (DBU) or 1,1,3,3-tetramethylguanidine (TMG) (Table 1, entries 1, 8). Using a weaker base such as triethylamine led to a significant decrease in TON (Table 1, entry 9). Strong inorganic bases such as LiOH or Cs_2_CO_3_ led to no observable formate production, probably due to their insolubility in acetonitrile. The necessity of using strong organic bases can be understood by comparing the pK_aH_ of the organic base and formic acid in acetonitrile [34,35]. While DBU (pK_aH_ = 24.31) and TMG (pK_aH_ = 23.35) are sufficiently strong bases to quantitively deprotonate formic acid (pK_aH_ = 20.7), triethylamine (pK_aH_ = 18.83) and diisopropylethylamine (DIPEA, pK_aH_ = 18.2) are not [34,35]. Since the hydrogenation of CO_2_ to formic acid is an inherently endergonic reaction, the base must be strong enough to provide a thermodynamic driving force. In the absence of base, no reaction occurs (Table 1, entry 10). 

After 70 h, with TMG as base, more than the expected stoichiometric amounts of formic acid were formed, namely two equivalents in relation to the amount of base employed. Increasing the reaction time to 140 h did not result in even higher yields (see electronic supporting information, Table S4), indicating that the reaction stops when two equivalents of formic acid have been formed. While unexpected, higher than the expected stoichiometric amounts of formic acid in relation to the base have been reported before and are due to the formation of a formate-formic acid dimer [36]. Counterion effects in **M1** and complexes similar to **M1** have been studied; while higher catalytic activity could most likely be achieved employing an even more weakly coordinating anion, studying these effects in depth is beyond the scope of the present article [37,38].

Using more polar solvents such as DMSO, water or methanol led to a significantly lower TON. Reducing the total pressure from 60 bar to 30 bar led to a tenfold drop in reactivity (entry 11). The TOF for complex **C1**, using TMG as base (see electronic supporting information, Table S4), was determined by monitoring the reaction and is 5.0 h^−1^.

The performance of the monometallic species **M1** or **M3** is poorer than the 1:1 mixture of **M1** and **M3**, especially considering that **M3** is catalytically inactive. This shows that there is a synergistic effect between the two complexes. This synergistic effect is much more pronounced in the heterobimetallic complex **C1**, leading to a reactivity that is ~4 times as high as the mixture of **M1** and **M3** (entry 1 vs. entry 4, Table 1). Furthermore, there is no observable synergistic effect between **M2** and **M3** (entry 6 vs. entry 7, Table 1) but in the heterobimetallic complex **C2**, leading to a threefold increase in reactivity compared to **M2** (Table 1, entry 5 vs. entry 6).

### 2.3. Comparing Heterobimetallic and Monometallic Systems

The spectroscopic data of the heterobimetallic complexes **C1** and **C2** were compared to **M3** to elucidate whether the increased reactivity in the heterobimetallic complexes stems from an actual synergistic effect or is due to a beneficial electronic interaction. The chemical shift of the central phosphorus atom in the ligand of all three complexes is almost identical (79.7 ppm in **M3** vs. 80.2 and 80.5 ppm in **C1** and **C2**, respectively; see supporting information). The CO stretching vibrations of **M3**, **C2** and **C1** are almost identical (two signals at 1930 cm^−1^ and 1845 cm^−1^).

**Figure 2:**
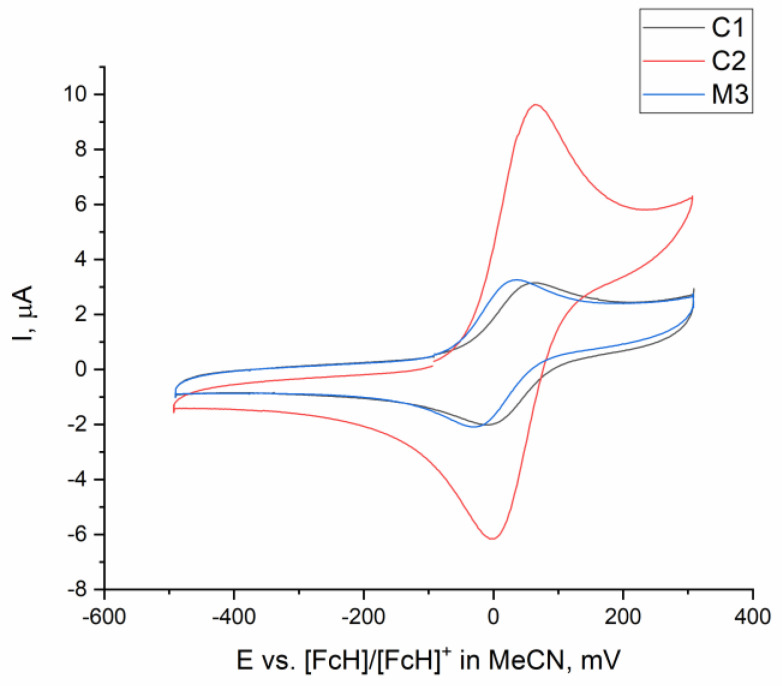
Cyclic voltammogram of **M3** (1 mM), **C1** (1 mM) and, **C2** (3 mM) in MeCN at room temperature, with 0.10 M [N(^n^Bu)_4_]PF_6_ as supporting electrolyte, a glassy carbon electrode (surface area 0.001 cm^2^), a Ag/AgNO_3_ (0.01 mol∙l^−1^) reference electrode, a Pt wire (99.9%) counter electrode, and a voltage sweep rate ν = 100 mV∙s^−1^.

Most notably, however, the experimental *E*_1/2_ values for Mo^0/I^ are only about 30 mV apart (Figure 2; *E*_1/2_(**M3**) = 5 mV, *E*_1/2_(**C1**) = 25 mV, *E*_1/2_(**C2**) = 35 mV, vs. [FcH]/[FcH]^+^). When measuring the *E*_1/2_ values of Mo^0/I^ for **M3** while **M1** or **M2** is present in the same concentration, one can see a very small shift (*E*_1/2_(**M3** and **M2**) = 12 mV, *E*_1/2_(**M3** and **M1**) = 15 mV, see Sections 8.2 and 8.3 in the supporting information) The very similar electrochemical potential as well as the spectroscopic data suggest that the Mo centers in **C1**, **C2** and **M3** are electronically very similar, which in turn means that the Ir center of **C1** and **M1** and the Rh center of **C2** and **M2** must also be comparable. It further indicates that any increase in reactivity must be from synergistic effects between the two metal centers and cannot be attributed to electronic interactions. This is in contrast to other synergistic heterobimetallic systems, where an electronic interaction between the metals is very likely and the increase in reactivity is probably caused by a combination of synergism and electronic interaction [21,22,23,24,39].

**Figure 3:**
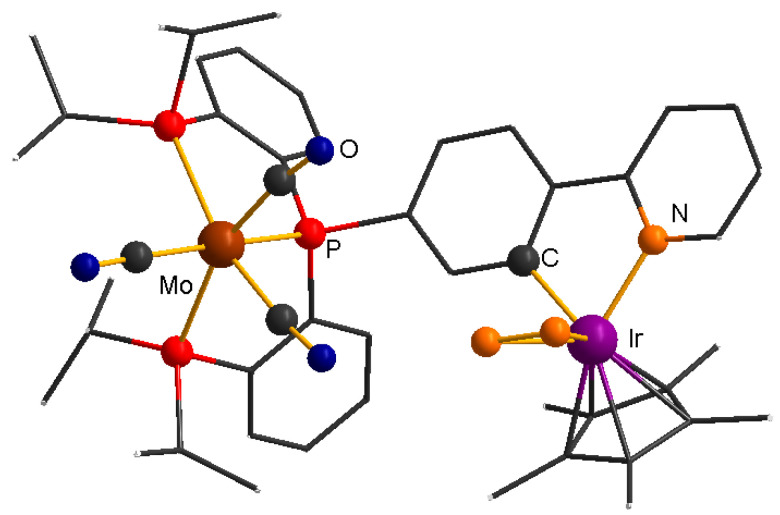
Molecular structure of a derivative of complex **C1** in which the coordinated MeCN has been replaced by N_2_ during crystallization. The non-coordinating PF_6_^−^ counterion, all non-coordinating solvent molecules and hydrogen atoms have been omitted for clarity. All non-coordinating carbon atoms and Cp* are drawn as wireframe.

Similar to natural enzymes, the synergism in **C1** and **C2** is likely caused by the close spatial proximity of the two metals. While **C1** does not crystallize readily, single crystals were obtained of a complex in which the labile MeCN ligand was exchanged with N_2_ (Figure 3). The intermetallic distance Mo···Ir is 6.731(1) Å is longer than the proposed ideal intermetallic distance of 3.5–6 Å, but the metals are not ideally aligned to achieve the shortest possible distance [40]. Additionally, the closest approach of a CO ligand at the Mo center to the N_2_ at the Ir atom is 3.7589(3) Å. This suggests that substrates bound at one metal can readily interact with a ligand and/or substrates bound at the second metal.

### 2.4. Understanding the CO_2_ Hydrogenation Using **M1** as Catalyst

Considering that the two metals in **C1** and **C2** are not electronically communicating, the monometallic complexes **M1** and **M2** can be used as models for the Ir and Rh center in the heterobimetallic complexes, reducing the complexity of the system considerably. Furthermore, studying the heterobimetallic system directly has several disadvantages. The main challenge is that any changes at either metal lead to small shifts in the ^1^H- and ^31^P-NMR spectra, which renders interpreting the spectra difficult, or even impossible. Since NMR spectroscopy is the only analytical technique available to us, which can be performed under an H_2_ or CO_2_ atmosphere, without conclusive NMR measurement no mechanistic insight could be gained into the heterobimetallic system.

During catalytic trials **M3** showed no catalytic activity as the complex is unable to form the necessary hydride species to perform the reaction; thus, we focused the subsequent mechanistic investigations on **M1**, as the Mo center in **C1** only has an auxiliary role in the mechanism. Furthermore, a thorough understanding how **M1** performs CO_2_ hydrogenation reactions can provide a basis for including synergistic interactions later. As the two metals are not in electronic communication, one can deduce that the two metals will be electronically isolated in the catalytically active species as well. Therefore, the catalytic cycle of **M1** is expected to bear strong resemblance to that of **C1**, the main difference being that the Mo center has a beneficial interaction during the rate-determining step (RDS). While additional interactions are possible, they will only have an influence if the activation energy of the RDS is lowered so significantly that a new RDS arises. Nevertheless, this means that by identifying the RDS in the CO_2_ hydrogenation, using **M1** as catalyst the nature of the synergistic interaction becomes more apparent, as an energetically lower transition state of the RDS is essential for higher TON and TOF.

**Figure 4:**
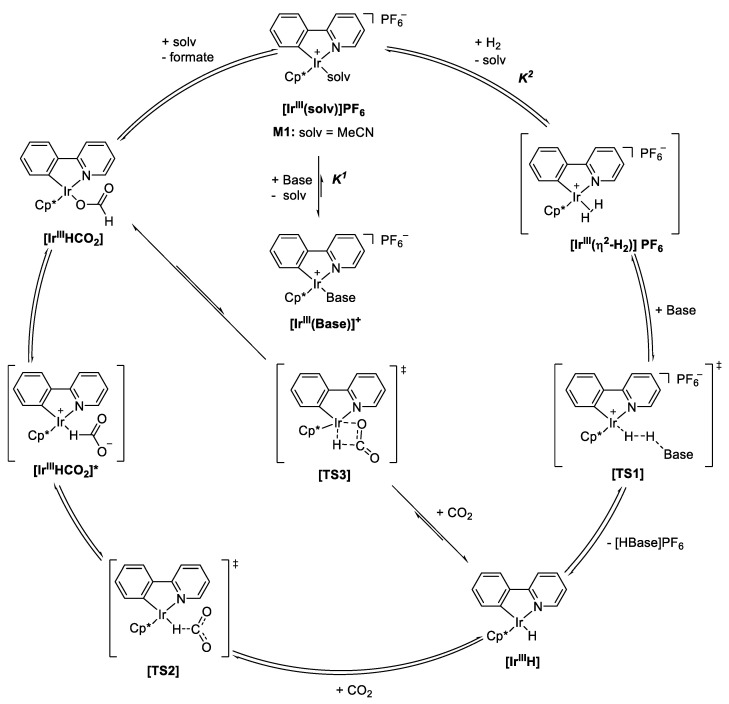
Proposed catalytic scheme for CO_2_ hydrogenation using a monometallic iridium catalyst (Cp* = C_5_Me_5_).

Even before the catalytic cycle (Figure 4) begins, there are two parameters to be considered. The first is the coordinative strength of the solvent, quantified by the free binding enthalpy (ΔG^0^_bind_). These values were calculated for acetonitrile (−50.2 kJ mol^−1^) and for DMSO (−158.9 kJ mol^−1^, coordination through the oxygen atom). The strong coordination of DMSO explains the low activity when the latter is used as a solvent. The resulting **[Ir^III^(DMSO)]^+^** complex (Figure 4) would act as a thermodynamic sink. While DMSO binds too strongly, THF, methanol and water show only weak coordination (ΔG^0^_bind_ = −36.9 kJ mol^−1^, −29.9 kJ mol^−1^, and −32.1 kJ mol^−1^, respectively). The lack of stabilization by the solvent leads to instability of **[Ir^III^(solv)]^+^**. Accordingly, when **M1** is subjected to 1 atm of H_2_ atmosphere in THF-d_8_, signals similar to reported Rh nanoclusters can be observed in the ^1^H-NMR spectra [41]. Thus, MeCN is an ideal solvent as it provides good stabilization of the Ir complex, while not binding too strongly.

Both DBU and TMG are sufficiently strong bases to provide a driving force for the reaction by deprotonating the product formic acid; they also bind strongly to the metal center [42]. For the coordination of DBU to **M1**, the calculated free binding enthalpy in different solvents is about −100 kJ mol^−1^ (ΔG^0^_bind_ = −98.6 kJ mol^−1^ (MeCN), −98.3 kJ mol^−1^ (DMSO, coordination through the oxygen atom), −96.5 kJ mol^−1^ (H_2_O), −98.7 kJ mol^−1^ (MeOH), and −103.0 kJ mol^−1^ (THF)). It is reasonable to assume that the resting state of the reaction is **[Ir^III^(Base)]^+^** (Figure 4); thus, the effective concentration of **[Ir^III^(solv)]^+^** in the reaction is very low, contributing to the low TOF. When **M1** is subjected to 1 atm of H_2_ in the presence of TMG (see Sections 5.1 and 5.2 in the electronic supporting information), TMG binds quantitatively at Ir; hence, no **[Ir^III^H]** formation can be observed.

**[Ir^III^(solv)]^+^** reacts with H_2_ to give the intermediate **[Ir^III^(η^2^**-**H_2_)]^+^** (Figure 4). The calculated H-H bond length (92.7 pm) in the side-on coordinated dihydrogen indicates a significant activation considering an H-H bond length of 76.1 pm in molecular hydrogen [43]. The formation of the key intermediate **[Ir^III^H]** proceeds through a heterolytic cleavage of the η^2^-bound H_2_. In the presence of the strong base DBU, **[Ir^III^(η^2^-H_2_)]^+^** seems to be deprotonated instantaneously, as it was not possible to detect the transition state (TS) of the heterolytic cleavage using DFT calculations. Furthermore, the structure of **[Ir^III^(η^2^-H_2_)]^+^** was found to be only stable on the potential energy hypersurface in the absence of base, supporting the idea of a barrierless reaction. Interestingly, a TS for the heterolytic cleavage of H_2_—with a very small barrier—was found when the D3 dispersion correction was omitted, suggesting that the non-covalent interactions between the large complex **[Ir^III^(η^2^-H_2_)]^+^** and a bulky base could decrease and remove the barrier. Furthermore, the counterion PF_6_^−^ was neglected in the DFT calculations, but could also have an effect on the stability of **[Ir^III^(η^2^-H_2_)]^+^** as well as on **[TS1]**.

**Scheme 2:**
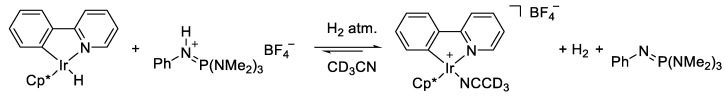
Equilibrium between hydrido complex **[Ir^III^H]** and cationic **[Ir^III^(NCCD_3_)]^+^** used to determine the thermodynamic hydricity [42].

The next step in the catalytic cycle is the hydride transfer from **[Ir^III^H]** to CO_2_. In similar catalytic processes, this is often the overall RDS [27,28,29,30]. However, not only the kinetics of this step bear remarkable consequences for the overall reaction, but also its thermodynamics. By determining the thermodynamic hydricity (ΔG^0^_H−_), the Gibbs free energy change of the hydride transfer from the metal hydride to carbon dioxide can be calculated [44]. Since both hydride complexes **[Rh^III^H]** and **[Ir^III^H]** are isostructural, the hydride transfer has to undergo the same transition state with both metals and thus the Bell–Evans–Polanyi principle can be applied, i.e., the more endergonic reaction will also have a higher transition state [45,46]. The thermodynamic hydricity of **[Rh^III^H]** has been determined by ^1^H-NMR spectroscopy (Scheme 2); using a similar procedure, ΔG^0^_H−_ for **[Ir^III^H]** was calculated [42].

**Figure 5:**
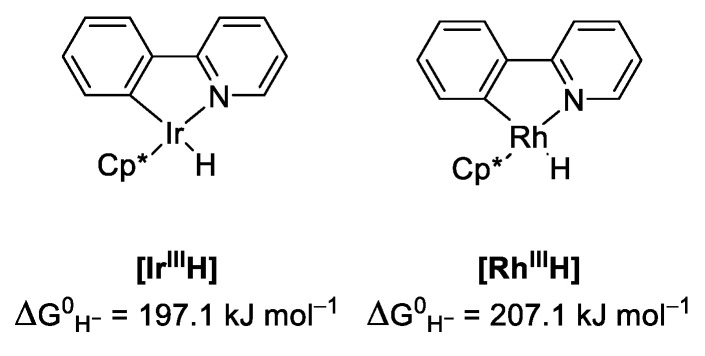
Hydrido complexes **[Ir^III^H]** and **[Rh^III^H]** (Cp* = C_5_Me_5_) with their respective thermodynamic hydricity [42].

While the hydricity of **[Rh^III^H]** could be assessed using a triethylammonium salt, **[Ir^III^H]** was too reactive and reacted quantitatively. Using a less acidic phosphorimidic triamide salt a ΔG_H−_(**[Ir^III^H]**) of 197.1 kJ mol^−1^ was determined (Figure 5).

**Scheme 3:**
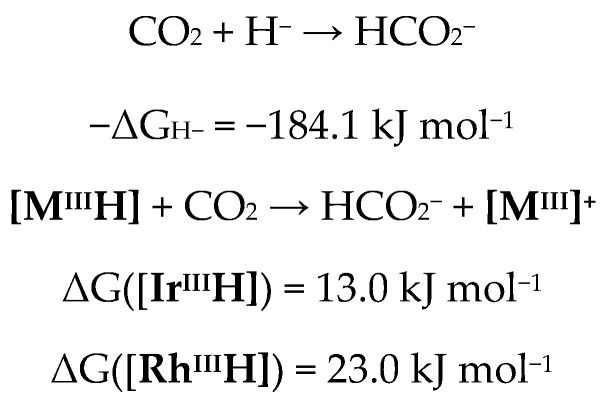
Calculated reaction enthalpies for hydride transfer from **[M^III^H]** to CO_2_.

The relatively high ΔG_H−_ indicates that the hydride transfer from both Ir and Rh to CO_2_ is thermodynamically unfavorable (Scheme 3), and the catalytic activity is most likely due to the basicity of the base (in case of the sufficiently strong bases DBU or TMG), high pressure and temperature [44]. This would also explain the strong pressure dependency of the catalytic activity (Table 1, entry 1 and entry 11). When subjecting **[Ir^III^H]** to 1 atm of CO_2_ in CD_3_CN, no reaction was observed over the course of one week. Furthermore, the hydride transfer from Rh is 10.0 kJ mol^−1^ less favorable than the hydride transfer from Ir, which explains the much higher reactivity of **C1** and **M1**.

The hydride transfer can follow an inner-sphere or outer-sphere mechanism. The outer-sphere reaction would proceed through **[TS2]** to form the H-bound formate adduct [Ir^III^HCO_2_]*. This adduct can either release the formate anion followed by recoordination via oxygen, or **[Ir^III^HCO_2_]** can be formed through an intramolecular isomerization. An inner-sphere mechanism would involve direct binding of CO_2_ to form **[Ir^III^HCO_2_]** directly through **[TS3]**. Both pathways result in the formation of **[Ir^III^HCO_2_]**, which then releases the formate to close the catalytic cycle. When **M1** is mixed in a 1:1 ratio with [N(^n^Bu)_4_]HCO_2_, **[Ir^III^HCO_2_]** is formed exclusively (see Section 5.3 in the electronic supporting information). To further assess whether the liberation of formate could be the overall rate-determining step, phosphorimidic triamide and H_2_ (1 atm) were added to this mixture. While the formation of **[Ir^III^H]** is slower from **[Ir^III^HCO_2_]** than staring directly from **M1**, it can be observed. This shows that the liberation of formate is unfavorable but not rate determining. While the hydrogen activation is only slowed down in the presence of formate, no reaction was observed when **[Ir^III^H]** was subjected to a CO_2_ atmosphere at room temperature. This shows that the hydride transfer to CO_2_ is indeed the RDS of the catalysis.

The complex [M^III^HCO_2_]^*^ and transition state **[TS2]** could not be optimized through DFT calculations, as the structures always spontaneously rearranged into **[M^III^HCO_2_]** and **[TS3]**, respectively. Thus, it is reasonable to assume that the reaction follows an inner-sphere mechanism.

**Figure 6:**
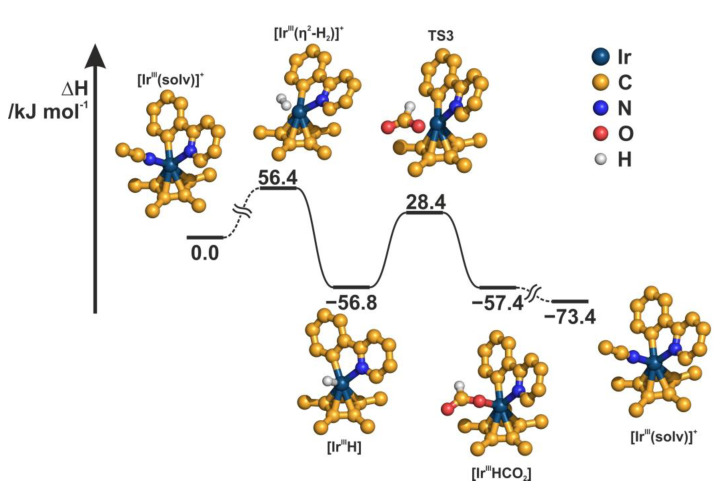
Reaction enthalpy profile for the catalyst **M1** in MeCN using DBU as a base.

Calculating accurate solution phase entropies is a challenge for quantum chemistry, and for the hydrogenation of CO_2_ to formate, two components of the reaction must enter the solution from gas phase at elevated pressures. To avoid discussing this elusive term, we will here consider only the more reliable calculated relative enthalpies; the corresponding Gibbs free energies are given in the electronic supporting information. The reaction enthalpy profile is presented in Figure 6 for **M1** in MeCN and with DBU as base; detailed numerical data for all other variations are summarized in Table 2. The counterion PF_6_^−^ was neglected in all calculations, assuming its influence on the catalyst to be only minor. As discussed above, the coordination of the base or the solvent result in a highly stable resting state. Taking **[Ir^III^(solv)]^+^** as reference, the formation of the complex with an η^2^-coordinated dihydrogen ligand is endothermic for all solvents (MeCN, DMSO, H_2_O and MeOH). Splitting the H-H bond in the before mentioned barrierless step is thermodynamically more favorable with DBU than NEt_3_ as base yielding **[Ir^III^H]**. In agreement with the discussion above, the reaction of the base with the formed formic acid is necessary if the overall catalytic cycle is to be thermodynamically favorable. Accordingly, the formation of a salt consisting of the protonated base and a formate anion was observed experimentally.

Clearly, the values discussed above would be different if entropic effects were considered, since the reaction of H_2_ with **[Ir^III^(solv)]^+^** and of CO_2_ with **[Ir^III^H]** involve the association of molecules. However, since these effects are similar for all the systems that were compared in this study, the trends observed in enthalpies are valid for Gibbs free energies as well.

It should further be noted that all shown solution phase enthalpies were calculated using an implicit continuum solvation model. This approach, while acting as a good foundation for the comparison of different solvents, allows for some inaccuracy for not including explicit solvation.

### 2.5. Identifying the Synergistic Interaction

As the hydride transfer from **[Ir^III^H]** to CO_2_ is the overall RDS, the synergism that enhances the reactivity in **C1** needs to assist in this step. There are several examples of Mo-phosphine complexes capable of binding and activating CO_2_, e.g., for coupling with ethylene forming acrylate. However, all these complexes contain at least one labile ligand [47,48,49,50,51,52]. It is possible that during the activation period, before the addition of CO_2_, one or more CO ligands are cleaved from the Mo center of **C1**. However, considering that no CO ligands were eliminated at high temperature (>110 °C) in the presence of trimethylamine *N*-oxide or under irradiation with UV light, we infer that the Mo complex is stable under the reaction conditions.

In a first attempt to study the activation period of the catalyst (30 bar H_2_ for 30 min), a solution containing **M1** and **M3** was subjected to 1 atm of H_2_ in the presence of excess NEt_3_ (see Section 5.4 in the electronic supporting information). While no transformation on the Mo center of **M3** could be observed in either the ^1^H- or ^31^P-NMR spectra over the course of one week, formation of **[Ir^III^H]** from **M1** occurred. This indicates an interaction in which **M3** assists in the formation of **[Ir^III^H]** and stabilizes the latter. As mentioned before, in the absence of **M3**, HNEt_3_^+^ is too acidic and quantitatively reacts with **[Ir^III^H]**. The exact nature of the interaction between **M1** and **M3** is not yet understood as **M3** does not interact with HNEt_3_^+^, at least not in a manner that is observable by IR or NMR spectroscopy.

When **C1** is subjected to 1 atm of H_2_ in the presence of excess NEt_3_, the formation of an iridium hydride species can be observed as well using ^1^H-NMR spectroscopy. Additionally, a shift in the ^31^P{^1^H}-NMR spectrum takes place supporting the formation of a corresponding hydrido complex, which, however, decomposed over the course of several days. The fact that a transformation occurred while no reaction was observed at the molybdenum center in the mixture of the two monometallic species further confirms that the synergistic effect is much more pronounced in the heterobimetallic complex. Future work is now focused on identifying the intermediate and understanding the synergistic interaction.

## 3. Computational Methods

All calculations were carried out using ORCA (version 3.0) [53] for the complexes **M1** and **M2**. Density functional theory with the exact exchange functional B3LYP and the def2-SVP basis set was used [53,54,55,56]. All calculations utilize the atom-pairwise dispersion correction with the Becke-Johnson damping scheme (D3BJ) [57,58]. The settings TightSCF, grid5, and TightOpt were employed. After each optimization, a numerical frequency calculation was carried out in order to confirm minimum structures on the potential energy surface. For the transition state search the same settings with unrestricted calculations were used, but with the OptTS and the SlowConv keywords. A single-point calculation on each of the structures optimized in the gas phase was performed, using the conductor-like polarizable continuum model (CPCM) for each solvent with the default settings [59].

## 4. Conclusions

Two heterobimetallic complexes and their monometallic counterparts were synthesized, fully characterized, and their catalytic activity was studied in homogeneous CO_2_ hydrogenation. Best results in terms of catalytic activity were obtained with the heterobimetallic Mo,Ir complex **C1** (TON 168, TOF 5 h^−1^). A synergistic effect was observed in all heterobimetallic complexes, leading to a significant increase in reactivity, as compared with the 1:1 mixture of their monometallic counterparts. Electrochemical analysis indicates that this synergism cannot be attributed to an electronic interaction, which is further supported by spectroscopic data.

As the two metal centers in the heterobimetallic complex do not communicate electronically, the monometallic Ir complex **M1** was studied extensively as a model for the Ir center in **C1**. A mechanism for the CO_2_ hydrogenation using **M1** was proposed and is supported by extensive calculations and NMR spectroscopic data. The overall rate-determining step is the hydride transfer from the Ir catalyst to CO_2_. The resting state of the reaction is the base adduct of **M1**, thus removing it from the reaction and leading to low TOF. Since the mechanism of the CO_2_ hydrogenation using **C1** must be similar to the mechanism using **M1,** one can deduce that a synergism assists in the hydride transfer to CO_2_. For further understanding of the heterobimetallic system, the active catalyst must be identified, and the catalytic reaction studied by in situ spectroscopy.

## Figures and Tables

**Table 1:** Catalytic CO_2_ hydrogenation to formate using different complexes and bases (Cp* = C_5_Me_5_).^(a)^

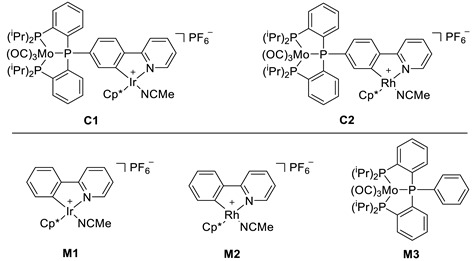					
**Entry**	**Catalyst**	**Catalyst Loading (mol%) ^(b)^**	**Base**	**TON ^(c)^**	**Yield (%) ^(d)^**
1	**C1**	0.6	DBU	128	77
2	**M1**	0.6	DBU	23	14
3	**M3**	0.6	DBU	traces	<1
4	**M1 + M3 ^(e)^**	0.6	DBU	34	21
5	**C2**	0.6	DBU	17	10
6	**M2**	0.6	DBU	5	3
7	**M2 + M3 ^(e)^**	0.6	DBU	3	2
8	**C1**	0.6	TMG	168	Quant.
9	**C1**	0.6	NEt_3_	45	27
10	**C1**	0.6	-	-	0
11	**C1 ^(f)^**	0.6	DBU	12	7

^(a)^ Reaction conditions: catalyst (3 µmol), base (500 µmol), 2 mL total volume of MeCN solution, 50 °C, H_2_ (30 bar), 30 min; CO_2_ (30 bar, total pressure 60 bar), 22 h; DBU = 1,8-diazabicyclo[5.4.0]undec-7-ene, TMG = 1,1,3,3-tetramethylguanidine. ^(b)^ In relation to amount of base. ^(c)^ Based on ^1^H-NMR signal of HCO_2_^−^ relative to the internal standard (benzene in C_6_D_6_ in a capillary). ^(d)^ Determined by n(formate)/n(base) by ^1^H-NMR spectroscopy. ^(e)^ 3 µmol of each complex. ^(f)^ H_2_ (15 bar), CO_2_ (15 bar, total pressure 30 bar).

**Table 2:** Calculated relative enthalpies (DFT, B3LYP-D3/def2-SVP level of theory in kJ mol^−1^) for the catalytic cycle of monometallic Ir complexes for different solvents and bases.

Intermediate.	Base	MeCN (M1)	DMSO	H_2_O	MeOH
**[Ir^III^(solv)]^+^**	DBU	0.0	0.0	0.0	0.0
	NEt_3_	0.0	0.0	0.0	0.0
**[Ir^III^(base)]^+^**	DBU	−66.7	−34.2	−86.2	−84.0
	NEt_3_	−9.0	22.5	−28.7	−26.2
**[Ir^III^(η^2^-H_2_)]^+^**	DBU	56.4	87.7	36.3	39.3
	NEt_3_	56.4	87.7	36.3	39.3
**[Ir^III^H]**	DBU	−56.8	−25.8	−77.6	−73.8
	NEt_3_	−9.0	21.7	−30.4	−25.8
**[TS3]**	DBU	28.4	59.1	7.8	11.6
	NEt_3_	76.3	106.7	55.0	59.6
**[Ir^III^HCO_2_]**	DBU	−57.4	−26.6	−78.7	−74.3
	NEt_3_	−9.6	21.0	−31.5	−26.3
**[Ir^III^(solv)]^+ (a)^**	DBU	−73.4	−73.3	−73.3	−73.4
	NEt_3_	−59.6	−59.7	−59.7	−59.5

^(a)^ With considering the formation of the [DBU(H)]HCO_2_ salt.

## Data Availability

X-ray crystallography data can be accessed through the CCDC (2210067-2210072). All other data are given in the Supplementary Materials.

## Supplementary Materials

The following supporting information can be downloaded at: https://www.mdpi.com/article/10.3390/molecules28062574/s1. Crystallographic data for **M3**, **2**, the starting materials [Mo(CO)_3_;RhCl] (**3**) and [Mo(CO)_3_;IrCl] (**4**), and the side products [Mo(CO)_3_;Rh(CO)]PF_6_ and [Mo(CO)_3_;Ir(N_2_)]PF_6_. Additional data for synthesis, spectroscopic characterizations, catalysis studies, computational studies, hydricity measurements, mechanistic NMR experiments and electrochemical data. References [60,61,62,63,64,65,66,67,68,69,70,71,72,73,74,75,76,77,78] are cited in the Supplementary Materials.
