# Supplementary material for: Synergistic Catalysis in Heterobimetallic Complexes for Homogeneous Carbon Dioxide Hydrogenation
Source: Molecules. 2023 Mar 12;28(6):2574. doi: 10.3390/molecules28062574 (PMC10059594; doi:10.3390/molecules28062574)
Supplement: Supplementary file 1 [file molecules-28-02574-s001.zip › molecules-2257547-supplementary.pdf]

Synergistic Catalysis in Heterobimetallic Complexes for Homogeneous Carbon Dioxide Hydrogenation

Zeno B. G. Fickenscher, Peter Lönnecke, Anna K. Müller[2], Oldamur Hollóczki[3], Barbara Kirchner[2], Evamarie Hey-Hawkins*

Institute of Inorganic Chemistry, Universität Leipzig, Johannisallee 29, D-04103 Leipzig, Germany

[2]Mulliken Center for Theoretical Chemistry, Institute for Physical and Theoretical Chemistry, Beringstr. 4, 53115 Bonn, Germany

[3]Department of Physical Chemistry, Faculty of Science and Technology, University of Debrecen, Egyetem ter 1, H-4010 Debrecen, Hungary

* E-mail: hey@uni-leipzig.de

Web: https://anorganik.chemie.uni-leipzig.de/de/anorganik/ak-hey-hawkins/

Table of Contents

1. General Remarks 1

2. Synthesis 4

2.1. Synthesis of Tris(dimethylamino)(phenylamino)phosphonium tetrafluoroborate 4

2.2. Synthesis of Bis[2-(diisopropylphosphanyl)phenyl]-4-(2-pyridyl)phenyl-phosphane (**1**) . 4

2.3. Synthesis of **M3** 5

2.4. Synthesis of **2** 7

2.5. Synthesis of **[Mo(CO)$_3$;RhCl]** (**3**) 7

2.6. Synthesis of **[Mo(CO)$_3$;IrCl]** (**4**) 9

2.7. Synthesis of **C2** 10

2.8. Synthesis of **C1** 11

3. Catalysis 13

3.1. Procedure 1 13

3.2. Catalysts 14

3.3. Solvent 15

3.4. Base 16

3.5. Reaction time / Determination of TOF 17

4. Determination of Thermodynamic Hydricity of **[Ir^{III}H]** 18

5. Mechanistic ^{1}H NMR Spectroscopic Studies 19

5.1. Coordination of TMG to **M1** 19

5.2. **M1** under H_2 atmosphere 20

5.3. Coordination of HCO_2^- to **M1** and subsequent H_2 activation 22

5.4. H_2 activation by a 1:1 mixture of **M1** and **M3** 24

6. ^{1}H, ^{31}P{^{1}H}, ^{13}C{^{1}H} and ^{19}F{^{1}H} NMR spectra 26

6.1. **1** 26

6.3. **M3** ... 29

6.4. **2** ... 32

6.5. **[Mo(CO)$_3$;RhCl]** (**3**) ... 35

6.6. **[Mo(CO)$_3$;IrCl]** (**4**) ... 38

6.7. **C2** ... 41

6.8. **C1** ... 45

6.9. Representative ^{1}H NMR spectrum (400 MHz, D_2O) of catalytic trials ... 49

6.10. ^{1}H NMR spectrum (400 MHz, CD_3CN) of hydricity measurement of **[Ir^{III}H]** at equilibrium ... 50

7. X-Ray Crystallography and Structure Refinement Details ... 51

8. Electrochemical Data ... 55

8.1. Cyclic Voltammogram of **M3** ... 55

8.2. Cyclic Voltammogram of **M3** and **M1** ... 56

8.3. Cyclic Voltammogram of **M3** and **M2** ... 57

8.4. Cyclic Voltammogram of **C2** ... 58

8.5. Cyclic Voltammogram of **C1** ... 59

9. Gibbs Free Energies ... 59

10. References ... 60

1. General Remarks

Safety advice: *High-pressure experiments with compressed gases represent a significant safety risk and must be conducted only following appropriate safety procedures and in conjunction with the use of suitable equipment.*

Reactions were performed using standard Schlenk line and glovebox techniques. All high pressure reactions were performed using a TAIATSU TECHNO® SUS316 120 CCALL equipped with a TEFLON inlet resulting in a reaction volume of 50 ml and a special made adapter to connect to the Schlenk line. H_2 and CO_2 gas were purchased from ALPHAGAZ and AIR LIQUIDE, respectively, and used without further purification. Reagents were purchased from chemical vendors and used without further purification unless noted otherwise. PCl_3 was degassed, distilled, and stored under nitrogen atmosphere over 4 Å molecular sieve prior to use. $NaPF_6$, LiOH, Li_2CO_3, Cs_2CO_3 were dried at 150 °C under reduced pressure for 18 h and stored under nitrogen atmosphere. $[RhCp^*Cl_2]_2$ and $[IrCp^*Cl_2]_2$ ($Cp^* = C_5Me_5$) were synthesized according to literature procedure as were **M1**, **M2**, bis(2-diisopropylphosphinophenyl)chlorophosphine, bis[2-(diisopropylphosphanyl)phenyl] phenylphosphane and phenyl-tris(dimethylamino)phosphorane [1–7]. The NMR spectra were recorded at 25 °C, unless noted otherwise, with a Bruker AVANCE DRX 400 spectrometer (^{1}H NMR: 400.13 MHz, ^{13}C NMR: 100.16 MHz, ^{31}P NMR: 161.97 MHz, ^{19}F NMR: 376.53 MHz). TMS was used as internal standard for ^{1}H and ^{13}C NMR spectra, and all spectra of other nuclei were referenced to TMS by using the Ξ scale [8]. In the ^{13}C NMR spectra of **3, 4, C1** and **C2** several signals corresponding to quaternary carbon atoms and particularly carbon atoms directly adjacent to phosphorus are not observable. Even when a 90° pulse sequence with extended measurement time (~ mass spectra were recorded on a BRUKER DALTONICS Impact II ESI-TOF mass spectrometer or SHIMADZU TQ 8040 GC-EI Quadrupole MS. IR spectra were measured between 4000 bis 400 cm^{-1}. The measurement of ATR-IR spectra was done using a THERMOFISHER NICOLET iS5 ATR spectrometer, KBr pellets were measured on a FT-IR SPEKTRUM 2000 spectrometer by PERKINELMER. All solvents were purified and degassed with an MBRAUN Solvent Purification System SPS-800. Dichloromethane (DCM) was stored under nitrogen over 4 Å molecular sieve. Diethyl ether (Et_2O) was stored under nitrogen over potassium mirror. Tetrahydrofuran (THF) was distilled over K and benzophenone under nitrogen atmosphere and stored over 4 Å molecular sieve. Methanol (MeOH) was dried over Mg, distilled,

stored under N_2 atmosphere over 3 Å molecular sieve. Dimethyl sulfoxide (DMSO) was dried over CaH_2 for 6 h, distilled under reduced pressure over CaH_2 and stored under N_2 atmosphere over 4 Å molecular sieve. Acetonitrile (MeCN) was dried over P_2O_5 for 3 d, distilled over P_2O_5, stored under N_2 atmosphere and over 3 Å molecular sieve. *n*-Pentane and *n*-hexane were stored over potassium under nitrogen atmosphere. All deuterated solvents (CD_2Cl_2, C_6D_6, CD_3CN, DMSO-d_6, THF-d_8) except D_2O were dried over 3 Å molecular sieve, degassed using freeze-pump-thaw and stored under N_2 atmosphere over 3 Å molecular sieve. Triethylamine (NEt_3) and *N,N*-diisopropylethylamine (DIPEA) were stirred over lithium aluminum hydride for 3 d, distilled and stored over 3 Å molecular sieve under nitrogen atmosphere with exclusion of light. Tetramethylguanidine (TMG) and 1,8-diazabicyclo[5.4.0]undec-7-ene (DBU) were directly stored over 3 Å molecular sieve under nitrogen atmosphere after obtaining the chemicals from the vendor. Crystallographic data were collected on a GEMINI-CCD diffractometer (RIGAKU OXFORD DIFFRACTION) using Mo-Kα radiation (λ = 71.073 pm), ω-scan rotation. Data reduction was performed with CRYSALIS PRO including the program SCALE3 ABSPACK for empirical absorption correction and an analytical numeric absorption correction using a multifaceted crystal model based on expressions derived by R.C. CLARK & J.S. REID [9,10]. The structure was solved by dual-space methods with SHELXT-2018 and the refinement of all non-hydrogen atoms was performed with SHELXL-2018 [11]. All non-hydrogen atoms were refined with anisotropic thermal parameters and the HFIX command was used to locate all hydrogen atoms for non-disordered regions of the structure. Cyclic voltammetry (CV) measurements on ~1.0 $mmol \cdot l^{-1}$ analyte solutions in dry, oxygen-free MeCN containing 0.1 $mol \cdot l^{-1}$ tetrabutylammonium hexafluorophosphate as supporting electrolyte have been conducted in a three-electrode setup (GAMRY INSTRUMENTS, SP-50 POTENTIOSTAT by BIOLOGIC SCIENCE INSTRUMENTS) under a blanket of nitrogen at room temperature. The glassy-carbon working electrode (ALS; surface area 0.07 cm^2) and the counter electrode (NEOLAB; platinum wire, 99.9%) were immersed in the analyte solution, while the reference electrode (ALS; Ag/$AgNO_3$ (0.01 $mol \cdot l^{-1}$) in 0.1 $mol \cdot l^{-1}$ tetrabutylammonium hexafluorophosphate in dry, oxygen-free CH_3CN) was connected to the cell via a bridge tube (filled with the supporting electrolyte) through VYCOR TIPS. The reference electrode was calibrated against decamethylferrocene as an internal standard at the end of the CV experiment, and the results were converted to the FcH/$[FcH]^+$ scale in accordance with the IUPAC

requirements [12,13]. The concentration of organo-lithium compounds was determined through titration with N-benzylbenzamide, according to BURCHART [14].

2. Synthesis

2.1. Synthesis of Tris(dimethylamino)(phenylamino)phosphonium Tetrafluoroborate

Tris(dimethylamino)(phenylimino)phosphorane (1.33 g, 5.24 mmol, 1.0 equiv.) was dissolved in THF (5 ml) and cooled to 0 °C. HBF_4 (0.47 ml of a 11.2 M aqueous sol., 5.24 mmol, 1.0 equiv.) was added dropwise. The mixture was stirred for 10 min. The solvent was removed under reduced pressure and the residue dried in vacuum (< 1 × 10^{-2} mbar) for 18 h. The residue was triturated, washed with Et_2O (3 × 2 ml) and dried under vacuum (< 1 ×10^{-2} mbar) for 5 h. The product was obtained as a yellow waxy solid (1.57 g, 4.58 mmol, 87%).

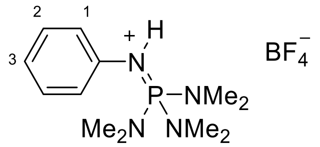

^{1}H NMR (400.1 MHz, CD_3CN): δ = 2.71 (d, $^3J_{HP}$ = 10.0 Hz, 18 H, NMe_2), 6.97 (d, $^3J_{HH}$ = 8.0 Hz, 2 H, 1-H), 7.05 (t, $^3J_{HH}$ = 7.4 Hz, 1 H, 3-H), 7.30 (ddd, $^3J_{HH}$ = 7.5 Hz, $^3J_{HH}$ = 7.5 Hz, $^3J_{HH}$ = 0.9 Hz, 2 H, 2-H) ppm. ^{31}P{^{1}H} NMR (161.97 MHz, CD_3CN): δ = 32.4 (s) ppm.

2.2. Synthesis of Bis[2-(diisopropylphosphanyl)phenyl]-4-(2-pyridyl)phenyl-phosphane (**1**)

Mg turnings (0.61 g, 25 mmol, 1.1 equiv.) were put in a flame-dried flask and heated to 200 °C under vacuum for two hours. The flask was cooled to room temperature and the Mg turnings covered with THF (5 ml). 2-(4-Bromophenyl)pyridine (5.15 g, 22 mmol, 1.0 equiv.) was dissolved in THF (60 ml) and added dropwise over 40 min under reflux. The mixture was stirred under reflux for 5 h. The concentration of the Grignard reagent solution was determined according to literature [15]. Bis(2-diisopropylphosphinophenyl)chlorophosphine (8.73 g, 19.3 mmol, 1.0 equiv.) was dissolved in Et_2O (100 ml), cooled to −80 °C and the freshly prepared Grignard reagent solution (60 ml of 0.32 M in THF sol., 19.3 mmol, 1.0 equiv.) was added dropwise. The solution was warmed to room temperature over night. Degassed water (30 ml) was added to the solution, the layers were separated, and the organic layer was washed with brine (2 × 10 ml). The aqueous layers were extracted with Et_2O (2 × 20 ml) and the combined organic layers were dried over $MgSO_4$. The solvent was removed, the residue triturated with Et_2O (30 ml) and washed with MeCN (60 ml) and MeOH (20 ml). The resulting white powder was dried under vacuum over

night. The product was obtained as a white powder (3.95 g, 10.4 mmol, 54%). Precipitation of the filtrate, at −20 °C over 2 months yielded additional product (1.13 g, 1.9 mmol, total yield: 64%)

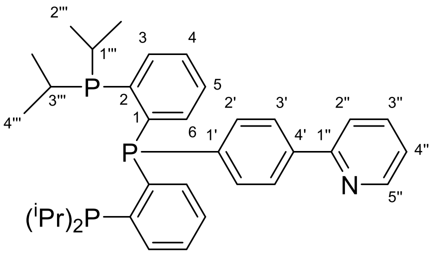

^{1}H NMR (400.1 MHz, CD_2Cl_2): δ = 0.83 (ddd, $^{3}J_{HP}$ = 13.8 Hz, $^{3}J_{HH}$ = 12.0, $^{3}J_{HH}$ = 7.0 Hz, 12 H, 2'''-H), 1.11 (ddd, $^{3}J_{HP}$ = 13.8 Hz, $^{3}J_{HH}$ = 7.0 Hz, $^{3}J_{HH}$ = 4.5 Hz, 12 H, 4'''-H), 2.09 (heptd, $^{3}J_{HH}$ = 7.0 Hz, $^{2}J_{HP}$ = 2.2 Hz, 2 H, 3'''-H), 2.18 (heptd, $^{3}J_{HH}$ = 7.0 Hz, $^{2}J_{HP}$ = 2.9 Hz, 2 H, 1'''-H), 6.79 – 6.85 (m, 2 H, 3-H), 7.17 (pt, ^{3}J$_{HH}$ = 7.5 Hz, 2 H, 5-H), 7.22 (m, 1 H, 4''-H), 7.25 (dd, $^{3}J_{HH}$ = 8.2, J_{HP} = 6.2 Hz, 2 H, 2'-H), 7.31 (td, ^{3}J$_{HH}$ = 7.5 Hz, $^{4}J_{HH}$ = 1.4 Hz, 2 H, 4-H), 7.52 (ddpt, $^{3}J_{HH}$ = 7.4 Hz, $^{4}J_{HH}$ = 1.7 Hz, $^{3}J_{HP}$ = 3.5 Hz, 2 H, 6-H), 7.72 - 7.78 (m, 2 H, 3''+2''-H), 7.91 - 7.96 (m, 2 H, 3'-H), 8.65 (dpt, $^{3}J_{HH}$ = 4.8 Hz, $^{4}J_{HH}$ = 1.4 Hz, 1 H, 5''-H) ppm. ^{13}C{^{1}H} NMR (100.6 MHz, CD_2Cl_2): δ = 19.5 (d, $^{2}J_{CP}$ = 10.8 Hz, $C^{2'''}$), 20.0 (m, $C^{4'''}$), 24.6 (ddd, $^{1}J_{CP}$ = 32.6 Hz, $^{1}J_{CP}$ = 14.1 Hz, $^{1}J_{CP}$ = 4.5 Hz, $C^{1'''}$), 25.6 (s, $C^{2'''}$), 120.3 (s, $C^{4''}$), 122.2 (s, $C^{3''}$), 126.3 (d, $^{3}J_{CP}$ = 6.4 Hz, $C^{3'}$), 127.8 (s, C^{4}), 128.6 (s, C^{5}), 132.2 (d, $^{2}J_{CP}$ = 6.0 Hz, C^{6}), 134.5 (m, C^{3}), 134.9 (d, $^{2}J_{CP}$ = 19.9 Hz, $C^{2'}$), 136.7 (s, $C^{4'}$), 138.8 (m, $C^{1'}$), 142.6 (m, C^{2}), 146.8 (m, C^{1}), 149.5 (s, $C^{5''}$) 156.8 (s, $C^{1''}$) ppm. ^{31}P{^{1}H} NMR (161.97 MHz, CD_2Cl_2): δ = –15.8 (dd, J_{PP} = 158 Hz, J_{PP} = 148 Hz, P(aryl)$_3$), –1.4 (dd, J_{PP} = 152 Hz, J_{PP} = 4 Hz, P(^{i}Pr)$_2$) ppm. HR-MS: ($C_{35}H_{44}NP_3$; $[M+H]^+$, pos. ESI) calcd.: 572.277, found: 572.274.

2.3. Synthesis of **M3**

[Mo(CO)$_3$(MeCN)$_3$] (34 mg, 0.11 mmol, 1.0 equiv.) and bis[2-(diisopropylphosphanyl)phenyl]phenylphosphane (55 mg, 0.11 mmol, 1.0 equiv.) were dissolved in THF (2 ml). The mixture was stirred at 60 °C over night. The solvent was removed, the residue washed with *n*-pentane (3 × 2 ml). The residue was dried under vacuum. The product was obtained as a yellow powder (86 mg, 0.12 mmol, quant.). Single crystals were obtained by slow evaporation of solvent from a saturated benzene solution.

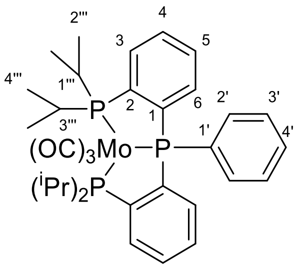

^{1}H NMR (400.1 MHz, C_6D_6): δ = 0.31 (dd, $^3J_{HP}$ = 13.6 Hz, $^3J_{HH}$ = 6.9 Hz, 6 H, 2'''-H^A), 0.53 (dd, $^3J_{HP}$ = 13.7 Hz, $^3J_{HH}$ = 7.0 Hz, 6 H, 4'''-H^A), 0.79 (dd, $^3J_{HP}$ = 13.0 Hz, $^3J_{HH}$ = 7.0 Hz, 6 H, 4'''-H^B), 0.97 (dd, $^3J_{HP}$ = 17.0 Hz, $^3J_{HH}$ = 6.9 Hz, 6 H, 2'''-H^B), 1.69 (m_c, 2 H, 3'''-H), 2.18 (m_c, 2 H, 1'''-H), 6.61 (m_c, 4 H), 6.74 (td, $^3J_{HH}$ = 7.3 Hz, $^4J_{HH}$ = 1.6 Hz, 2 H), 6.86 (s, 1 H), 6.91 – 6.96 (m, 2 H) 7.18 (t, $^3J_{HH}$ = 6.8 Hz, 2 H, 5-H), 7.33 (m_c, 2 H) ppm. ^{13}C{^{1}H} NMR (100.6 MHz, C_6D_6): δ = 19.1 (br.s., $C^{2'''A}$), 19.3 (br.s., $C^{4'''A}$), 20.3 (s, $C^{4'''B}$), 20.4 (s, $C^{2'''B}$), 28.8 (m_c, $C^{3'''}$), 29.9 (t, $^1J_{CP}$ = 11.5 Hz, $C^{1'''}$), 128.6 (s, C^{Ar}), 128.7 (s, C^6), 128.8 (s, C^{Ar}), 129.0 (d, $^2J_{CP}$ = 4.6 Hz, C^{Ar}), 129.4 (s, C^{Ar}), 130.4 (s, C^{Ar}), 130.6 (s, C^{Ar}), 132.6 (s, C^{Ar}), 132.7 (s, C^{Ar}), 132.8 (t, $^2J_{CP}$ = 5.9 Hz, C^{Ar}), 137.5 (m, $C^{1'}$), 143.7 (m, C^1), 146.8 (m, C^2), 222.2 (s, CO) ppm. ^{31}P{^{1}H} NMR (161.97 MHz, C_6D_6): δ = 72.1 (d, J_{PP} = 7.8 Hz, P(^{i}Pr)$_2$), 80.0 (t, J_{PP} = 7.8 Hz, P(aryl)$_3$) ppm. IR (KBr), selected vibrations: 1928 (s, Mo-CO), 1847 (s, Mo-CO), cm^{-1}. HR-MS: ($C_{33}H_{41}KMoO_3P_3$; $[M+K]^+$, pos. ESI) calcd.: 709.097, found: 709.101.

2.4. Synthesis of **2**

[$Mo(CO)_3(MeCN)_3$] (357 mg, 1.17 mmol, 1.0 equiv.) and **1** (670 mg, 1.17 mmol, 1.0 equiv.) were dissolved in THF (12 ml). The mixture was stirred under reflux over night. Then the solvent was removed, the residue washed with *n*-pentane (3 × 3 ml) and Et_2O (2 ml) and dried under vacuum. The product was obtained as a yellow powder (978 mg, 1.17 mmol, quant.). Single crystals were obtained by vapor diffusion of *n*-hexane into a saturated benzene solution.

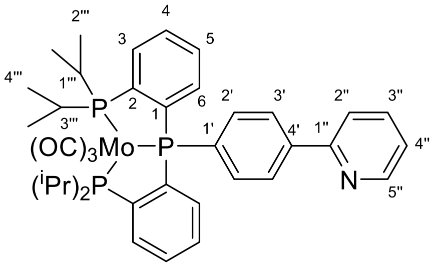

^{1}H NMR (400.1 MHz, C_6D_6): δ = 0.60 (dd, $^{3}J_{HP}$ = 14.0 Hz, $^{3}J_{HH}$ = 6.9 Hz, 6 H, 2’’’-H^{A}), 0.83 (dd, $^{3}J_{HP}$ = 13.6 Hz, $^{3}J_{HH}$ = 7.0 Hz, 6 H, 4’’’-H^{A}), 1.09 (dd, $^{3}J_{HP}$ = 12.8 Hz, $^{3}J_{HH}$ = 6.8 Hz, 6 H, 4’’’-H^{B}), 1.27 (dd, $^{3}J_{HP}$ = 16.4 Hz, $^{3}J_{HH}$ = 6.9 Hz, 6 H, 2’’’-H^{B}), 2.00 (hep, $^{3}J_{HH}$ = 7.0 Hz, 2 H, 3’’’-H), 2.49 (hep, $^{3}J_{HH}$ = 7.1 Hz, 2 H, 1’’’-H), 6.60 (dd, $^{3}J_{HH}$ = 7.4 Hz, $^{3}J_{HH}$ = 4.8 Hz, 1 H, 4’’-H), 6.88 – 6.95 (m, 4 H, 3 + 5-H), 7.02 (td, $^{3}J_{HH}$ = 7.8 Hz, $^{4}J_{HH}$ = 1.8 Hz, 1 H, 3’’-H), 7.15 (m$_{c}$, 1 H, 2’’-H), 7.23 (m$_{c}$, 2 H, 6-H), 7.57 (t, $^{3}J_{HH}$ = 6.8 Hz, 2 H, 4-H), 7.77 (dd, $^{3}J_{HP}$ = 10.2 Hz, $^{3}J_{HH}$ = 8.3 Hz, 2 H, 2’-H), 8.07 (dd, $^{3}J_{HH}$ = 8.4 Hz, $^{3}J_{HP}$ = 2.0 Hz, 2 H, 3’-H), 8.52 (dd, $^{3}J_{HH}$ = 5.0 Hz, $^{4}J_{HH}$ = 1.8 Hz, 1 H, 5’’-H) ppm. ^{13}C{^{1}H} NMR (100.6 MHz, C_6D_6): δ = 18.7 (s, C$^{2'''A}$), 18.9 (s, C$^{4'''A}$), 19.9 (s, C$^{4'''B}$), 20.0 (t, $^{2}J_{CP}$ = 4.5 Hz, C$^{2'''B}$), 28.4 (m$_{c}$, C$^{3'''}$), 29.6 (t, $^{1}J_{CP}$ = 10.5 Hz, C$^{1'''}$), 120.1 (s, C$^{2''}$), 121.9 (s, C$^{4''}$), 126.9 (d, $^{2}J_{CP}$ = 9.4 Hz, C$^{3'}$), 128.5 (s, C^{3}), 128.7 (s, C^{5}), 130.0 (d, $^{2}J_{CP}$ = 15 Hz, C^{6}), 132.6 (m$_{c}$, C$^{1'}$), 132.8 (s, C$^{2'}$), 135.9 (s, C$^{3''}$), 140.1 (s, C$^{4'}$), 143.9 (ddd, $^{1}J_{CP}$ = 10.3 Hz, $^{2}J_{CP}$ = 21.6 Hz, $^{4}J_{CP}$ = 38.3 Hz, C^{2}), 146.7 (ddd, $^{1}J_{CP}$ = 14.0 Hz, $^{2}J_{CP}$ = 17.5 Hz, $^{4}J_{CP}$ = 34.0 Hz, C^{1}) 149.6 (s, C$^{5''}$), 156.2 (s, C$^{1''}$), 225.3 (s, CO), 221.97 (d, $^{2}J_{CP}$ = 6.5 Hz, CO) ppm. ^{31}P{^{1}H} NMR (161.97 MHz, C_6D_6): δ = 72.0 (d, J_{PP} = 8.0 Hz, P(^{i}Pr)$_2$), 79.2 (t, J_{PP} = 8.0 Hz, P(aryl)$_3$) ppm. IR (KBr), selected vibrations: 1931 (s, Mo-CO), 1845 (s, Mo-CO) cm^{-1}. HR-MS: ($C_{38}H_{44}KMoNO_3P_3$; [M+K]$^{+}$, pos. ESI) calcd.: 786.123, found: 786.122.

2.5. Synthesis of **[Mo(CO)$_3$;RhCl]** (**3**)

2 (75 mg, 0.1 mmol, 1.0 equiv.), [$RhCp^*Cl_2$]$_2$ (31 mg, 0.05 mmol, 0.5 equiv.) and NaOAc (25 mg, 0.3 mmol, 3.0 equiv.) were dissolved in DCM (5 ml) and stirred for 5 h. The mixture was filtered

over celite, the solvent was removed, the residue washed with *n*-pentane (3 × 3 ml) and dried under vacuum. The product was obtained as a yellow powder (78 mg, 0.08 mmol, 76%). Single crystals were obtained by vapor diffusion of *n*-pentane into a saturated DCM solution.

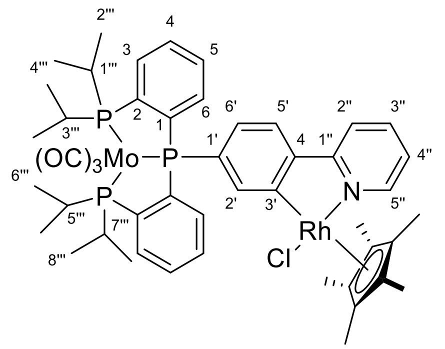

^{1}H NMR (400.1 MHz, CD_2Cl_2): δ = 0.56 (dd, $^3J_{HP}$ = 13.7 Hz, $^3J_{HH}$ = 6.9 Hz, 3 H, 2'''-H^A), 0.83 (dd, $^3J_{HP}$ = 13.5 Hz, $^3J_{HH}$ = 6.9 Hz, 3 H, 4'''-H^A), 0.98 (dd, $^3J_{HP}$ = 13.7 Hz, $^3J_{HH}$ = 7.1 Hz, 3 H, 2'''-H^B), 1.04 (dd, $^3J_{HP}$ = 14.7 Hz, $^3J_{HH}$ = 7.2 Hz, 3 H, 4'''-H^B), 1.11 (dd, $^3J_{HP}$ = 13.4 Hz, $^3J_{HH}$ = 6.9 Hz, 6 H, 6'''-H), 1.25 (dd, $^3J_{HP}$ = 11.0 Hz, $^3J_{HH}$ = 6.1 Hz, 3 H, 8'''-H^A), 1.31 (dd, $^3J_{HP}$ = 10.5 Hz, $^3J_{HH}$ = 6.8 Hz, 3 H, 8'''-H^B), 1.46 (s, 15 H, Cp*), 2.13 (m_c, 1 H, 1'''-H), 2.21 (m_c, 1 H, 3'''-H), 2.97 – 2.80 (m, 2 H, 5''' & 7'''-H), 7.20 (pq, $^3J_{HH}$ = 1.8 Hz, 1 H), 7.22 (dt, $^3J_{HH}$ = 4.8 Hz, $^4J_{HH}$ = 1.8 Hz, 1 H), 7.36 (ddt, $^3J_{HH}$ = 9.4 Hz, $^3J_{HH}$ = 7.9 Hz, $^3J_{HH}$ = 6.4 Hz, 2 H), 7.42 – 7.46 (m, 2 H), 7.57 (tt, $^3J_{HH}$ = 6.8 Hz, $^4J_{HH}$ = 1.7 Hz, 1 H), 7.63 – 7.65 (m, 2 H), 7.71 (m_c, 1 H), 7.75 – 7.83 (m, 4 H), 8.73 (dd, $^3J_{HH}$ = 5.6 Hz, $^4J_{HH}$ = 1.4 Hz, 1 H, 5''-H) ppm. ^{13}C{^{1}H} NMR (100.6 MHz, CD_2Cl_2): δ = 9.3 (s, Cp*-*Me*), 19.3 (d, $^2J_{CP}$ = 3.4 Hz $C^{2'''A}$), 19.5 (s, $C^{4'''A}$), 20.3 (s, $C^{6'''A}$), 20.4 (s, $C^{8'''A}$), 20.5 (d, $^2J_{CP}$ = 2.6 Hz, $C^{8'''B}$), 20.6 (s, $C^{2'''B}$), 21.0 (s, $C^{6'''A}$), 21.1 (s, $C^{4'''B}$), 28.7 (d, $^1J_{CP}$ = 9.3 Hz, $C^{3'''}$), 29.1 (d, $^1J_{CP}$ = 9.3 Hz, $C^{1'''}$), 29.3 (d, $^1J_{CP}$ = 13.4 Hz, $C^{5'''}$), 29.6 (d, $^1J_{CP}$ = 12.7 Hz, $C^{7'''}$), 96.4 (d, $^1J_{CRh}$ = 4.3 Hz, *Cp**-Me), 120.0 (s), 122.9 (s), 123.3 (s, $C^{4''}$), 123.5 (s), 127.9 (s), 128.2 (s), 129.3 (d, J_{CP} = 15.7 Hz), 129.6 (d, J_{CP} = 4.3 Hz), 129.8 (d, J_{CP} = 3.2 Hz), 130.8 (d, J_{CP} = 14.1 Hz), 131.2 (d, J_{CP} = 14.3 Hz), 133.4 (d, J_{CP} = 11.9 Hz), 137.6 (s, $C^{3''}$), 140.0 (d, J_{CP} = 5.0 Hz), 145.0 (s, $C^{4'}$), 151.6 (s, $C^{5''}$), 164.9 (s, $C^{1''}$), 179.1 (d, $C^{3'}$) ppm. ^{31}P{^{1}H} NMR (161.97 MHz, CD_2Cl_2): δ = 69.7 (d, J_{PP} = 23.8 Hz, P(^{i}Pr)$_2$), 71.5 (d, J_{PP} = 24.2 Hz, P(^{i}Pr)$_2$), 78.0 (t, J_{PP} = 7.3 Hz, P(aryl)$_3$) ppm. IR (KBr), selected vibrations: 1931 (s, Mo-CO), 1843 (s, Mo-CO) cm^{-1}. HR-MS: ($C_{48}H_{59}MoNO_3P_3Rh$; [M−Cl]$^+$, pos. ESI) calcd.: 990.173, found: 990.179.

2.6. Synthesis of **[Mo(CO)$_3$;IrCl] (4)**

2 (812 mg, 1.08 mmol, 1.0 equiv.), [IrCp*Cl$_2$]$_2$ (430 mg, 0.54 mmol, 0.5 equiv.) and NaOAc (266 mg, 3.3 mmol, 3.0 equiv.) were dissolved in DCM (20 ml) and stirred over night. The mixture was filtered over celite, the solvent was removed, the residue washed with *n*-pentane (3 × 10 ml) and dried under vacuum. The product was obtained as a yellow powder (1.15 g, 1.03 mmol, 96%). Single crystals were obtained by vapor diffusion of *n*-pentane into a saturated DCM solution.

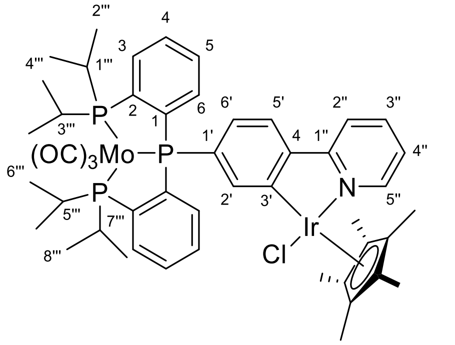

^{1}H NMR (400.1 MHz, CD$_2$Cl$_2$): δ = 0.56 (dd, $^3J_{HP}$ = 13.7 Hz, $^3J_{HH}$ = 6.9 Hz, 3 H, 2’’’-H^A), 0.82 (dd, $^3J_{HP}$ = 13.6 Hz, $^3J_{HH}$ = 6.9 Hz, 3 H, 4’’’-H^A), 0.98 (dd, $^3J_{HP}$ = 13.8 Hz, $^3J_{HH}$ = 7.1 Hz, 3 H, 2’’’-H^B), 1.04 (dd, $^3J_{HP}$ = 14.7 Hz, $^3J_{HH}$ = 7.2 Hz, 3 H, 4’’’-H^B), 1.09 (dd, $^3J_{HP}$ = 13.3 Hz, $^3J_{HH}$ = 6.9 Hz, 3 H, 6’’’-H^A), 1.11 (dd, $^3J_{HP}$ = 13.3 Hz, $^3J_{HH}$ = 7.0 Hz, 3 H, 6’’’-H^B), 1.25 (dd, $^3J_{HP}$ = 9.5 Hz, $^3J_{HH}$ = 6.8 Hz, 3 H, 8’’’-H^A), 1.30 (dd, $^3J_{HP}$ = 9.5 Hz, $^3J_{HH}$ = 6.8 Hz, 3 H, 8’’’-H^B), 1.51 (s, 15 H, Cp*), 2.13 (m$_c$, 1 H, 1’’’-H), 2.21 (m$_c$, 1 H, 2’’’-H), 2.67 – 2.79 (m, 2 H, 5’’’ & 7’’’-H), 7.13 – 7.19 (m, 2 H), 7.37 (m$_c$, 2 H), 7.40 – 7.46 (m, 2 H), 7.58 (m$_c$, 1 H), 7.62 – 7.66 (m, 1 H), 7.69 – 7.82 (m, 6 H), 8.69 (dt, $^3J_{HH}$ = 5.5 Hz, $^4J_{HH}$ = 1.3 Hz, 1 H, 5’’-H) ppm. ^{13}C{^{1}H} NMR (100.6 MHz, CD$_2$Cl$_2$): δ = 8.5 (s, Cp*-*Me*), 18.8 (d, $^2J_{CP}$ = 2.3 Hz C$^{2’’’A}$), 19.0 (s, C$^{4’’’A}$), 19.1 (s, C$^{6’’’A}$), 19.8 (s, C$^{8’’’A}$), 19.9 (s, C$^{8’’’B}$), 20.0 (s, C$^{6’’’B}$), 20.0 (s, C$^{6’’’B}$), 20.6 (s, C$^{4’’’B}$), 28.1 (d, $^1J_{CP}$ = 8.0 Hz, C$^{3’’’}$), 28.5 (d, $^1J_{CP}$ = 9.8 Hz, C$^{1’’’}$), 29.0 (d, $^1J_{CP}$ = 11.0 Hz, C$^{5’’’}$), 29.1 (d, $^1J_{CP}$ = 11.5 Hz, C$^{7’’’}$), 88.5 (s, *Cp**-Me), 119.3 (s), 122.7 (s), 123.2 (s, C$^{4’’}$), 123.4 (s), 126.6 (s), 128.7 (s), 129.0 (br.s), 129.2 (d, J_{CP} = 4.8 Hz), 130.3 (d, J_{CP} = 5.3 Hz), 130.6 (d, J_{CP} = 13.6 Hz), 132.8 (d, J_{CP} = 12.0 Hz), 133.4 (d, J_{CP} = 12.2 Hz), 137.0 (s, C$^{3’’}$), 138.5 (d, J_{CP} = 4.5 Hz), 145.1 (s, C$^{4’}$), 151.2 (s, C$^{5’’}$), 166.4 (s, C$^{1’’}$) ppm. ^{31}P{^{1}H} NMR (161.97 MHz, CD$_2$Cl$_2$): δ = 69.8 (d, J_{PP} = 24.5 Hz, P(^{i}Pr)$_2$), 71.5 (d, J_{PP} = 20.3 Hz, P(^{i}Pr)$_2$), 77.8 (t, J_{PP} = 7.4 Hz, P(aryl)$_3$) ppm. IR (KBr), selected vibrations: 1928 (s, Mo-CO), 1841 (s, Mo-CO) cm^{-1}. HR-MS: (C$_{48}$H$_{59}$IrMoNO$_3$P$_3$; [M−Cl]$^+$, pos. ESI) calcd.: 1080.231, found: 1080.231.

2.7. Synthesis of **C2**

3 (184 mg, 0.18 mmol, 1.0 equiv.) and $NaPF_6$ (30 mg, 0.18 mmol, 1.0 equiv.) were dissolved in MeCN (5 ml) and stirred for 6 h under reflux. The mixture was filtered over celite, the solvent was removed, the residue washed with *n*-pentane (3 × 3 ml) and dried under vacuum. The product was obtained as a yellow powder (202 mg, 0.17 mmol, 96%). Single crystals were obtained by diffusion of *n*-pentane into a saturated THF solution, over the course of 3 months. In the obtained crystals, MeCN was displaced by CO, most likely released as a decomposition product.

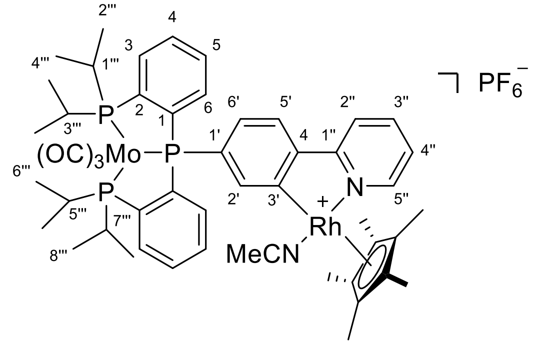

^{1}H NMR (400.1 MHz, CD_3CN): δ = 0.60 (dd, $^{3}J_{HP}$ = 12.9 Hz, $^{3}J_{HH}$ = 6.9 Hz, 3 H, 2’’’-H^A), 0.79 (dd, $^{3}J_{HP}$ = 13.0 Hz, $^{3}J_{HH}$ = 6.9 Hz, 3 H, 4’’’-H^A), 0.97 – 1.13 (m, 12 H, 2’’’, 4’’’, 6’’’, 8’’’-H^B), 1.21 (dd, $^{3}J_{HP}$ = 16.7 Hz, $^{3}J_{HH}$ = 6.8 Hz, 3 H, 6’’’-H^A), 1.35 (dd, $^{3}J_{HP}$ = 17.0 Hz, $^{3}J_{HH}$ = 6.8 Hz, 3 H, 8’’’-H^A), 1.45 (s, 15 H, Cp*), 1.96 (s, 3 H, MeCN), 2.07 (m_c, 1 H, 1’’’-H), 2.20 (m_c, 1 H, 3’’’-H), 2.77 (m_c, 1 H, 5’’’-H), 2.91 (m_c, 1 H, 7’’’-H), 6.98 (ddd, $^{3}J_{HH}$ = 9.7 Hz, $^{3}J_{HH}$ = 8.0 Hz, $^{3}J_{HH}$ = 1.6 Hz, 1 H, 6’-H), 7.41 (ddd, $^{3}J_{HH}$ = 6.6 Hz, $^{3}J_{HH}$ = 5.6 Hz, $^{3}J_{HH}$ = 2.4 Hz, 1 H, 4’’-H), 7.52 – 7.56 (m, 4 H), 7.62 (dd, $^{3}J_{HH}$ = 10.8 Hz, $^{4}J_{HH}$ = 1.6 Hz, 1 H, 2’-H), 7.71 (dd, $^{3}J_{HH}$ = 8.0 Hz, $^{4}J_{HH}$ = 2.0 Hz, 1 H, 5’-H), 7.82 – 7.86 (m, 3 H), 7.91 – 7.99 (m, 3 H), 8.74 (dt, $^{3}J_{HH}$ = 5.6 Hz, $^{4}J_{HH}$ = 1.3 Hz, 1 H, 5’’-H) ppm. ^{13}C{^{1}H}{^{31}P} NMR (100.6 MHz, CD_3CN): δ = 1.32 (s, *Me*CN), 9.3 (s, Cp*-*Me*), 19.6 (s, $C^{2'''A}$), 19.7 (s, $C^{4'''A}$), 19.9 (s, $C^{6'''B}$), 20.1 (s, $C^{8'''B}$), 20.6 (s, $C^{8'''A}$) 21.1 (s, $C^{2'''B}$), 21.2 (s, $C^{4'''B}$), 21.7 (s, $C^{6'''A}$), 29.2 (s, $C^{5'''}$), 29.5 (s, $C^{3'''}$), 29.6 (s, $C^{7'''}$), 30.1 (s, $C^{1'''}$), 99.3 (d, $^{1}J_{CRh}$ = 6.5 Hz, *Cp**-Me), 117.7 (s, Me*CN*), 121.3 (s), 124.4 (s, $C^{5'}$), 125.0 (s, $C^{4''}$), 128.3 (s, $C^{6'}$), 130.7 (s), 130.9 (s), 131.9 (s), 133.8 (s), 134.0 (s), 140.3 (s), 140.6 (s), 142.3 (s), 153.3 (s, $C^{5''}$), 165.4 (s, $C^{1''}$), 173.5 (d, $C^{3'}$), 223.5 (s, CO), 226.9 (s, CO) ppm. ^{31}P{^{1}H} NMR (161.97 MHz, CD_3CN): δ = −144.6 (hept, $^{1}J_{PF}$ = 706 Hz, PF_6), 71.9 (dd, $^{3}J_{PP}$ = 23.6 Hz, $^{3}J_{PP}$ = 6.0 Hz, $P(^{i}Pr)_2$), 72.3 (dd, J_{PP} = 23.5 Hz, $^{3}J_{PP}$ = 5.5 Hz, $P(^{i}Pr)_2$), 80.5 (t, $^{3}J_{PP}$ = 5.9 Hz, $P(aryl)_3$) ppm. ^{19}F{^{1}H} NMR

(376.53 MHz, CD_3CN): δ = −73.0 (d, $^{1}J_{FP}$ = 706 Hz, PF_6) ppm. IR (KBr), selected vibrations: 1929 (s, Mo-CO), 1842 (s, Mo-CO), 842 (s, PF_6), cm^{-1}. HR-MS: ($C_{48}H_{59}MoNO_3P_3Rh$; $[M-PF_6]^{+}$, pos. ESI) calcd.: 990.173, found: 990.179.

2.8. Synthesis of **C1**

4 (111 mg, 0.10 mmol, 1.0 equiv.) and $NaPF_6$ (17 mg, 0.10 mmol, 1.0 equiv.) were dissolved in MeCN (2 ml) and stirred under reflux over night. The mixture was filtered over celite and the solvent was removed under reduced pressure. The residue was dissolved in THF (2 ml), precipitated with *n*-pentane (2 ml) and filtered off. The yellow powder was washed with *n*-pentane (3 × 1 ml) and dried under vacuum. The product was obtained as a yellow powder (122 mg, 0.09 mmol, 97%). Single crystals were obtained by diffusion of *n*-hexane into a saturated THF solution, over the course of 4 months. In the obtained crystals, MeCN was displaced by N_2.

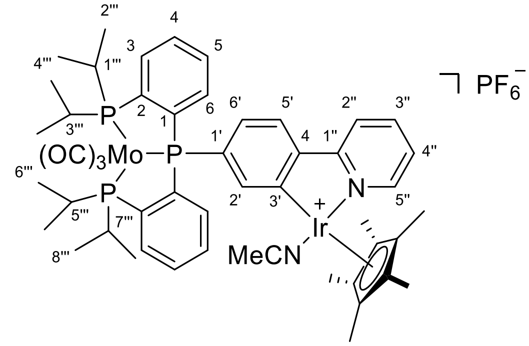

^{1}H NMR (400.1 MHz, CD_3CN): δ = 0.62 (dd, $^{3}J_{HP}$ = 12.9 8Hz, $^{3}J_{HH}$ = 7.0 Hz, 3 H, 2’’’-H^{A}), 0.76 (dd, $^{3}J_{HP}$ = 12.9 Hz, $^{3}J_{HH}$ = 7.0 Hz, 3 H, 4’’’-H^{A}), 0.97 – 1.13 (m, 12 H, 2’’’, 4’’’, 6’’’, 8’’’-H^{B}), 1.24 (dd, $^{3}J_{HP}$ = 16.8 Hz, $^{3}J_{HH}$ = 6.7 Hz, 3 H, 6’’’-H^{A}), 1.34 (dd, $^{3}J_{HP}$ = 17.0 Hz, $^{3}J_{HH}$ = 6.8 Hz, 3 H, 8’’’-H^{A}), 1.51 (s, 15 H, Cp*), 1.96 (s, 3 H, MeCN), 2.06 (m_c, 1 H, 1’’’-H), 2.17 (m_c, 1 H, 3’’’-H), 2.79 (m_c, 1 H, 5’’’-H), 2.90 (m_c, 1 H, 7’’’-H), 6.96 (ddd, $^{3}J_{HH}$ = 9.9 Hz, $^{3}J_{HH}$ = 8.0 Hz, $^{3}J_{HH}$ = 1.7 Hz, 1 H, 6’-H), 7.35 (ddd, $^{3}J_{HH}$ = 7.3 Hz, $^{3}J_{HH}$ = 5.7 Hz, $^{3}J_{HH}$ = 1.5 Hz, 1 H, 4’’-H), 7.51 – 7.56 (m, 5 H), 7.59 (dd, $^{3}J_{HH}$ = 10.9 Hz, $^{4}J_{HH}$ = 1.5 Hz, 1 H, 2’-H), 7.74 (dd, $^{3}J_{HH}$ = 8.1 Hz, $^{4}J_{HH}$ = 2.1 Hz, 1 H, 5’-H), 7.82 – 7.85 (m, 2 H), 7.94 (ddd, $^{3}J_{HH}$ = 8.2 Hz, $^{4}J_{HH}$ = 7.3 Hz, $^{5}J_{HH}$ = 1.6 Hz 1 H), 7.98 – 8.04 (m, 2 H), 8.72 (ddd, $^{3}J_{HH}$ = 5.6 Hz, $^{4}J_{HH}$ = 1.3 Hz, $^{5}J_{HH}$ = 0.8 Hz 1 H, 5’’-H) ppm. ^{13}C{^{1}H} NMR (100.6 MHz, CD_3CN): δ = 1.32 (sept, *Me*CN), 9.0 (s, Cp*-*Me*), 19.6 (s, C$^{2’’’A}$), 20.0 (s, C$^{4’’’A}$), 20.5 (s, C$^{6’’’B}$), 20.6 (s, C$^{8’’’B}$), 21.37 (s, C$^{8’’’A}$) 21.39 (s, C$^{2’’’B}$), 21.6 (s, C$^{4’’’B}$), 21.7 (s, C$^{6’’’A}$), 29.1 (m, C$^{5’’’}$), 29.2 (m, C$^{3’’’}$), 29.3 (s, C$^{7’’’}$), 29.9 (s, C$^{1’’’}$), 92.2 (s,

*Cp**-Me), 118.3 (s, Me*CN*), 121.1 (s), 124.6 (d, J_{CP} = 9.7 Hz, $C^{5'}$), 125.8 (s, $C^{4''}$), 127.6 (d, J_{CP} = 12.1 Hz $C^{6'}$), 130.7 (s), 130.9 (s), 131.0 (d, J_{CP} = 5.1 Hz), 131.8 (d, J_{CP} = 3.1 Hz), 131.9 (d, J_{CP} = 3.7 Hz), 133.7 (d, J_{CP} = 11.3 Hz), 133.9 (d, J_{CP} = 11.9 Hz), 140.0 (s), 140.1 (s), 140.5 (s), 146.5 (d, J_{CP} = 1.2 Hz) 153.8 (s, $C^{5''}$), 167.3 (s, $C^{1''}$) ppm. $^{31}P\{^1H\}$ NMR (161.97 MHz, CD_3CN): δ = −144.7 (hept, $^1J_{PF}$ = 706 Hz, PF_6), 72.1 (dd, $^3J_{PP}$ = 23.4 Hz, $^3J_{PP}$ = 6.2 Hz, $P(^iPr)_2$), 72.3 (dd, J_{PP} = 23.5 Hz, $^3J_{PP}$ = 6.1 Hz, $P(^iPr)_2$), 80.2 (t, $^3J_{PP}$ = 5.9 Hz, $P(aryl)_3$) ppm. $^{19}F\{^1H\}$ NMR (376.53 MHz, CD_3CN): δ = −73.0 (d, $^1J_{FP}$ = 706 Hz, PF_6) ppm. IR (KBr), selected vibrations: 1929 (s, Mo-CO), 1842 (s, Mo-CO), 844 (s, PF_6) cm^{-1}. HR-MS: ($C_{48}H_{59}IrMoNO_3P_3$; $[M-PF_6]^+$, pos. ESI) calcd.: 1080.234, found: 1080.231.

3. Catalysis

3.1. Procedure 1

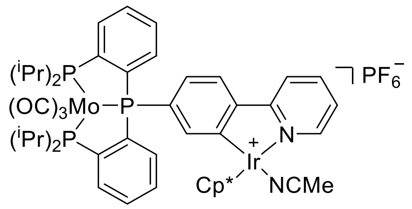

$C_{50}H_{61}F_6IrMoN_2O_3P_4$

C1

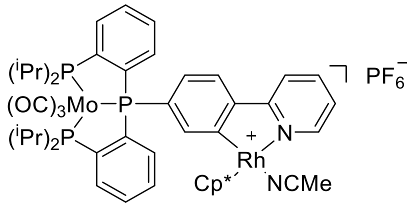

$C_{50}H_{61}F_6MoN_2O_3P_4Rh$

C2

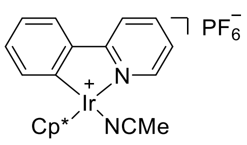

$C_{23}H_{26}F_6IrN_2P$

M1

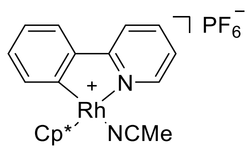

$C_{23}H_{26}F_6N_2PRh$

M2

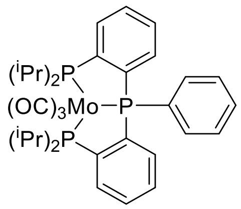

$C_{33}H_{41}MoO_3P_3$

M3

A 50 ml autoclave was heated to 50 °C at 1×10^{-3} mbar for 2 h and then charged with an Ar atmosphere, using a special made adapter for connecting to a Schlenk line. The *catalyst* (1 ml of a 3 mM MeCN sol., corresponds to 3 µmol of catalyst) was added. When using a different solvent then acetonitrile, the solvent was removed under vacuum. *Base* (0.5 mmol) and *solvent* (to achieve a total volume of 2 ml) were added. The adapter was exchanged for the head of the autoclave and the apparatus was charged with 30 bar of dihydrogen. The pressure was vented down to 10 bar, raised back up to 30 bar and vented again, to flush out any air that was introduced while changing the top. After repeating this procedure for 3 times the dihydrogen pressure was set to 30 bar and the mixture was stirred at 50 °C for 30 min. Then 27 bar of CO_2 were added (57 bar of total pressure) and the mixture was stirred at 50 °C for 22 h. The reaction was stopped by venting the reaction vessel. The mixture was dissolved in acetone (3 × 2 ml) and the solvent removed under reduced pressure. The residue was dissolved in D_2O (1 ml) and filtered over a glass filter paper. The concentration of formate was determined by ^1H NMR spectroscopy using a capillary containing benzene in C_6D_6, which had been calibrated using formate solutions with known concentrations in D_2O. The yield was calculated accordingly.

3.2. Catalysts

$$CO_2 + H_2 \xrightarrow[\text{MeCN, 50 °C, 22 h}]{\text{DBU, 0.6 mol \% } \textbf{Cat}} [DBU(H)]HCO_2$$

All reactions were performed according to **Procedure 1** using 1,8-diazabicyclo[5.4.0]undec-7-ene (DBU) (75 µl, 0.5 mmol, 1.0 equiv.), *catalyst* (1.0 ml of a 3 mM MeCN sol., corresponds to 3 µmol of catalyst) and MeCN (1 ml, corresponding to a total volume of 2 ml). When using a mixture of two catalysts no additional MeCN was added.

Table S1: Performance of different catalyst for CO_2 hydrogenation.

entry	catalyst	n([DBU(H)]HCO₂) [µmol]	yield [%][a]	TON
1	**C1**	383	77	128
		394	79	131
		377	75	126
2	**M3**	5	1	2
		2	0	1
3	**M1**	70	14	23
		75	15	25
4	**M1** + **M3**	110	22	37
		111	22	37
5	**No cat.**	0	0	0
6	**C2**	34	7	11
		50	10	17
7	**M2**	10	2	3
		15	3	5
8	**M2** + **M3**	8	2	3
		10	2	3
12	**C1**[b]	36	7	12
13	**C1**[c]	168	34	56

[a] In relation to 0.5 mmol DBU. [b] $p(H_2) = p(CO) = 15$ bar, corresponds to a total pressure of 30 bar. [c] reaction mixture was not stirred.

3.3. Solvent

$$CO_2 + H_2 \xrightarrow[\text{solvent, 50 °C, 22 h}]{\text{DBU, 0.6 mol \% } \mathbf{C2}} [DBU(H)]HCO_2$$

All reactions were performed according to **Procedure 1** using DBU (75 µl, 0.5 mmol, 1.0 equiv.) and **C2** (1.0 ml of a 3 mM MeCN sol., corresponds to 3 µmol of catalyst). After removal of the solvent under vacuum *solvent* (2 ml) was added. When using deuterated solvent, the reaction mixture was taken directly from the reaction vessel, filtered over a glass filter paper and the yield determined using ^1H NMR spectroscopy.

Table S2: Influence of solvent on catalytic CO_2 hydrogenation using **C2**.

entry	Solvent	n([DBU(H)]HCO₂) [µmol]	yield [%][a)]	TON
1	MeCN	383	77	128
		394	79	131
		377	75	126
2	DMSO-d_6	36	7	12
3	D_2O	48	10	16
4	THF	46	9	15

a) In relation to 0.5 mmol DBU.

3.4. Base

$$CO_2 + H_2 \xrightarrow[\text{MeCN, 50 °C, 22 h}]{\text{Base, 0.6 mol \% } \mathbf{C2}} [DBU(H)]HCO_2$$

All reactions were performed according to **Procedure 1** using *base* (0.5 mmol, 1.0 equiv.), **C2** (1.0 ml of a 3 mM MeCN sol., corresponds to 3 µmol of catalyst) and MeCN (1 ml, corresponding to a total volume of 2 ml).

Table S3: Influence of base on catalytic CO_2 hydrogenation using **C2**.

entry	Base	n([DBU(H)]HCO_2) [µmol]	yield [%][a)]	TON
1	Li_2CO_3	traces	0	0
2	LiOH	5	1	2
3	Cs_2CO_3	13	3	4
4	No base	Traces	<1	0
5	NEt_3	135	27	45
6	DIPEA	92	18	31
7	$PhNP(NMe_2)_3$	6	1	2
8	DBU	383<br>394<br>377	77<br>79<br>75	128<br>131<br>126
9	TMG	500	100	168

a) In relation to 0.5 mmol base.

3.5. Reaction time / Determination of TOF

$$CO_2 + H_2 \xrightarrow[\text{MeCN, 50 °C, time}]{\text{TMG, 0.5 mol \% } \mathbf{C2}} [TMG(H)]HCO_2$$

All reactions were performed according to **Procedure 1** using Tetramethylguanidine (TMG) (67 µl, 0.634 mmol, 1.0 equiv.) and **C2** (1.0 ml of a 3 mM MeCN sol., corresponds to 3 µmol of catalyst).

Table S4: Monitoring of **C2** for CO_2 hydrogenation using TMG as a base.

entry	Time [h]	n([TMG(H)]HCO2) [µmol]	yield [%][a)]	TON
1	2	31	5	10
		41	6	14
2	4	45	7	15
		84	13	28
3	8	98	15	33
		113	18	38
4	16	277	44	92
		216	34	72
5	22	550	87	183
		505	79	168
6	70	1265	200	422
		1122	177	374
7	140	1109	175	370

[a)] In relation to 0.634 mmol TMG.

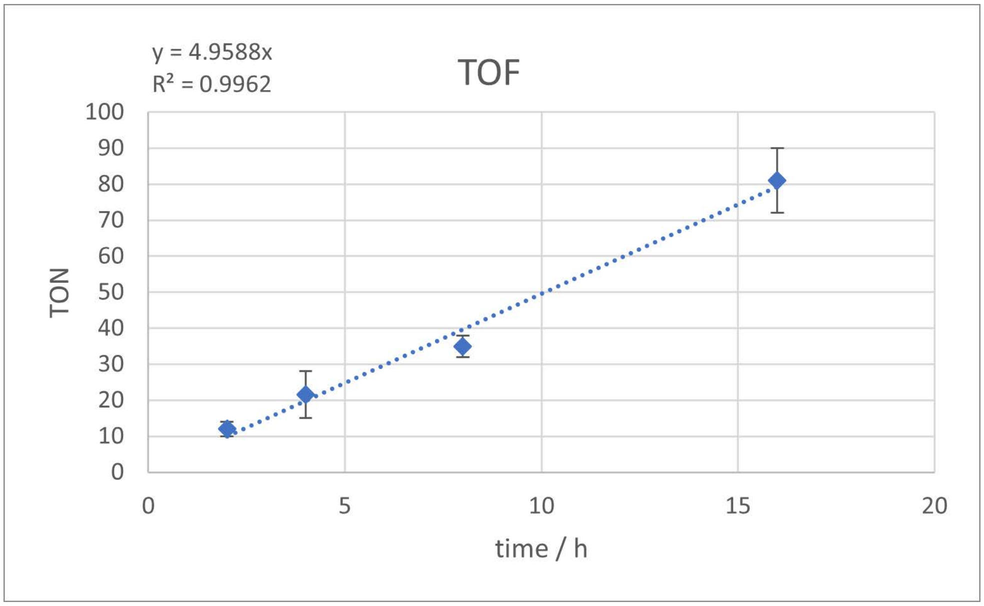

Scheme 1: Kinetic plot of formate turnovers versus time (hours) using entries 1 – 4 in Table S4. The plot is linear and the slope represents the rate ($TOF_I = 5.0\ h^{-1}$).

4. Determination of Thermodynamic Hydricity of [Ir^{III}H] [6]

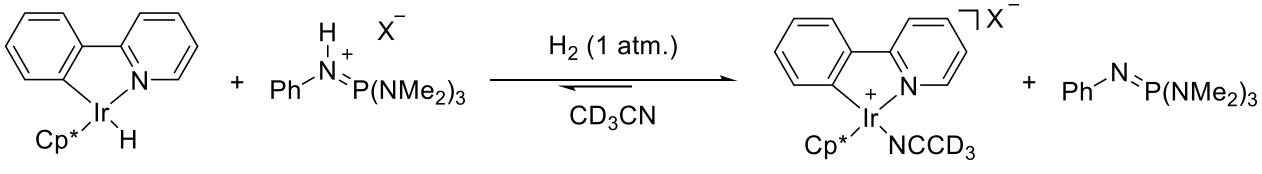

M1 (8.2 mg, 12.3 µmol, 1.0 equiv.), tris(dimethylamino)(phenylamino)phosphonium tetrafluoroborate (13.0 mg, 51.1 µmol, ~4 equiv.) and tris(dimethylamino)(phenylimino)phosphorane (15.4 mg, 45.0 µmol) were accurately weighed into a YOUNG NMR tube and dissolved in CD_3CN (0.7 ml). The mixture was degassed using freeze-pump-thaw and set under 1 atm of H_2. The reaction was monitored using ^{1}H NMR spectroscopy until the equilibrium was reached (24 h). The ratio of **[Ir^{III}H]** and **[Ir^{III}(NCMe)]X** was determined using the respective Cp* signal. The concentrations of the four components were calculated from their initial concentration. The procedure was repeated twice. An average equilibrium constant (K_{eq}) of 0.75 atm was calculated. By substituting this value in eq. 1, a ΔG^0_{H-} of 47.1(7) $^{kcal}/_{mol}$ was obtained. [6]

$$\Delta G^0_{H^-}(kcal/mol) = -1.364\log(K_{eq}) - 1.364pK_a(Et_3NH^+) + 76.0 \qquad (1)$$

5. Mechanistic ^{1}H NMR Spectroscopic Studies

5.1. Coordination of TMG to **M1**

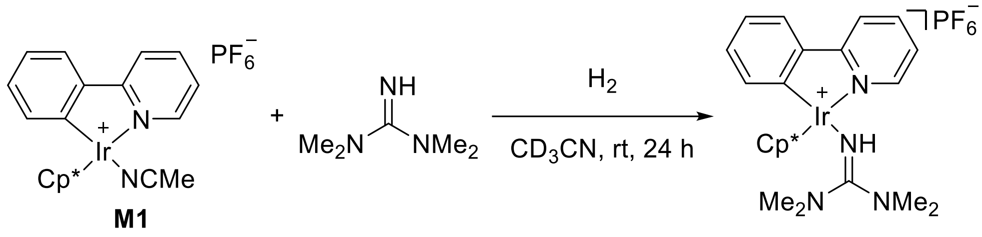

M1 (5.4 mg, 8.0 µmol, 1.0 equiv.) and 1,1,3,3-tetramethylguanidine (6.1 mg, 53 µmol, ~10 equiv.) were accurately weighed into a YOUNG NMR tube and dissolved in CD_3CN (0.7 ml). The mixture was degassed using freeze-pump-thaw and set under 1 atm of H_2. The reaction was monitored for 24 h using ^{1}H NMR spectroscopy.

^{1}H NMR (400 MHz, CD_3CN)

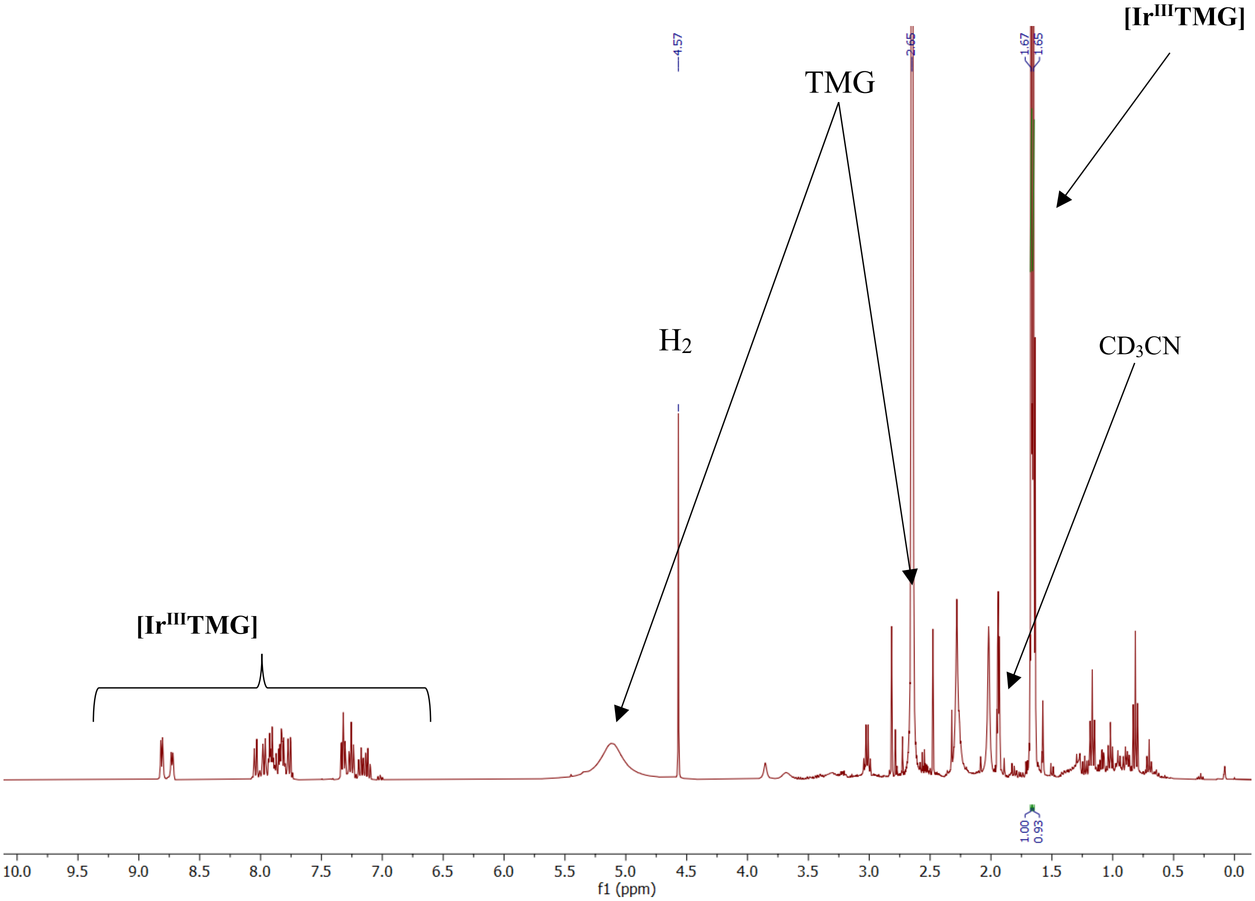

5.2. **M1** under H_2 atmosphere

While **[Ir^{III}H]** is stable in CD_3CN for weeks, **M1** is decomposing slowly over the course of several days under 1 atm of H_2. A signal in the ^{1}H NMR spectrum at −15.5 ppm emerges. Whereas this single signal is almost identical in chemical shift to the hydridic signal of **[Ir^{III}H]**, the remaining spectrum does not fit, particularly the Cp* resonance. Additionally, no Cp* signal with a fitting integral of 15 in relation to the signal at −15.5 ppm was found. To find out whether this signal corresponds to the proposed intermediate **[Ir^{III}(η^2-H$_2$)]$^+$**, an HD test was performed and the T1-relaxation time of the hydridic signal was measured [16–19]. When using HD instead of H_2, no change in the ^{1}H NMR spectrum could be observed. The T1-relaxation time was determined using an inversion recovery experiment (at 400 MHz, at 298 K) as 5306 ms, which is too high for both a non-classical hydride (>80 ms) and a classical hydride (<150 ms). Like the decomposition of **M1** under H_2 atmosphere in THF-d_8 this signal could arise from a multimetallic cluster due to degradation of the active catalyst. Similar Rh nanoclusters were reported to have T1 times between 938 and 1732 ms (at 300 K and 500 MHz), which is still significantly shorter than those found here, but also significantly larger than those typical for both classical and non-classical hydrides. Furthermore, when the atmosphere was subsequently changed from H_2 to N_2 the observed signal did not subside, which means that the species is not in equilibrium with free solvated dihydrogen, but rather results from a reaction of the complex with dihydrogen. However, as the resulting product could not be isolated, full characterization was not possible.

Experimental Procedure

M1 (6.4 mg, 9.6 µmol, 1.0 equiv.) was weighed into a YOUNG NMR tube and dissolved in CD_3CN (0.5 ml). The mixture was degassed using freeze-pump-thaw and set under 1 atm of H_2. When HD gas instead of H_2 gas was used, no change in the ^{1}H NMR spectrum was observed. The decomposition was monitored for 1 week using ^{1}H NMR spectroscopy. Then the T1 time of the signal at −15.5 ppm was determined (T1 = 5306 ms at 25 °C and 400 MHz). After T1 was measured, the mixture was again degassed using freeze-pump-thaw and set under N_2 atmosphere, which led to no further changes.

^{1}H NMR (400 MHz, CD_3CN)

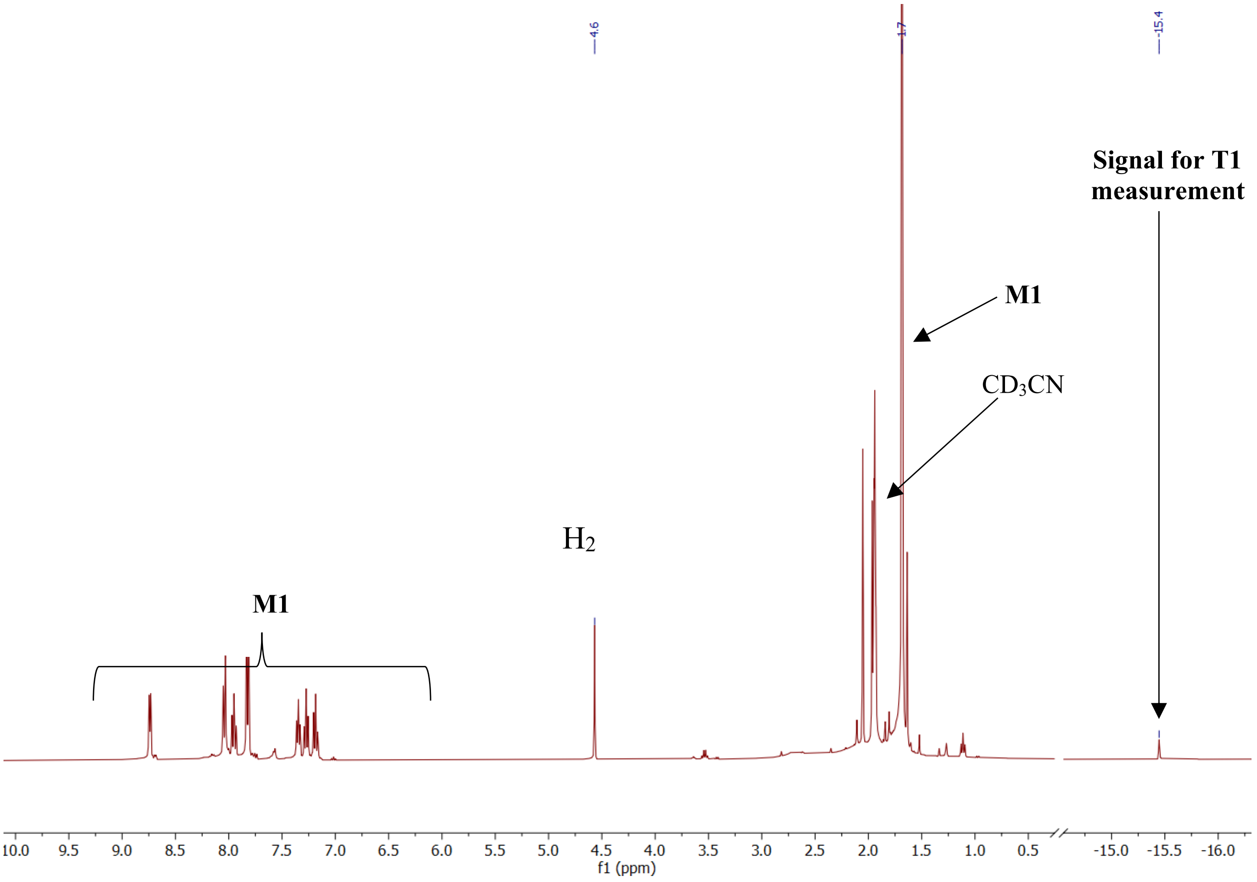

5.3. Coordination of HCO_2^- to **M1** and subsequent H_2 activation

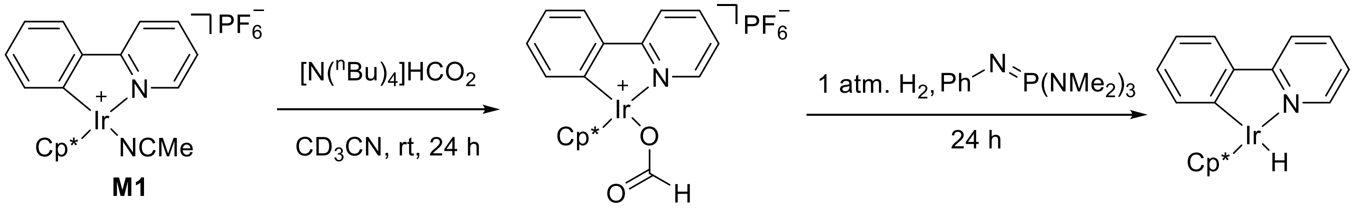

M1 (2.9 mg, 4.3 µmol, 1.0 equiv.) and tetrabutylammonium formate (1.3 mg, 4.3 µmol, 1.0 equiv.) were accurately weighed into a YOUNG NMR tube and dissolved in CD_3CN (0.7 ml). The mixture was kept at room temperature for 24 h, then a ^1H NMR spectrum was recorded. Phenyltris(dimethylamino)phosphorane (3.3 mg, 12.9 µmol, 3.0 equiv.) was added, the mixture degassed using freeze-pump-thaw and set under 1 atm of H_2. The reaction was monitored for 24 h using ^1H NMR spectroscopy.

^1H NMR (400 MHz, CD_3CN) before H_2 activation:

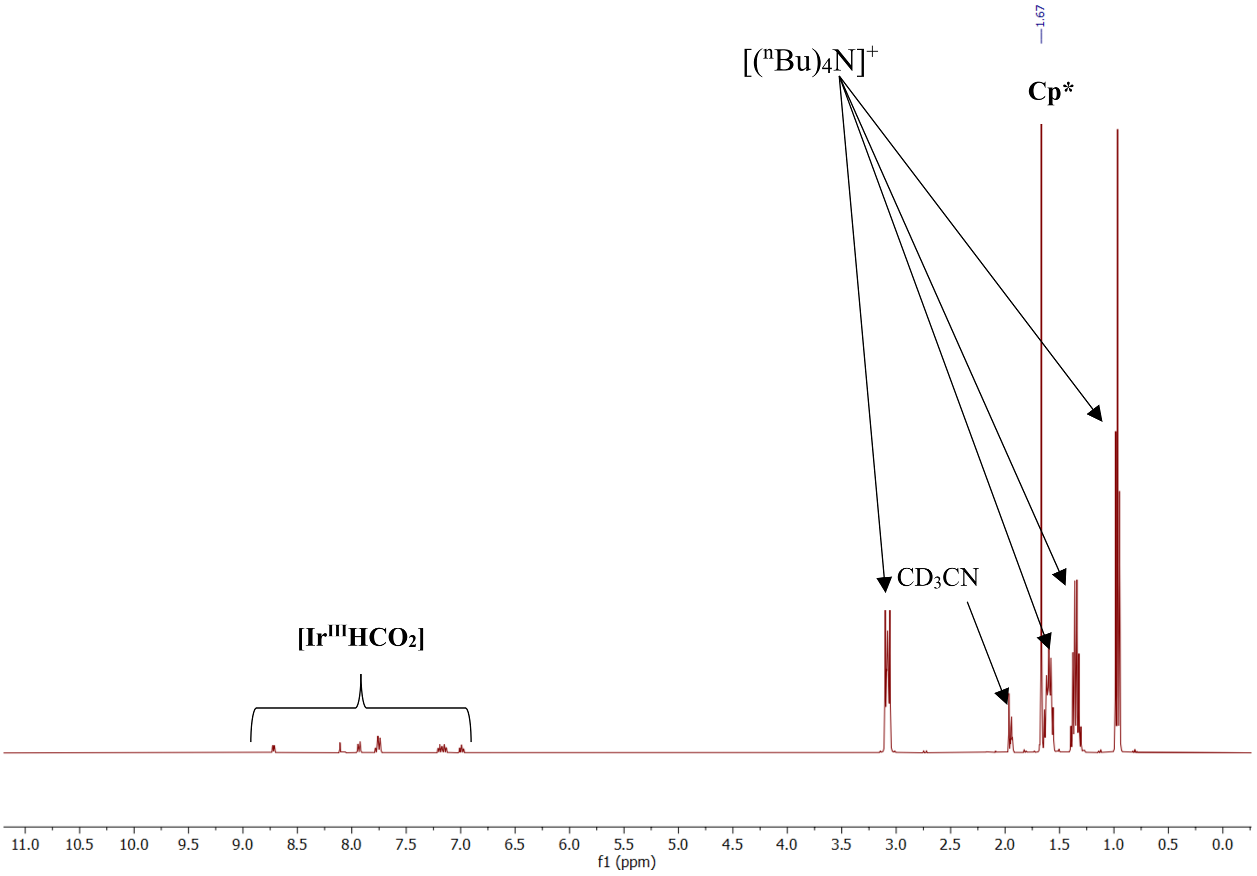

^{1}H NMR (400 MHz, CD_3CN) after H_2 activation:

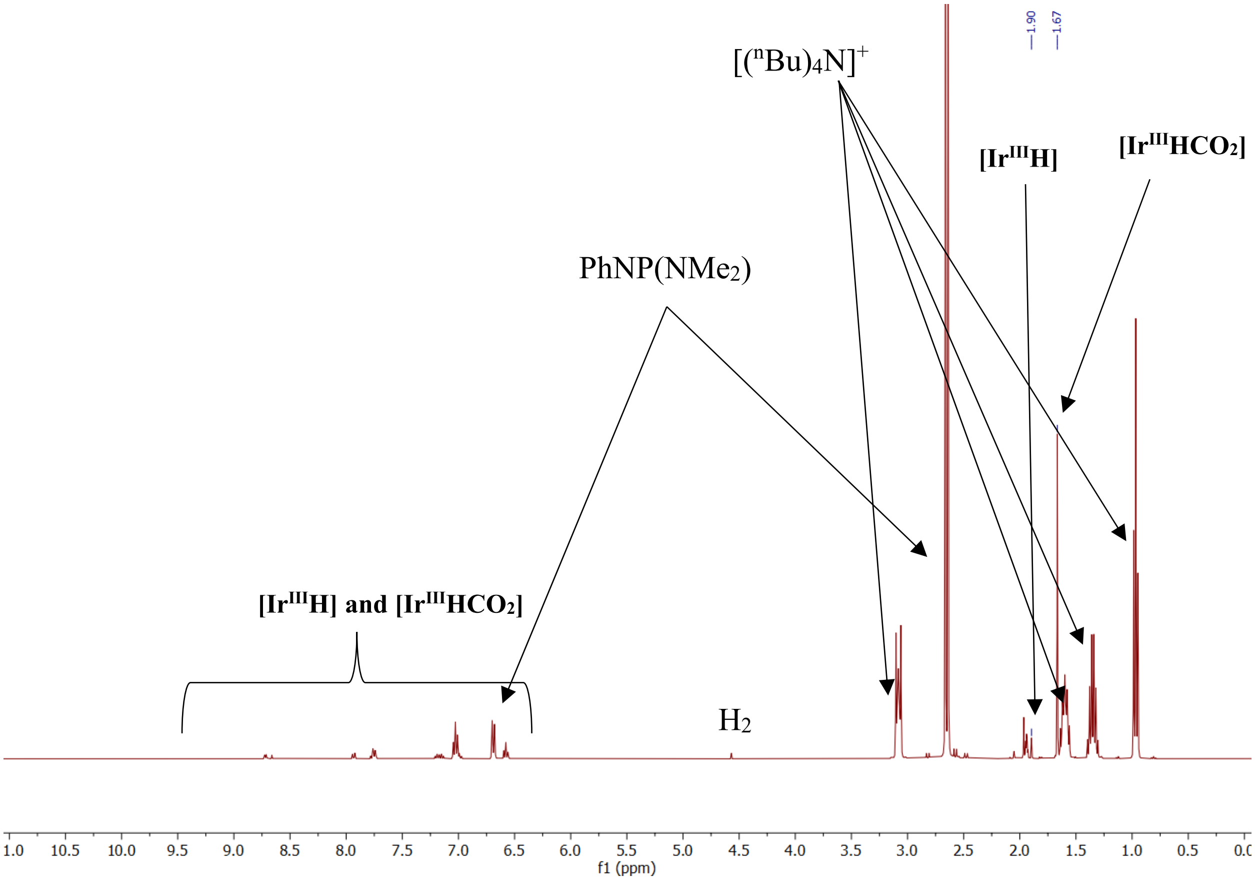

5.4. H_2 activation by a 1:1 mixture of **M1** and **M3**

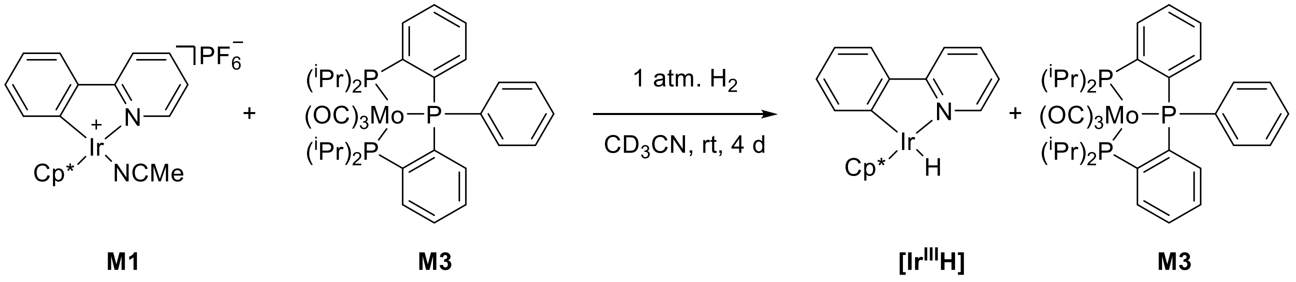

M1 (8.0 mg, 5.4 µmol, 1.0 equiv.), **M3** (8.0 mg, 5.3 µmol, 1.0 equiv.) and NEt_3 (2.4 mg, 24 µmol, 3.0 equiv.) were dissolved in CD_3CN (0.5 ml) in a Young NMR tube. The mixture was degassed using freeze-pump-thaw and set under 1 atm of H_2. Every day for 4 days a ^1H NMR spectrum and a $^{31}P\{^1H\}$ NMR spectrum were recorded.

^1H NMR (400 MHz, CD_3CN) after 4 days:

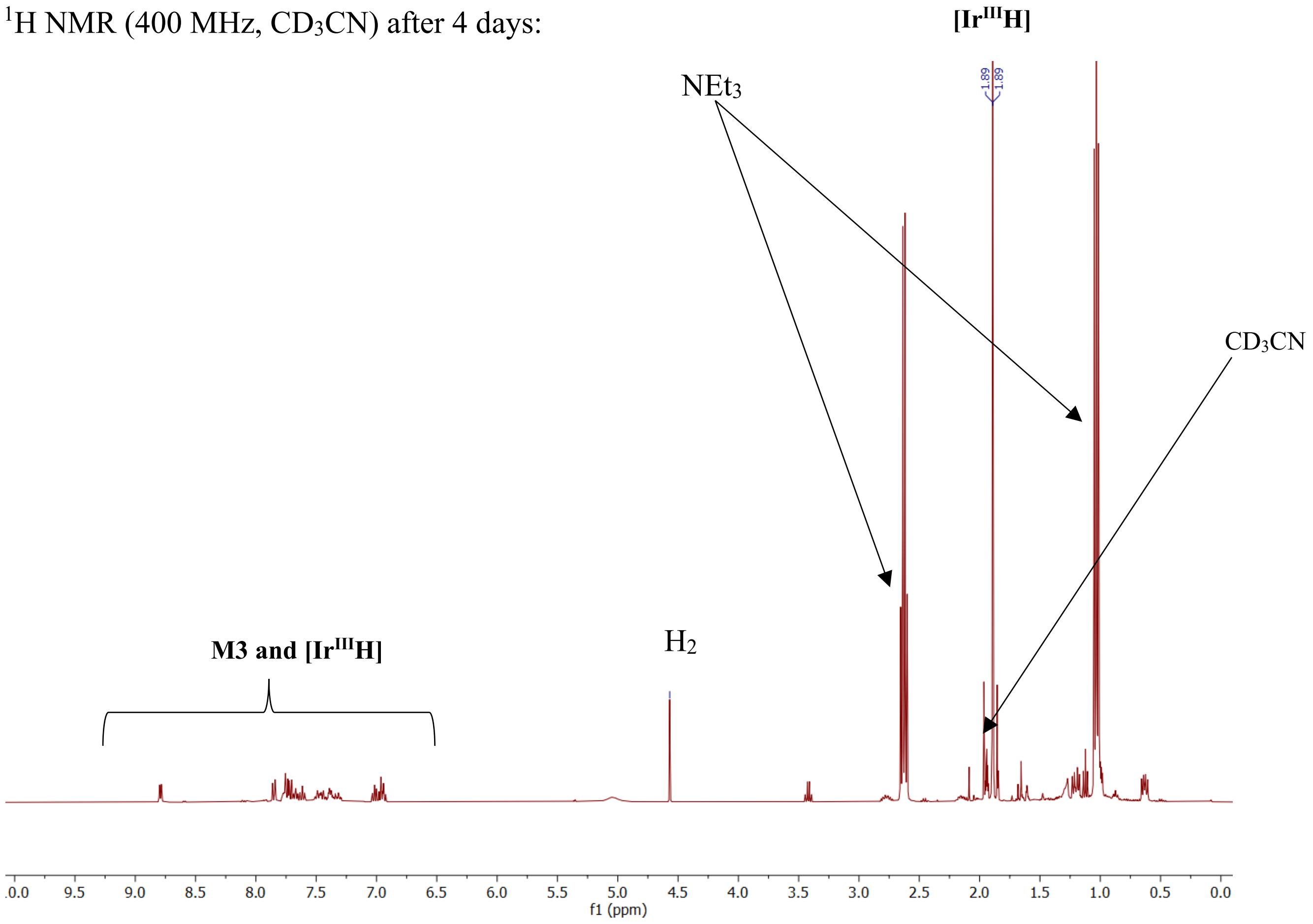

$^{31}P\{^{1}H\}$ NMR (162 MHz, CD_3CN) after 4 days:

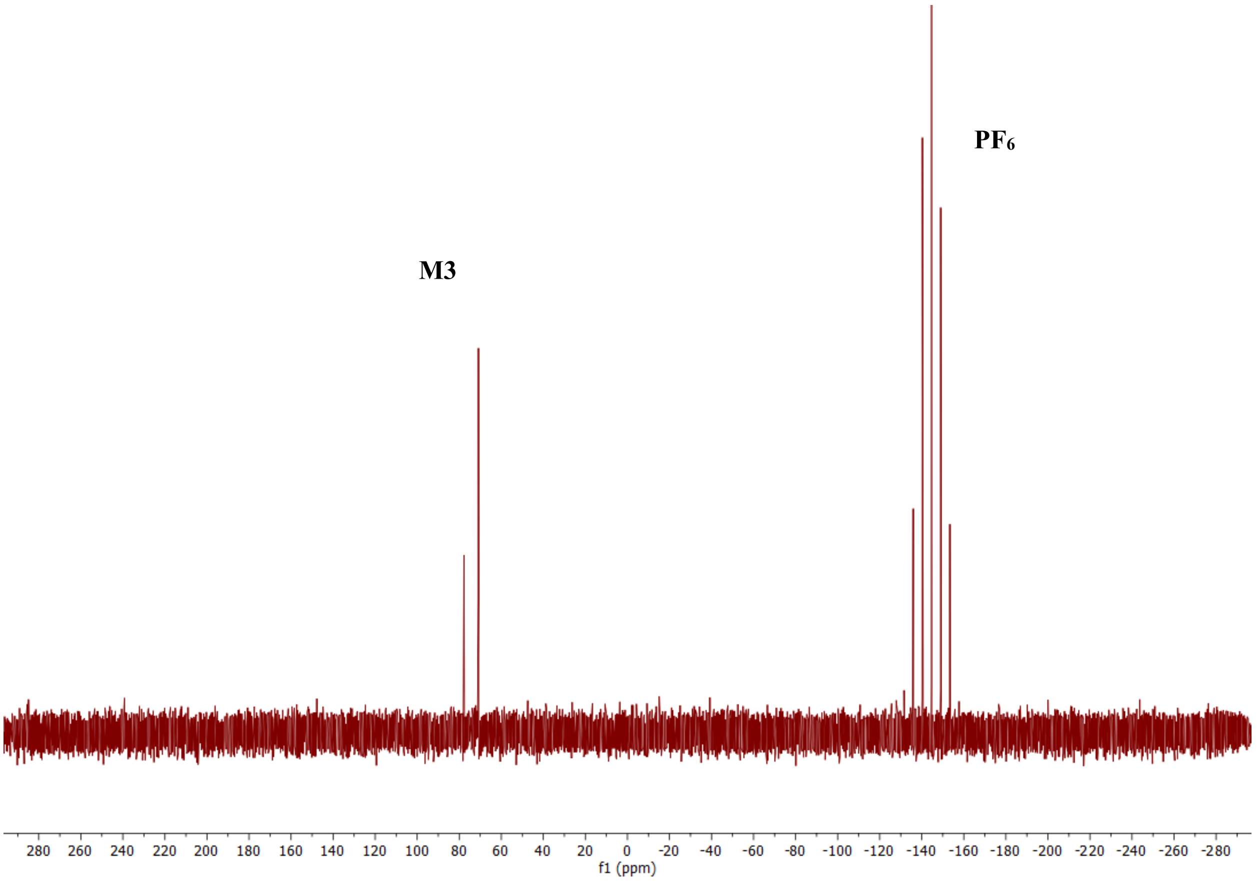

6. ^1H, $^{31}P\{^1H\}$, $^{13}C\{^1H\}$ and $^{19}F\{^1H\}$ NMR spectra

6.1. **1**

^1H NMR (400 MHz, CD_2Cl_2)

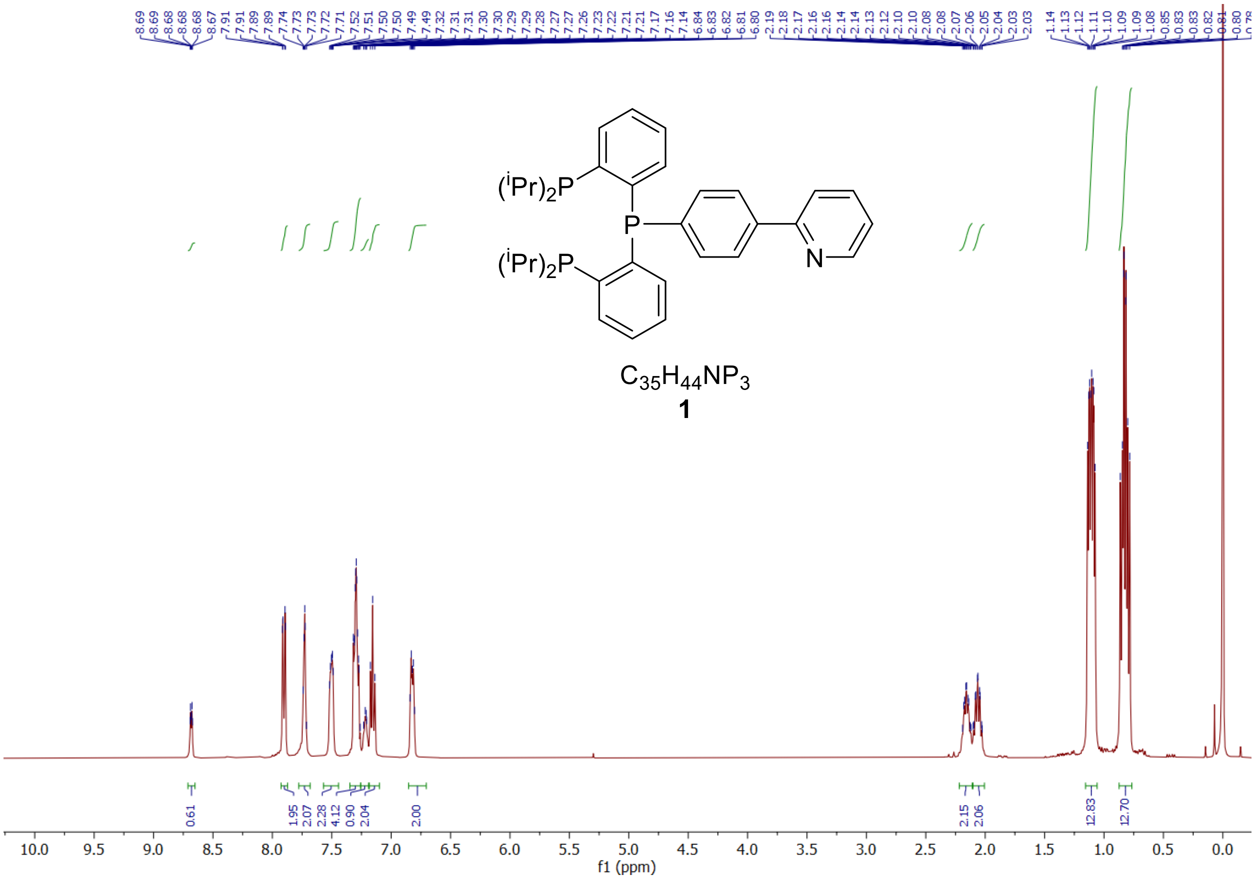

$^{31}P\{^{1}H\}$ NMR (162 MHz, CD_2Cl_2)

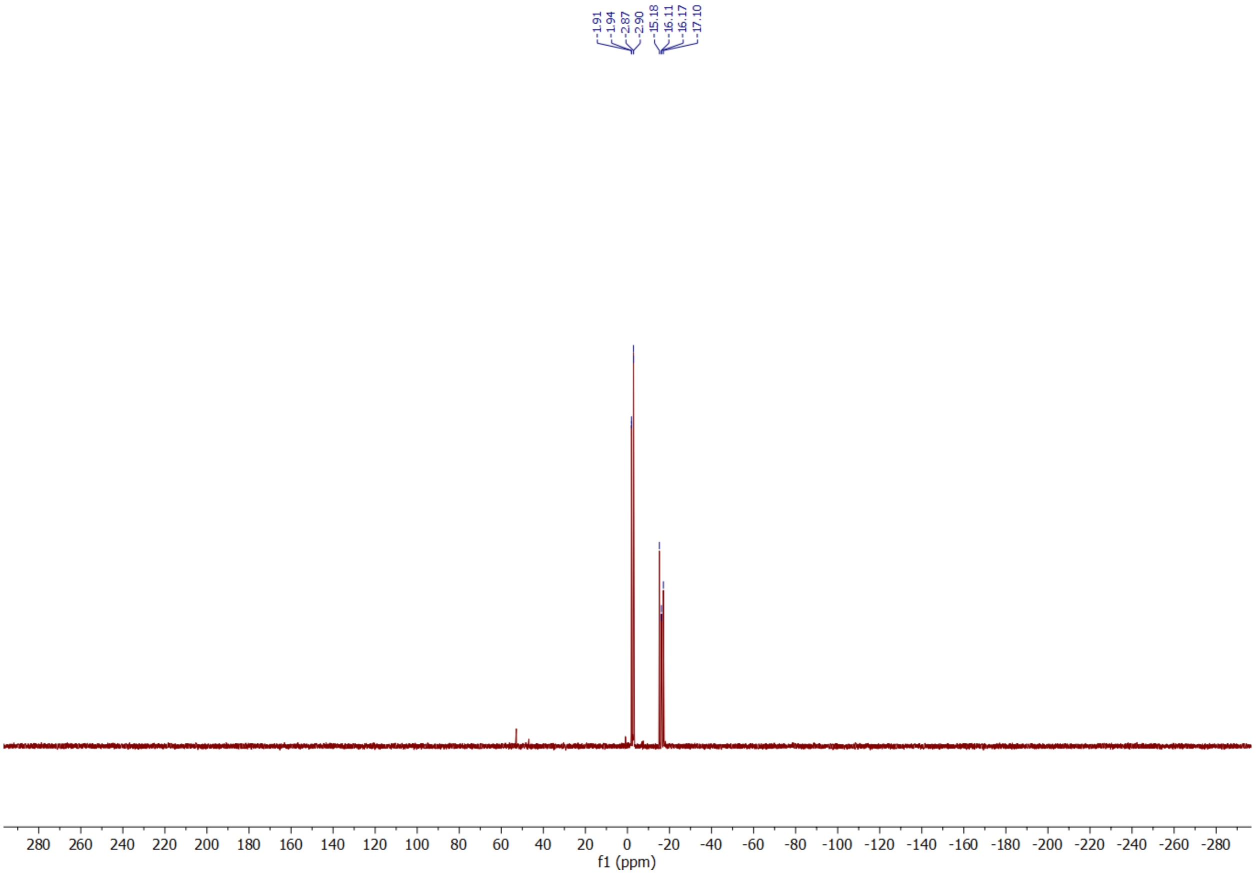

$^{13}C\{^{1}H\}$ NMR (100 MHz, CD_2Cl_2)

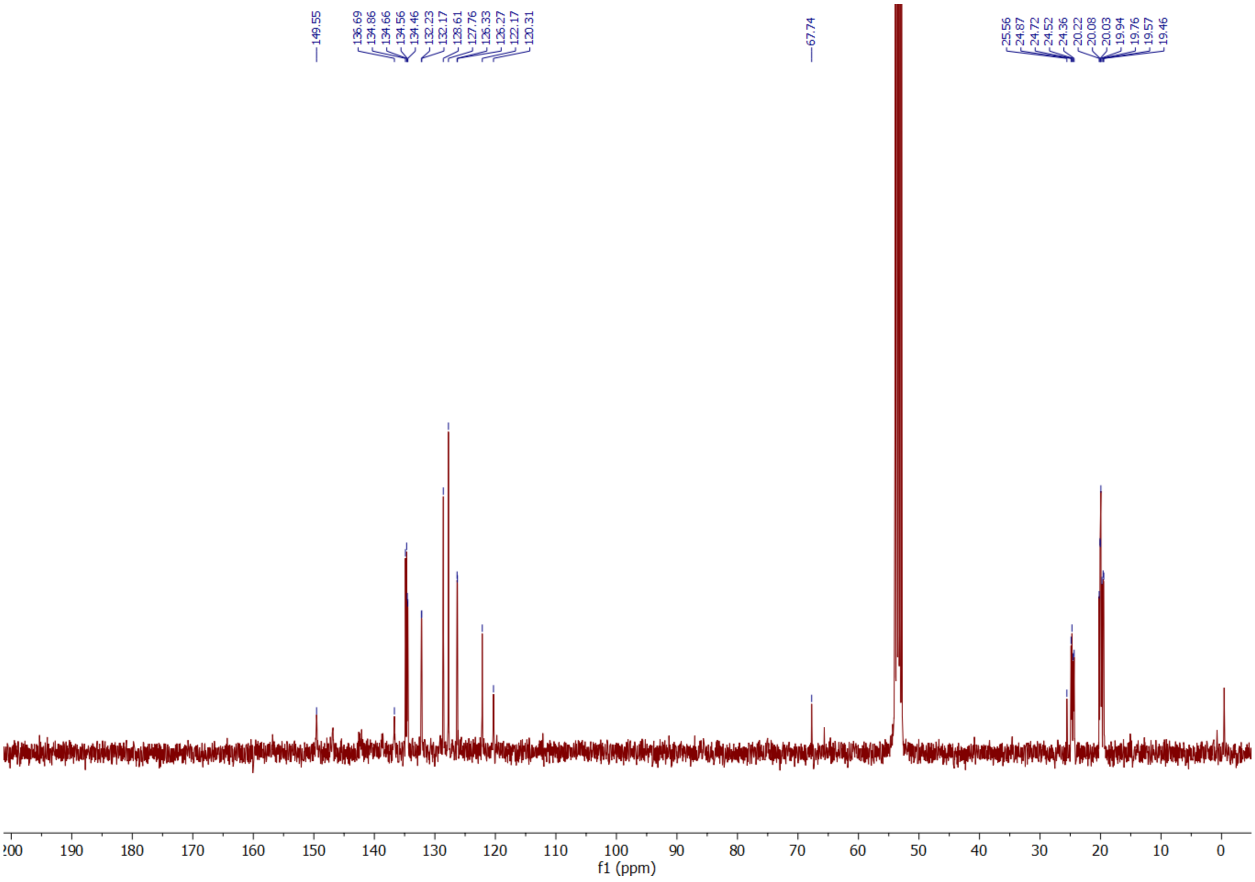

6.3. **M3**

^{1}H NMR (400 MHz, CD_2Cl_2)

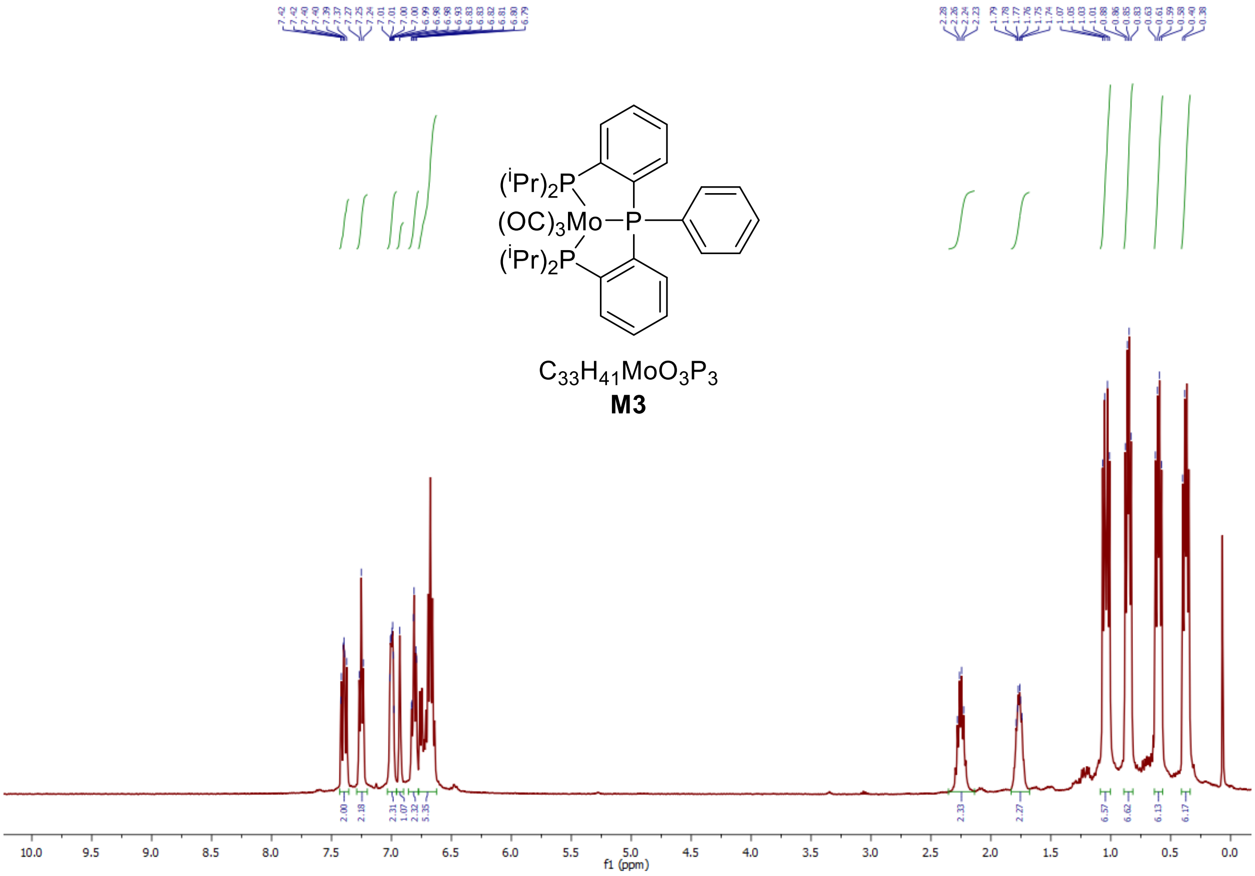

$^{31}P\{^1H\}$ NMR (162 MHz, CD_2Cl_2)

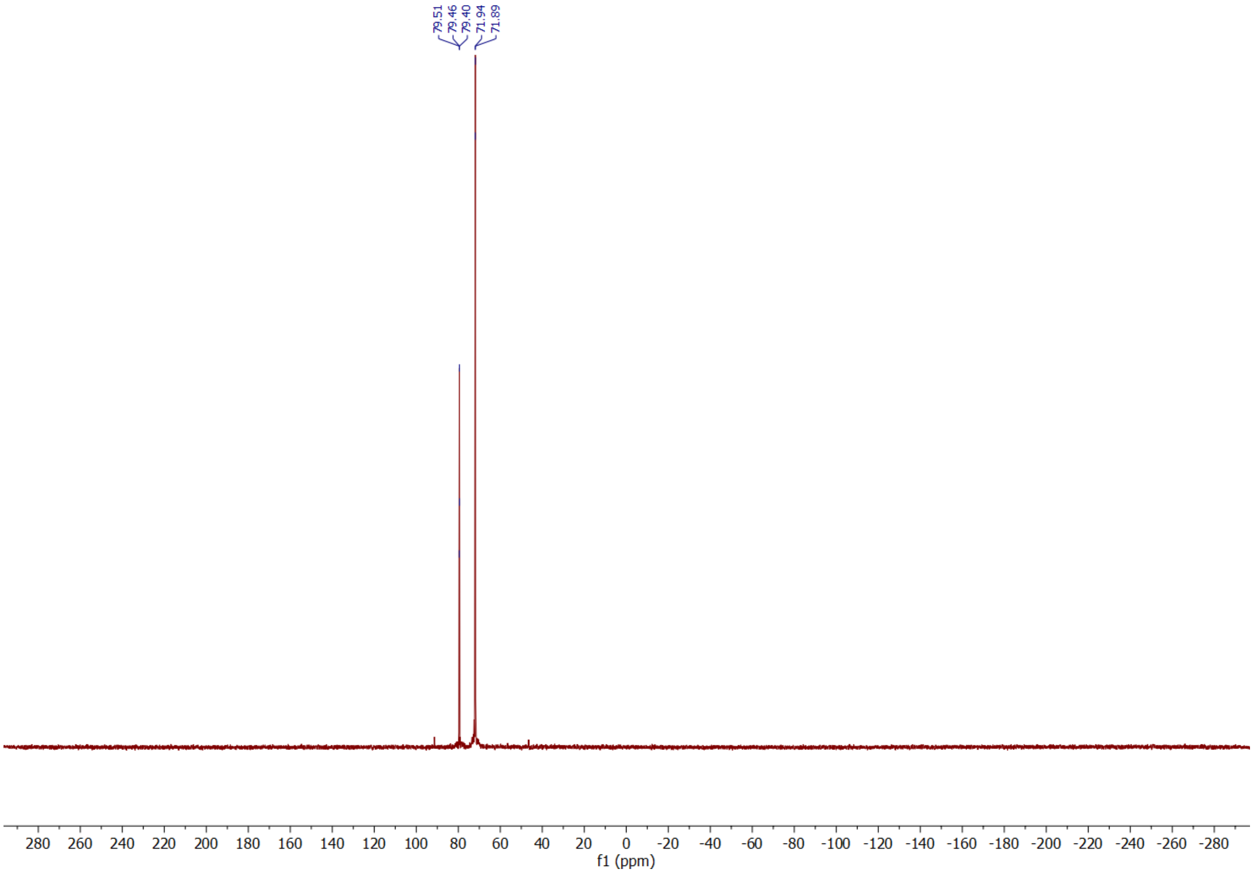

$^{13}C\{^1H\}$ NMR (100 MHz, CD_2Cl_2)

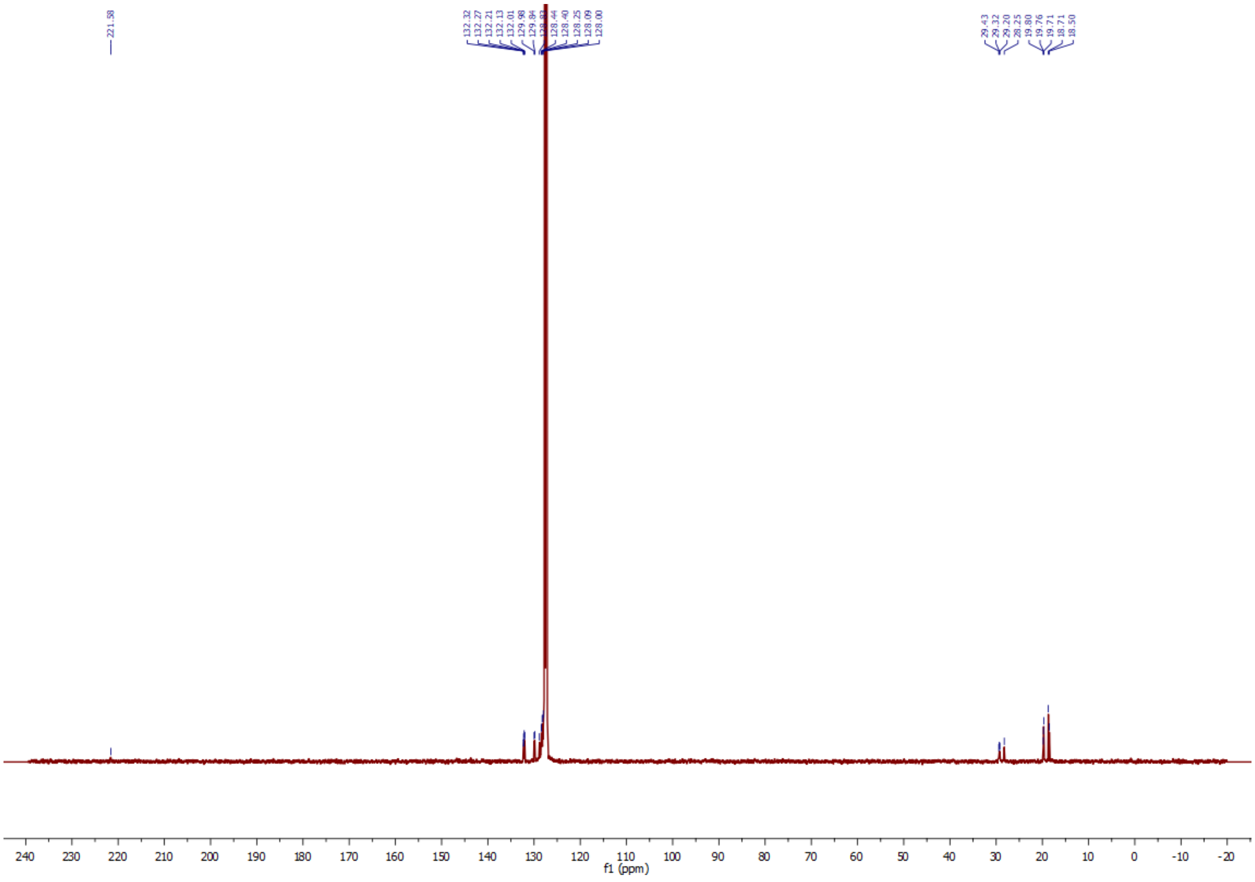

6.4. **2**

^{1}H NMR (400 MHz, C_6D_6)

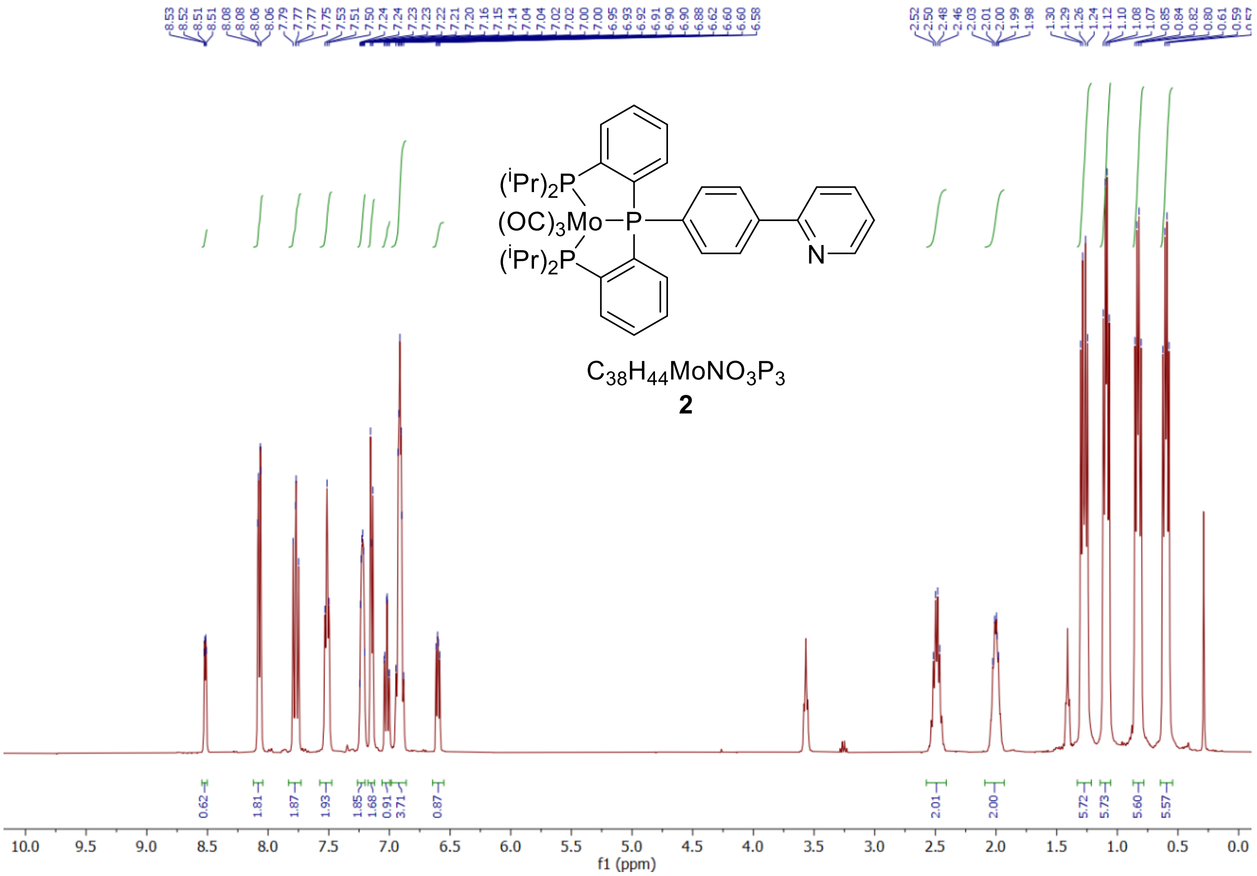

$^{31}P\{^1H\}$ NMR (162 MHz, C_6D_6)

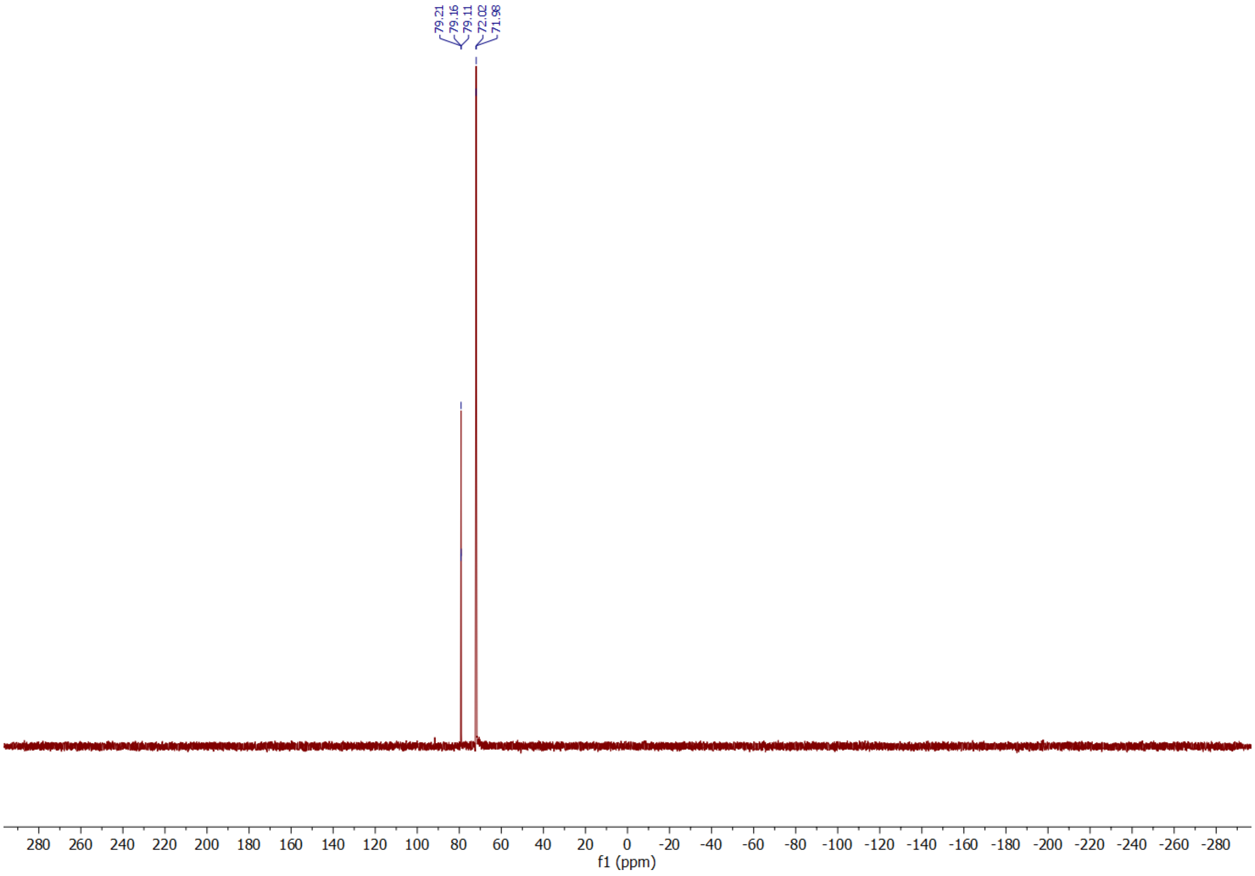

$^{13}C\{^{1}H\}$ NMR (100 MHz, C_6D_6)

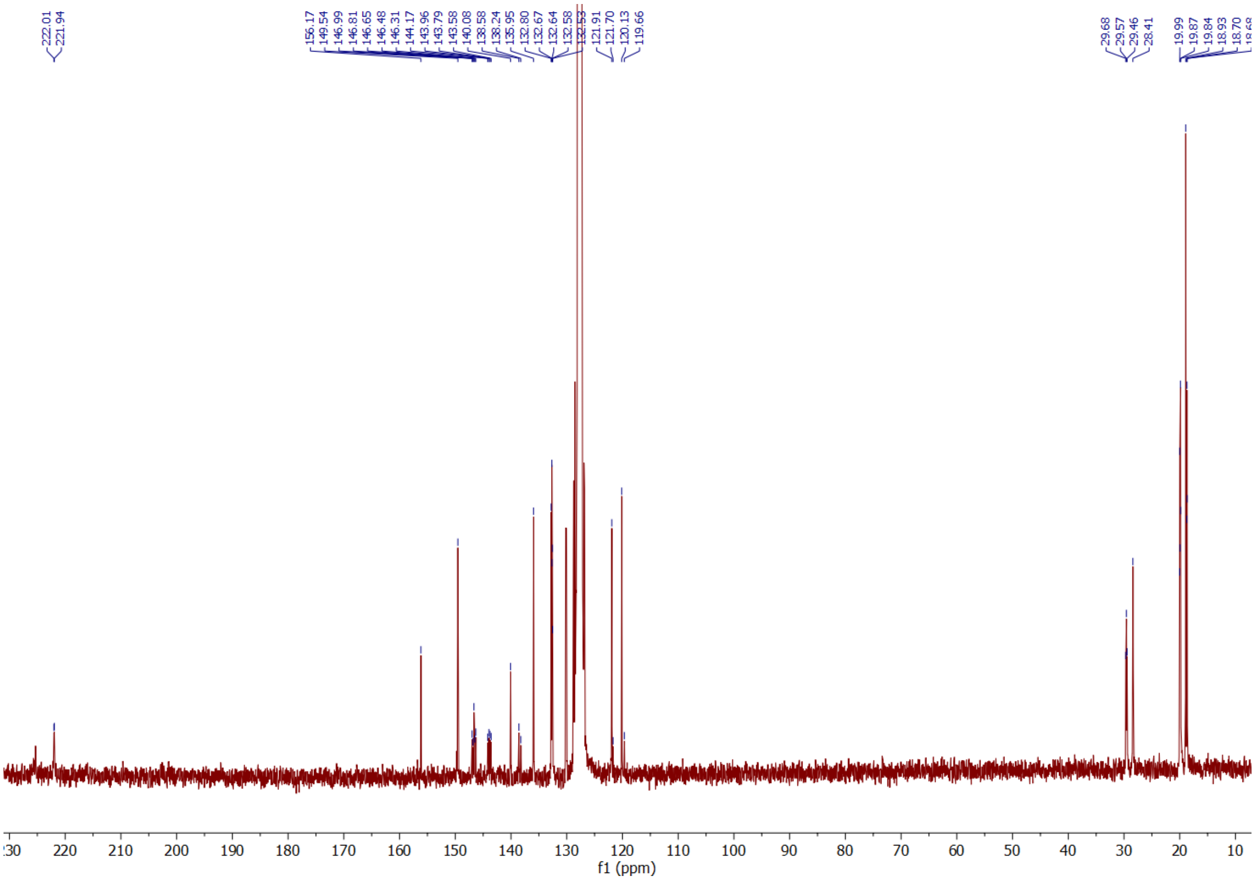

6.5. **[Mo(CO)$_3$;RhCl]** (**3**)

^{1}H NMR (400 MHz, CD_2Cl_2)

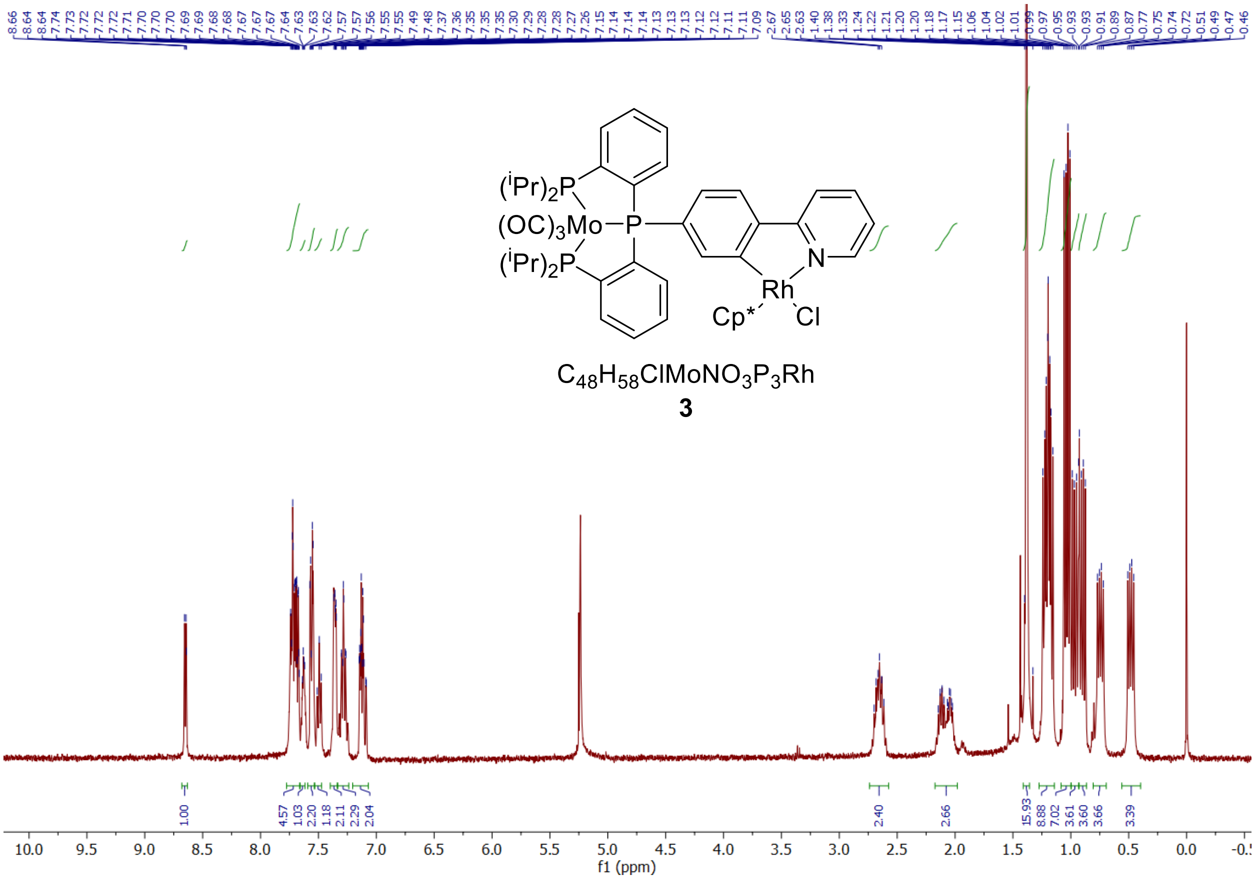

$^{31}P\{^1H\}$ NMR (162 MHz, CD_2Cl_2)

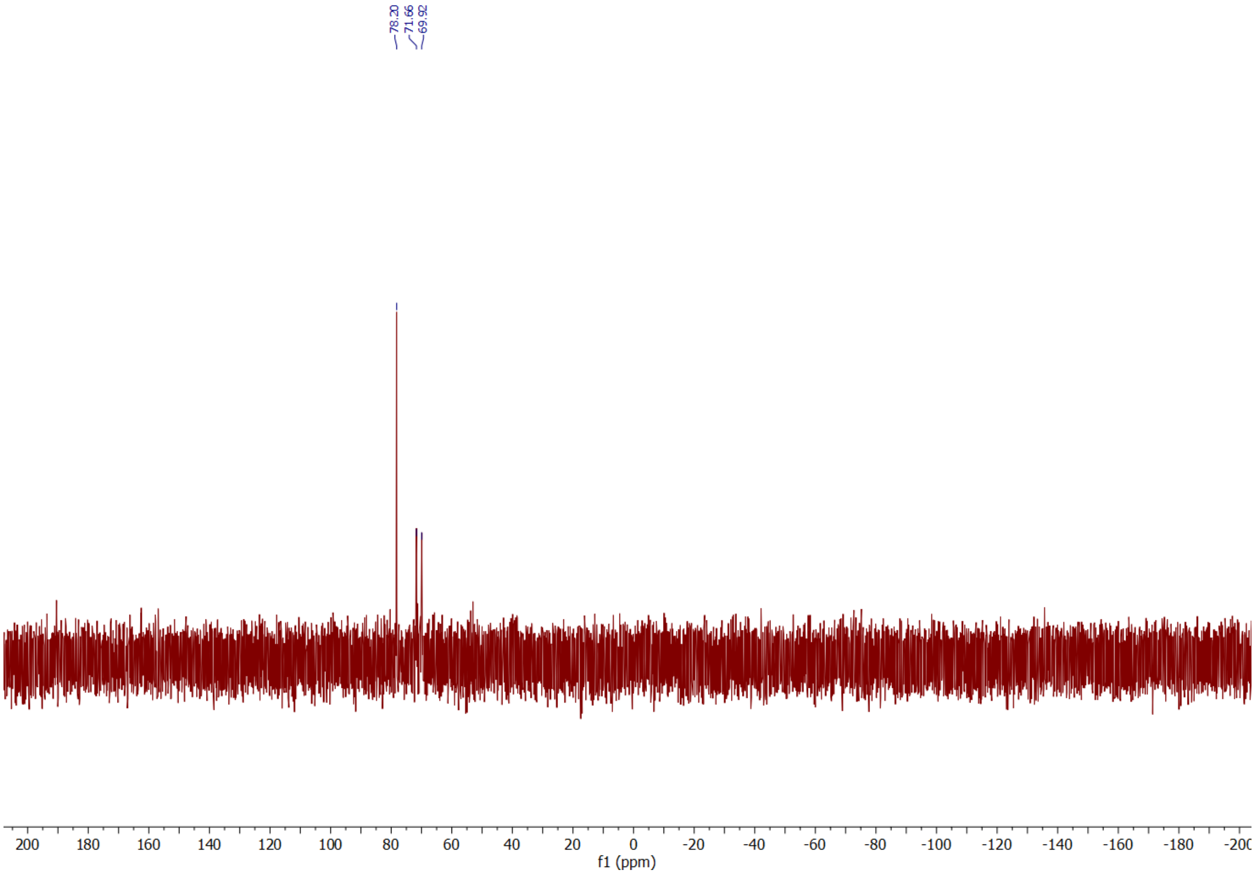

$^{13}C\{^1H\}$ NMR (100 MHz, CD_2Cl_2)

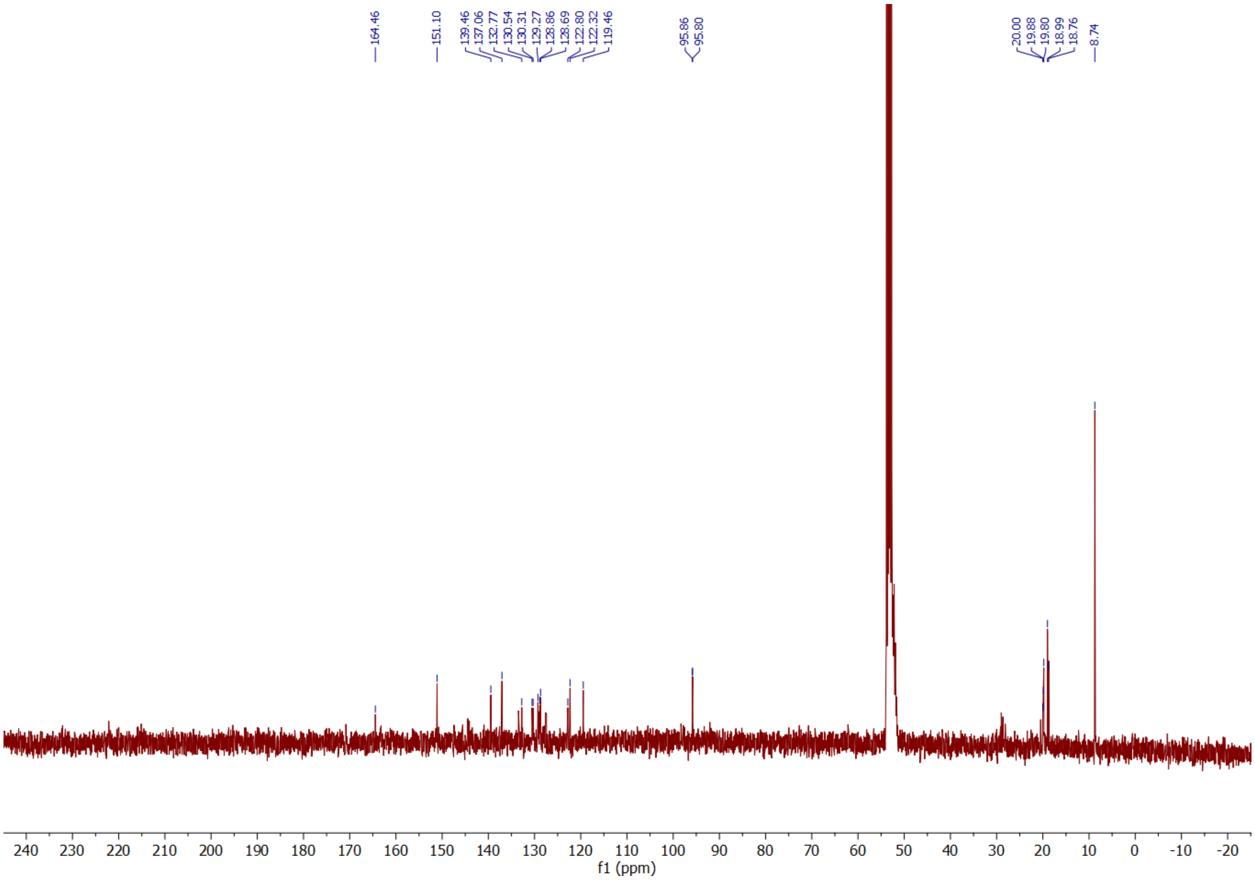

6.6. **[Mo(CO)$_3$;IrCl]** (**4**)

^{1}H NMR (400 MHz, CD_2Cl_2)

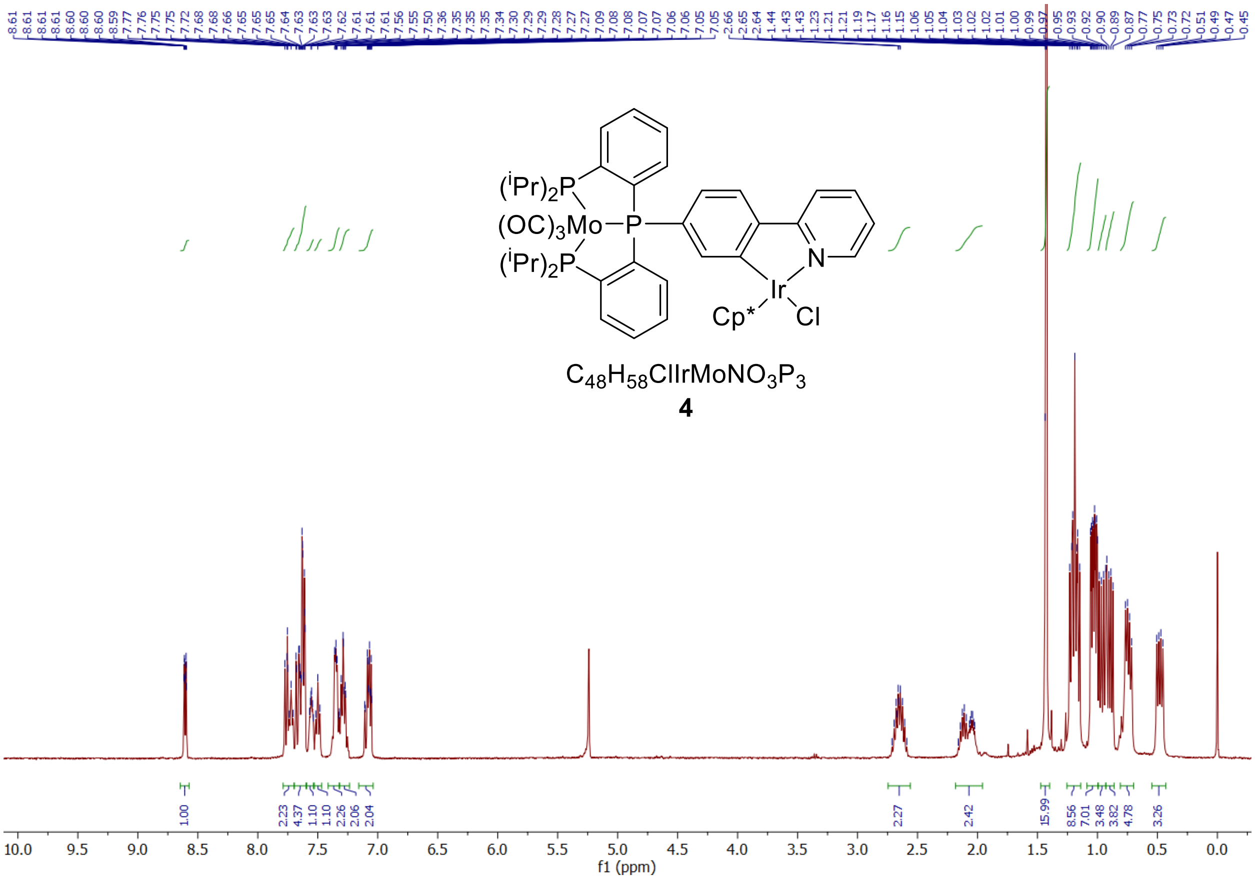

$^{31}P\{^{1}H\}$ NMR (162 MHz, CD_2Cl_2)

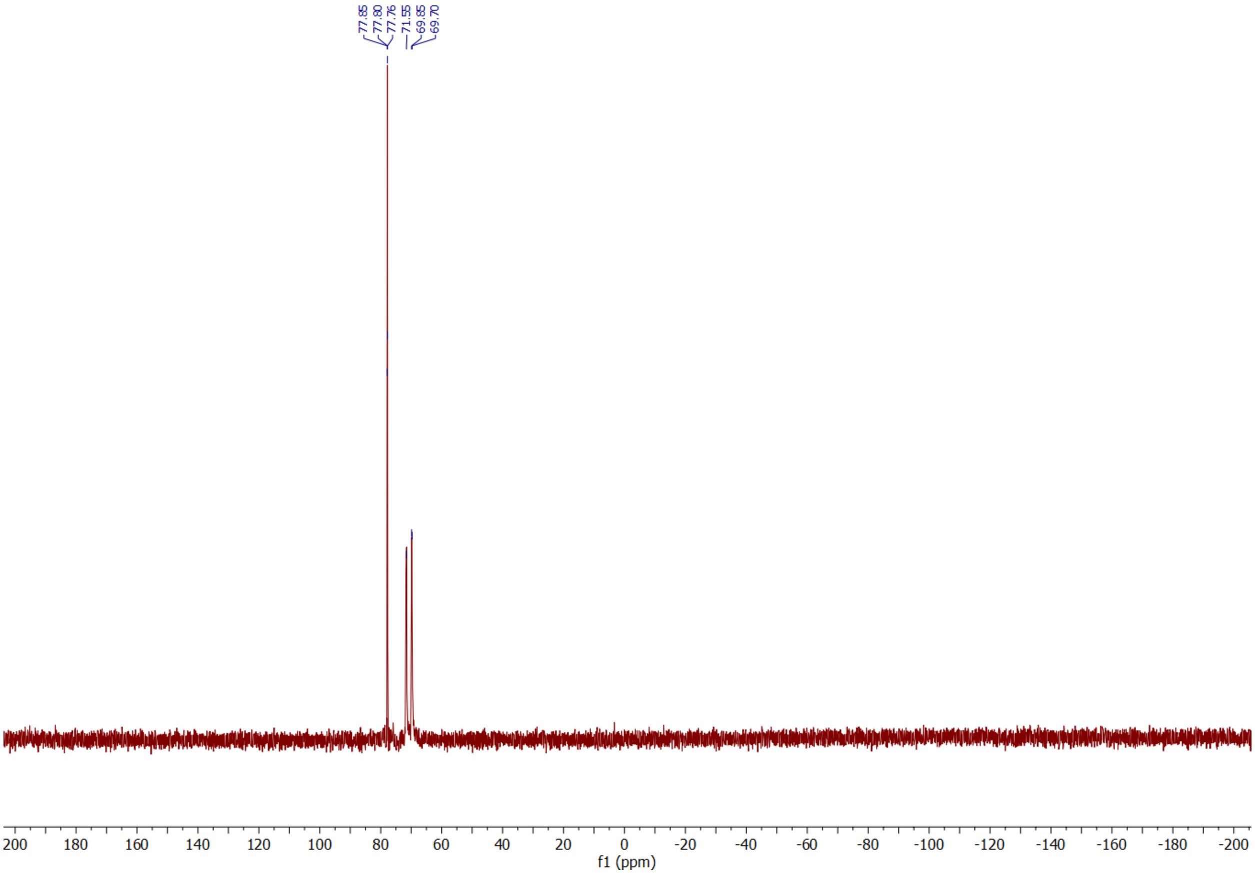

$^{13}C\{^{1}H\}$ NMR (100 MHz, CD_2Cl_2)

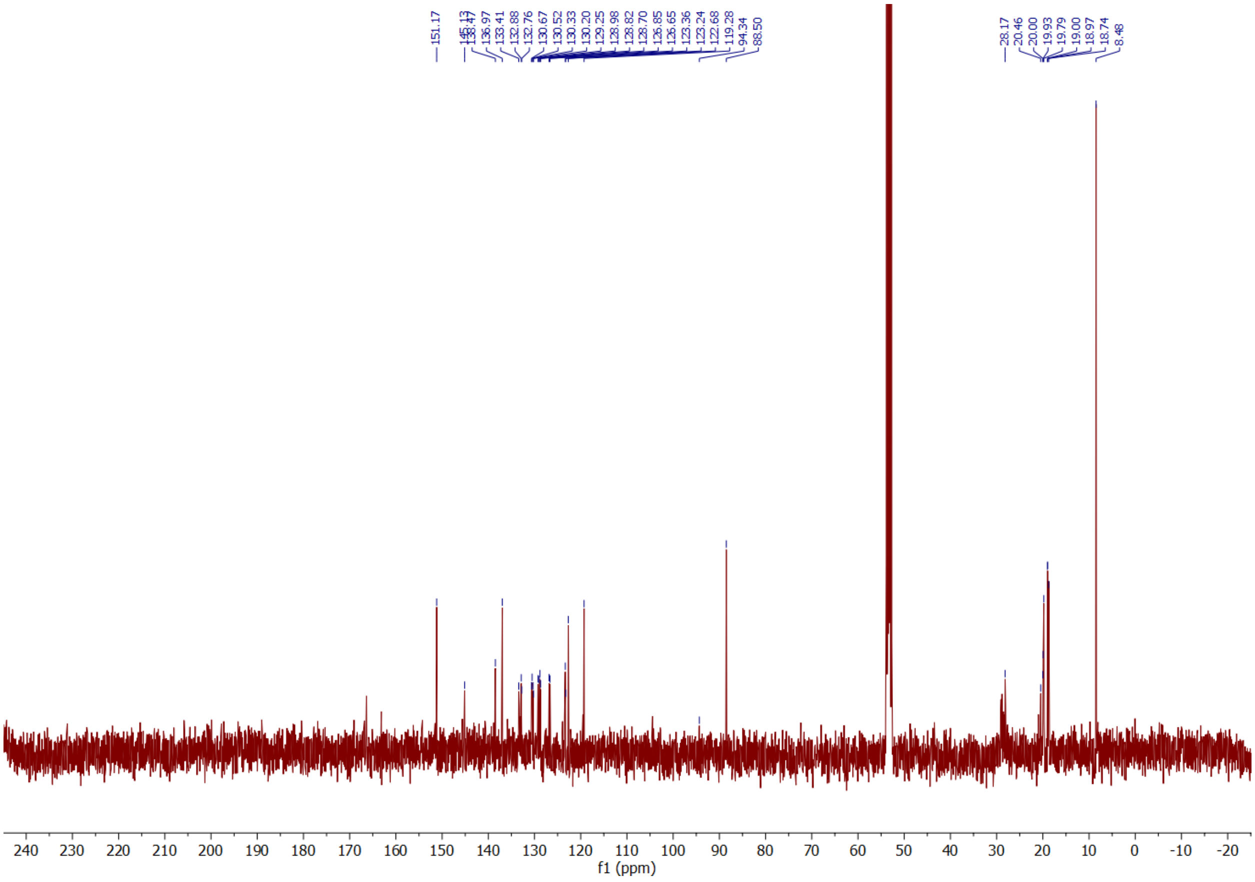

6.7. **C2**

^{1}H NMR (400 MHz, CD_3CN)

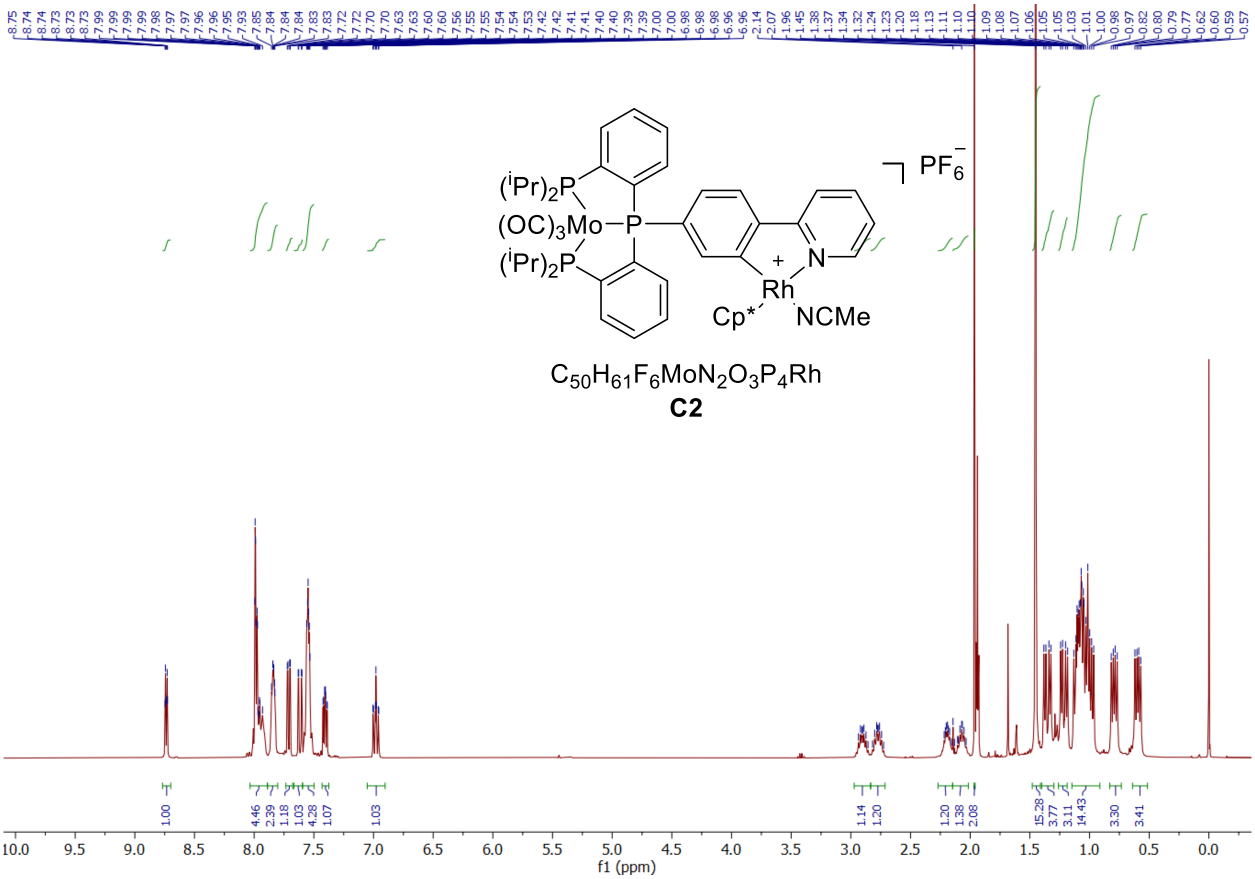

$^{31}P\{^{1}H\}$ NMR (162 MHz, CD_3CN)

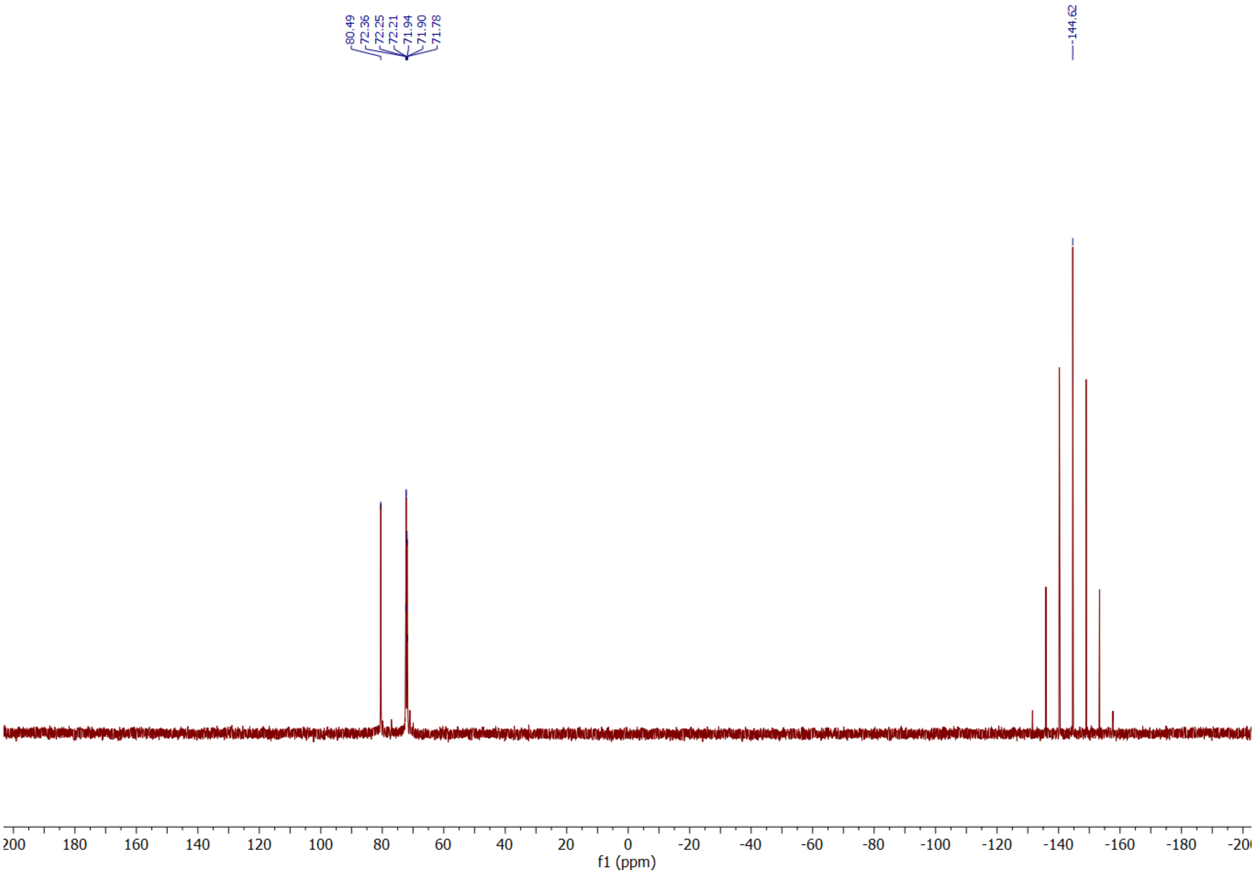

$^{13}C\{^{1}H\}$ NMR (100 MHz, CD_3CN)

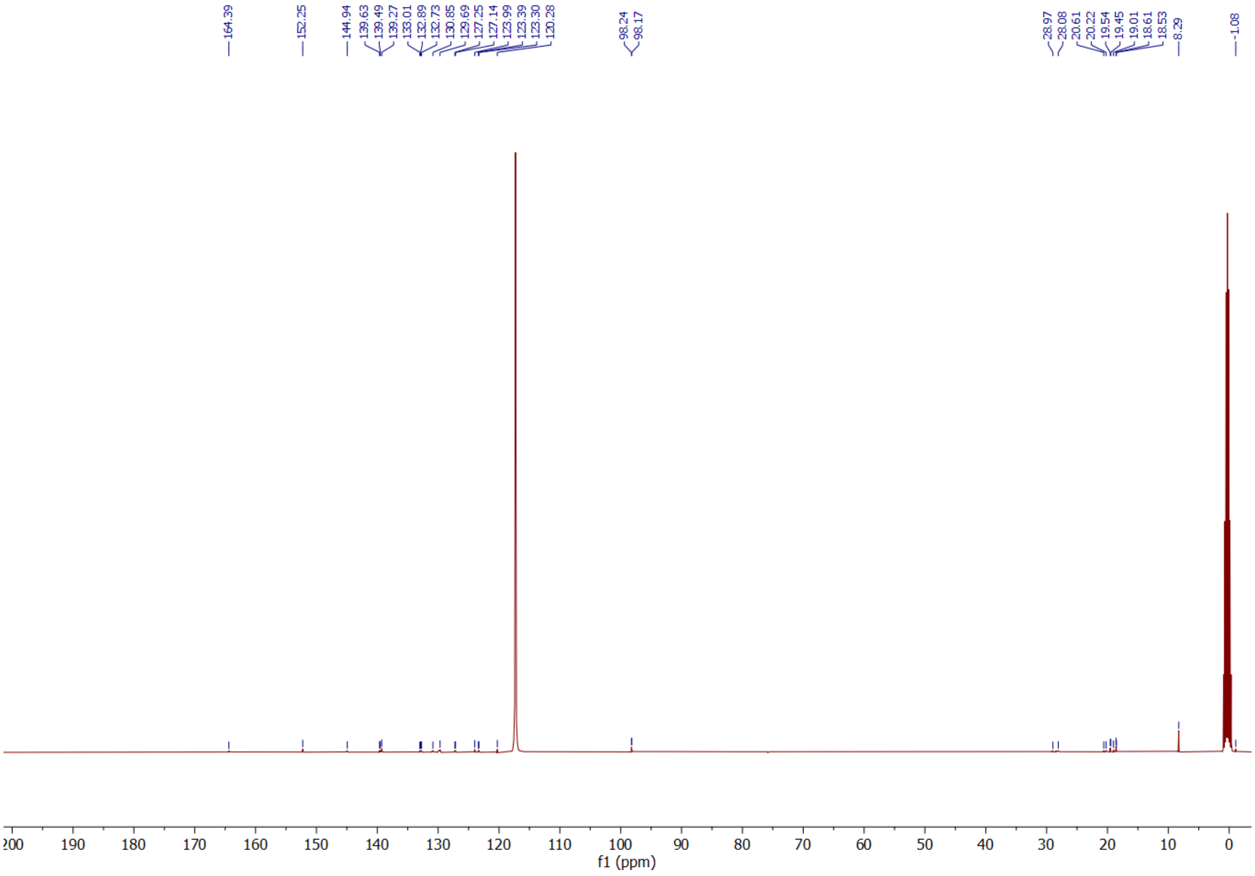

$^{19}F\{^{1}H\}$ NMR (376 MHz, CD_3CN)

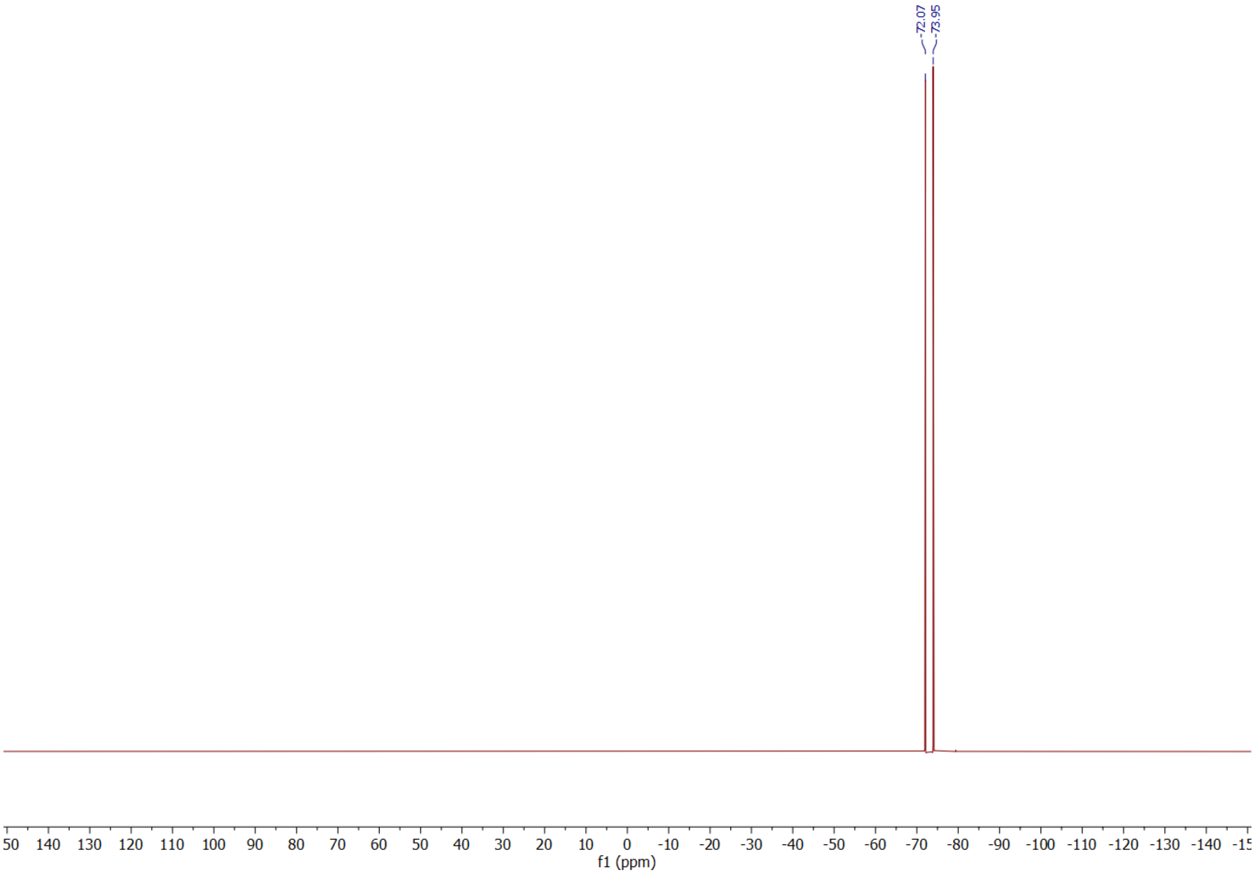

6.8. **C1**

^{1}H NMR (400 MHz, CD_3CN)

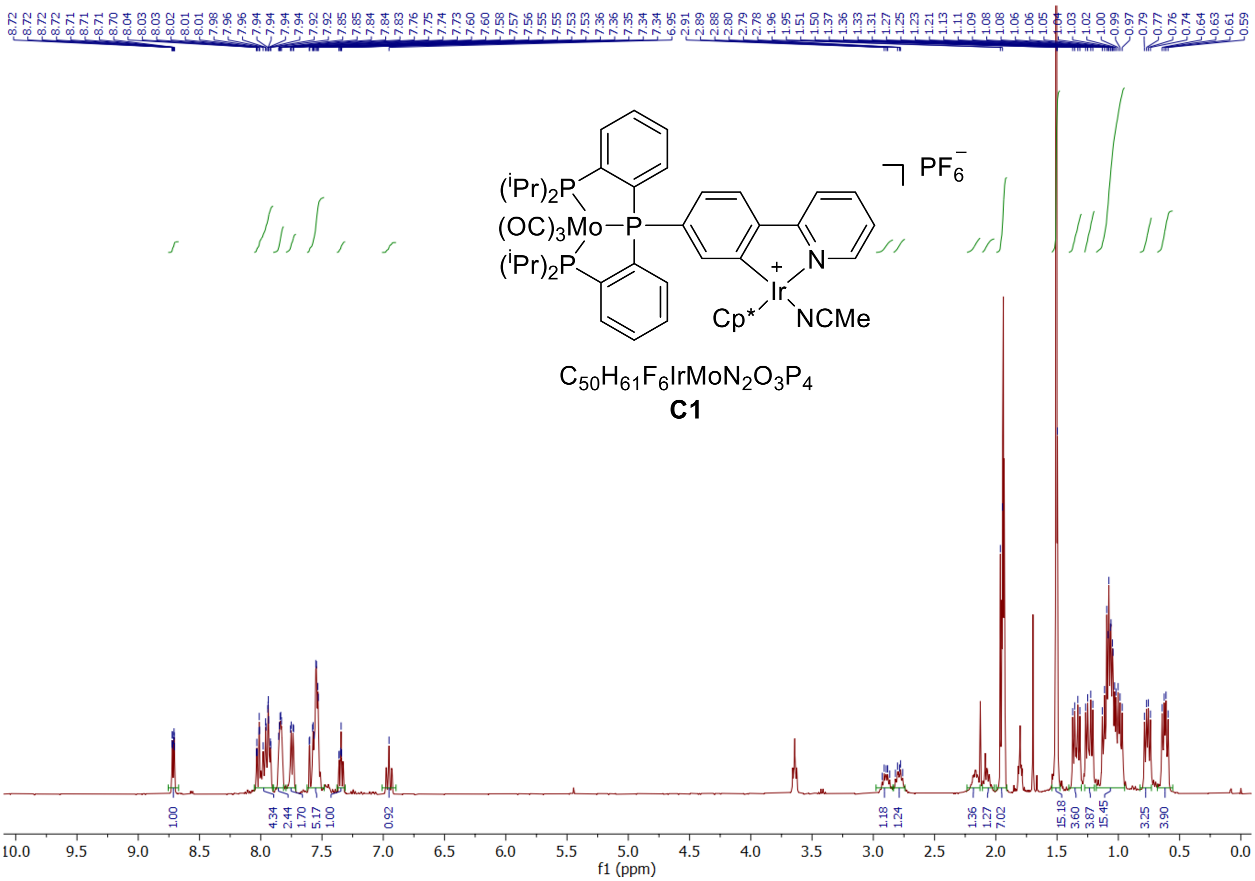

$^{31}P\{^{1}H\}$ NMR (162 MHz, CD_3CN)

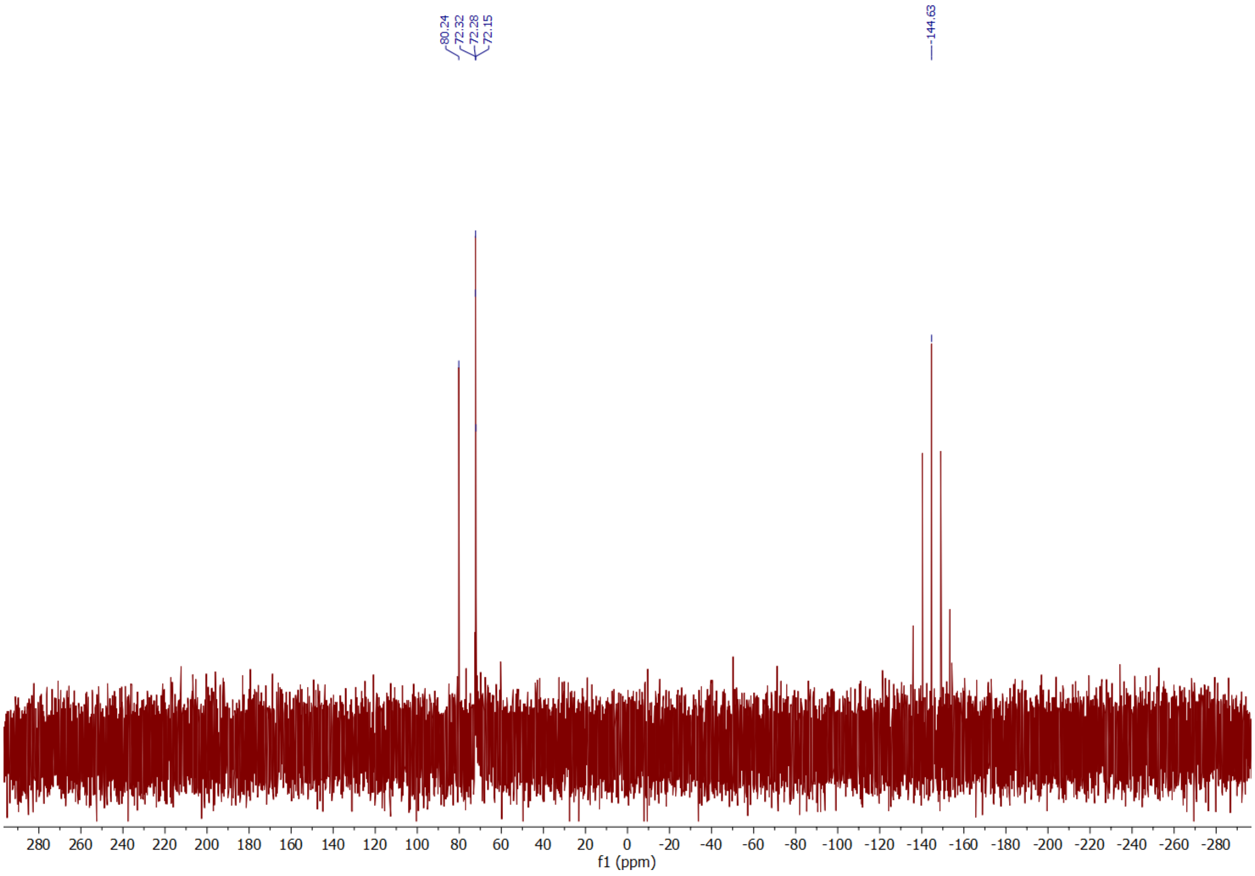

$^{13}C\{^{1}H\}$ NMR (100 MHz, CD_3CN)

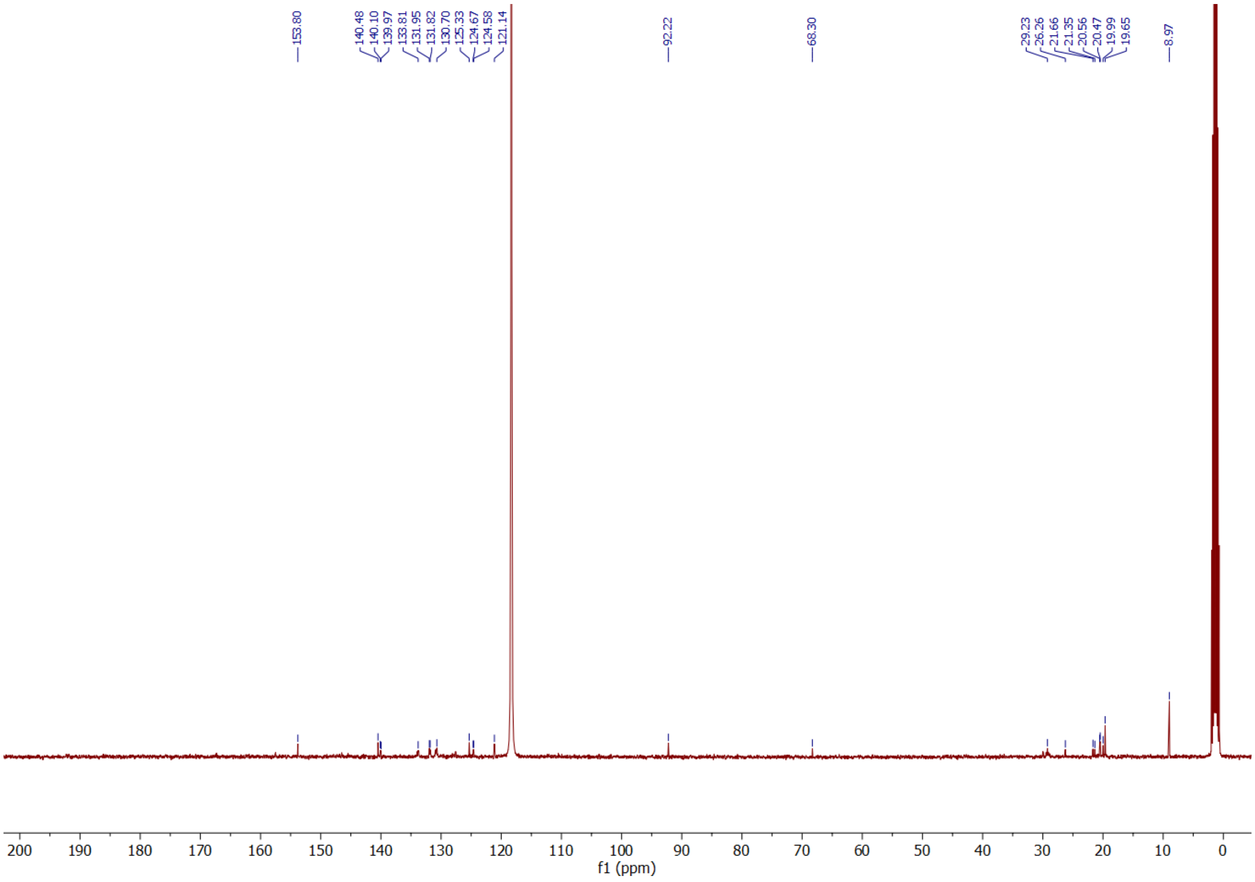

$^{19}F\{^{1}H\}$ NMR (376 MHz, CD_3CN)

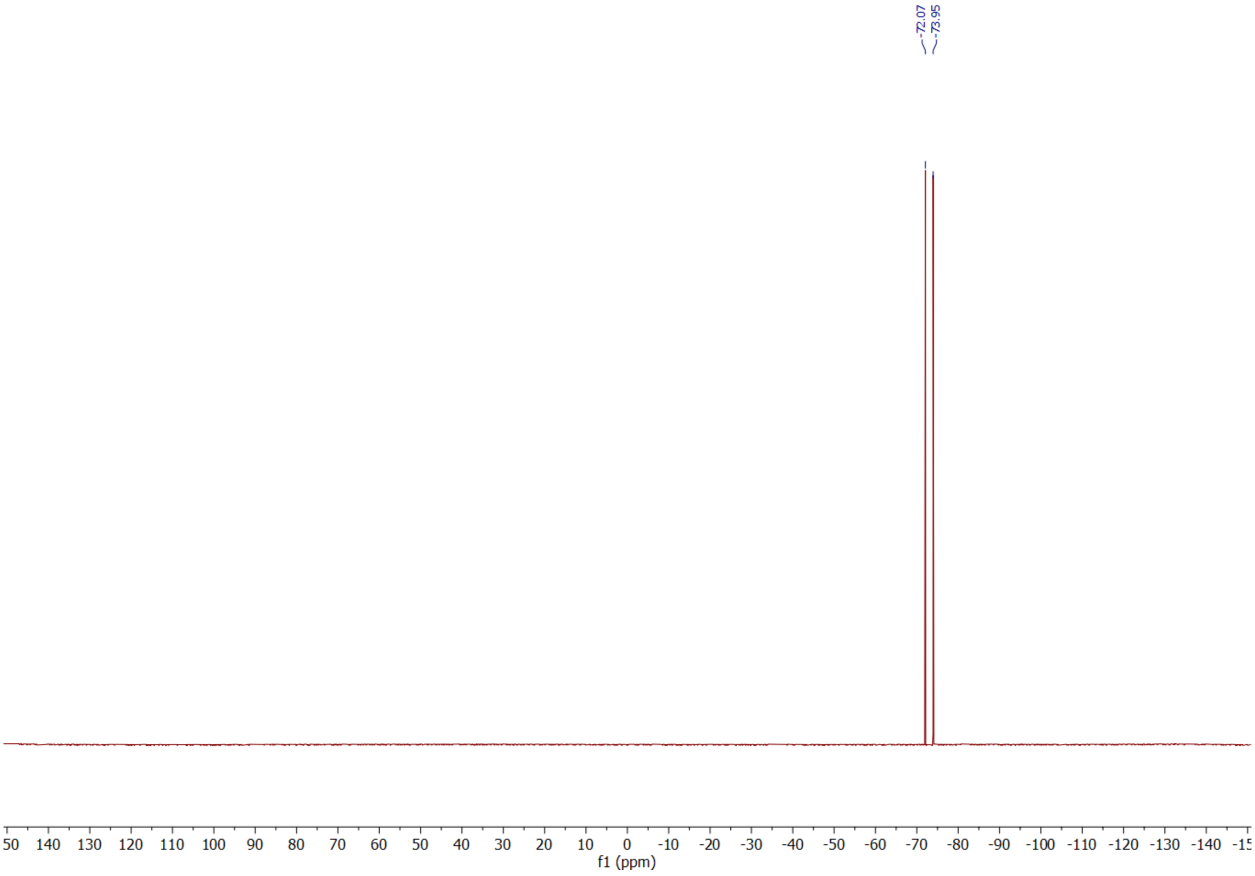

6.9. Representative ^{1}H NMR spectrum (400 MHz, D_2O) of catalytic trials

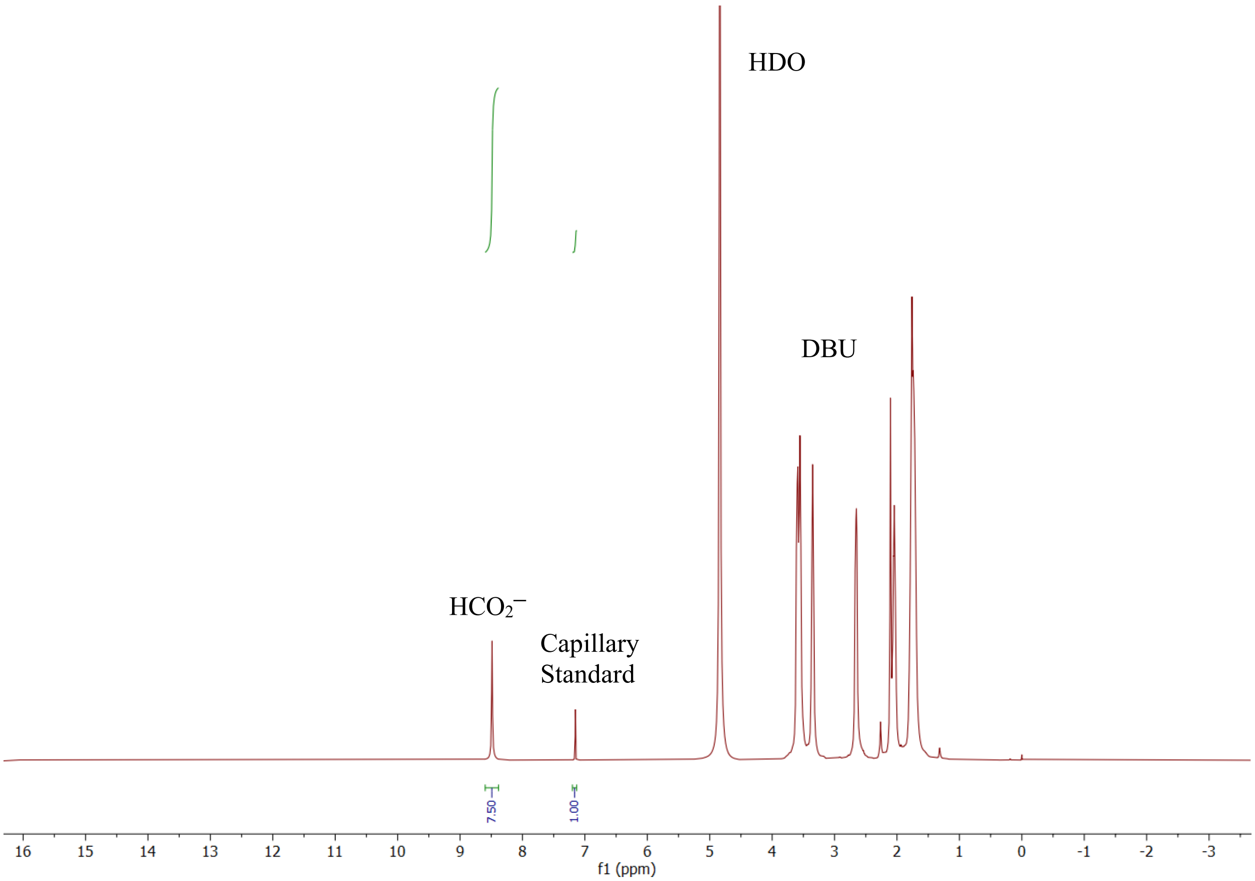

6.10. ^{1}H NMR spectrum (400 MHz, CD_3CN) of hydricity measurement of **[Ir^{III}H]** at equilibrium

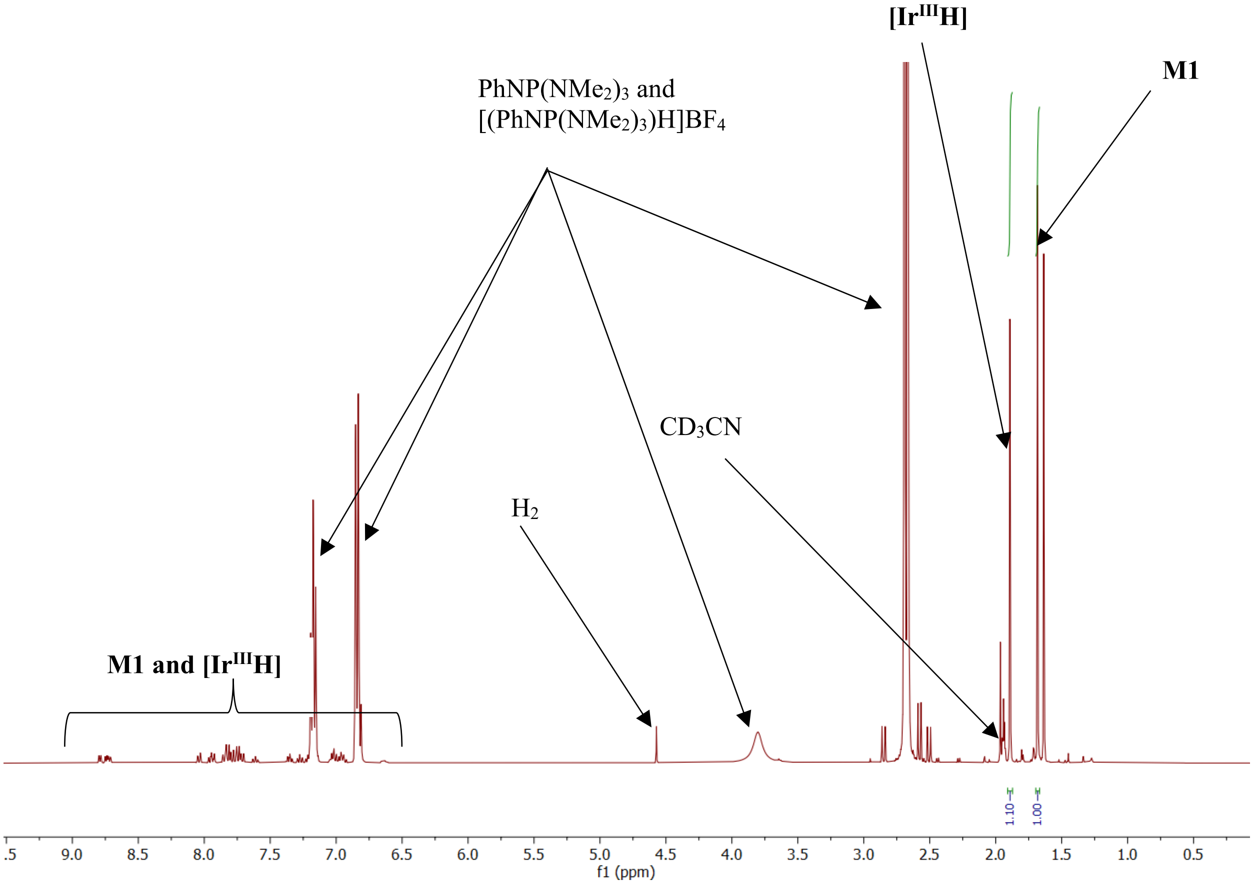

7. X-Ray Crystallography and Structure Refinement Details

COMPLEX	M3[a)]	2[b)]
EMPIRICAL FORMULA	$C_{33}H_{41}MoO_3P_3$	$C_{38}H_{44}MoNO_3P_3$
FORMULA WEIGHT	674.51	751.59
TEMPERATURE	130(2) K	130(2) K
WAVELENGTH	71.073 pm	71.073 pm
CRYSTAL SYSTEM	Monoclinic	Monoclinic
SPACE GROUP	$P2_1/c$	C2/c
UNIT CELL DIMENSIONS	a = 1632.26(3) pm α = 90°<br>b = 1091.79(1) pm ß = 98.153(1)°<br>c = 3625.61(5) pm γ = 90°	a = 3907.6(3) pm α = 90°<br>b = 1166.00(6) pm ß = 100.657(6)°<br>c = 1604.35(9) pm γ = 90°
VOLUME	6.3958(2) nm^3	7.1838(7) nm^3
Z	8	8
DENSITY (CALCULATED)	1.401 Mg/m^3	1.390 Mg/m^3
ABSORPTION COEFFICIENT	0.592 mm^{-1}	0.536 mm^{-1}
F(000)	2800	3120
CRYSTAL SIZE	0.20 x 0.20 x 0.07 mm^3	0.10 x 0.05 x 0.01 mm^3
THETA RANGE	1.572 to 28.057°	1.825 to 28.058°
INDEX RANGES	$-19 \leq h \leq 20$, $-14 \leq k \leq 14$, $-45 \leq l \leq 47$	$-47 \leq h \leq 51$, $-15 \leq k \leq 15$, $-20 \leq l \leq 21$
REFLECTIONS COLLECTED	64545	33909
INDEPENDENT REFLECTIONS	14435 [R(int) = 0.0602]	7776 [R(int) = 0.1913]
COMPLETENESS TO THETA	99.9%	99.9%
ABSORPTION CORRECTION	Semi-empirical from equivalents	Semi-empirical from equivalents
MAX. AND MIN. TRANSMISSION	1.00000 and 0.98853	1.00000 and 0.56603
REFINEMENT METHOD	Full-matrix least-squares on F^2	Full-matrix least-squares on F^2
RESTRAINTS / PARAMETERS	90 / 797	0 / 423
GOODNESS-OF-FIT ON F^2	1.037	0.983
FINAL R INDICES [I>Σ(I)]	R1 = 0.0458, wR2 = 0.0852	R1 = 0.0710, wR2 = 0.1116
R INDICES (ALL DATA)	R1 = 0.0745, wR2 = 0.0961	R1 = 0.1811, wR2 = 0.1490
RESIDUAL ELECTRON DENSITY	0.802 and -0.402 $e \cdot Å^{-3}$	0.866 and -1.326 $e \cdot Å^{-3}$
CCDC NUMBER	2210067	2210068

Comments: [a)] Three isopropyl substituents are disordered with a ratio of C(25), C(26), C(27) = 0.704(7):0.296(7), C(28), C(29), C(30) = 0.577(7):0.423(7) and C(55), C(56), C(57) = 0.542(7):0.458(7). [b)] It was very difficult to find and isolate a suitable single crystal with an acceptable quality. For the small sized crystal the results are acceptable.

COMPLEX	[Mo(CO)$_3$;RhCl] (3)[a)]	[Mo(CO)$_3$;IrCl] (4)[b)]
EMPIRICAL FORMULA	$C_{50.61}H_{63.63}Cl_{4.19}MoNO_3P_3Rh$	$C_{49.50}H_{61}Cl_4IrMoNO_3P_3$
FORMULA WEIGHT	1174.09	1240.84
TEMPERATURE	130(2) K	130(2) K
WAVELENGTH	71.073 pm	71.073 pm
CRYSTAL SYSTEM	Triclinic	Monoclinic
SPACE GROUP	$P\bar{1}$	$P2_1/n$
UNIT CELL DIMENSIONS	a = 1178.35(2) pm α = 94.009(2)°<br>b = 1322.26(3) pm β = 99.330(2)°<br>c = 3556.69(6) pm γ = 103.938(2)°	a = 1884.95(3) pm α = 90°<br>b = 1541.75(2) pm β = 116.672(2)°<br>c = 1974.79(3) pm γ = 90°
VOLUME	5.2729(2) nm^3	5.1283(2) nm^3
Z	4	4
DENSITY (CALCULATED)	1.479 Mg/m^3	1.607 Mg/m^3
ABSORPTION COEFFICIENT	0.895 mm^{-1}	3.180 mm^{-1}
F(000)	2406	2484
CRYSTAL SIZE	0.40 x 0.20 x 0.05 mm^3	0.46 x 0.30 x 0.15 mm^3
THETA RANGE FOR DATA	1.798 to 31.698°.	1.791 to 32.191°.
INDEX RANGES	$-16 \leq h \leq 17$, $-19 \leq k \leq 19$, $-52 \leq l \leq 50$	$-27 \leq h \leq 28$, $-22 \leq k \leq 23$, $-29 \leq l \leq 28$
REFLECTIONS COLLECTED	84114	74239
INDEPENDENT REFLECTIONS	33406 [R(int) = 0.0486]	16973 [R(int) = 0.0573]
COMPLETENESS TO THETA	99.9%	99.9%
ABSORPTION CORRECTION	Semi-empirical from equivalents	Analytical
MAX. AND MIN. TRANSMISSION	1.00000 and 0.97020	0.678 and 0.411
REFINEMENT METHOD	Full-matrix least-squares on F^2	Full-matrix least-squares on F^2
RESTRAINTS / PARAMETERS	79 / 1225	72 / 616
GOODNESS-OF-FIT ON F^2	1.049	1.075
FINAL R INDICES [I>Σ(I)]	R1 = 0.0496, wR2 = 0.1142	R1 = 0.0472, wR2 = 0.1116
R INDICES (ALL DATA)	R1 = 0.0691, wR2 = 0.1263	R1 = 0.0753, wR2 = 0.1275
RESIDUAL ELECTRON DENSITY	1.130 and -1.462 e·Å^{-3}	3.811 and -1.867 e·Å^{-3}
CCDC NUMBER	2210069	2210070

Comments: [a)] The compound crystallized from a methylene chloride/*n*-pentane solution. The two methylene chloride molecules (Cl7, Cl8, C99 and Cl9, Cl10, C100) are disordered with one *n*-pentane molecule (C101 to C105) with a ratio of 0.593(3):0.407(3), i.e. 0.593 mol disordered CH_2Cl_2 molecules and 0.407 / 2 = 0.204 mol disordered *n*-pentane molecules. All attempts to describe this disorder as a pure methylene chloride disorder failed and gave unacceptable results. [b)] The iridium coordinated part of the molecules may be marginally disordered with a ratio of 0.9628(8):0.0372(8). For the minor 4% disordered part only the most electron-rich iridium center could be located. All solvent molecules (CH_2Cl_2) are disordered to a certain extent, and especially the one in the vicinity of the center

of inversion was difficult to locate within a reasonable accuracy. The total amount of all solvent molecules was confirmed with a SQUEEZE electron count [20].

COMPLEX	$[Mo^0(CO)_3;Rh^{III}(CO)]PF_6$ a)	$[Mo^0(CO)_3;Ir^{III}(N_2)]PF_6$ b)
EMPIRICAL FORMULA	$C_{56}H_{72}F_6MoNO_{5.75}P_4Rh$	$C_{55}H_{72}F_6IrMoN_3O_{4.75}P_4$
FORMULA WEIGHT	1287.87	1377.17
TEMPERATURE	130(2) K	130(2) K
WAVELENGTH	71.073 pm	71.073 pm
CRYSTAL SYSTEM	Orthorhombic	Orthorhombic
SPACE GROUP	Pbca	Pbca
UNIT CELL DIMENSIONS	a = 1835.14(2) pm α = 90°<br>b = 1638.00(2) pm β = 90°<br>c = 3831.54(5) pm γ = 90°	a = 1840.61(2) pm α = 90°<br>b = 1644.88(2) pm β = 90°<br>c = 3844.42(4) pm γ = 90°
VOLUME	11.5175(2) nm^3	11.6393(2) nm^3
Z	8	8
DENSITY (CALCULATED)	1.485 Mg/m^3	1.572 Mg/m^3
ABSORPTION COEFFICIENT	0.683 mm^{-1}	2.676 mm^{-1}
F(000)	5296	5552
CRYSTAL SIZE	0.20 x 0.15 x 0.10 mm^3	0.30 x 0.20 x 0.02 mm^3
THETA RANGE FOR DATA	1.977 to 26.914°	1.970 to 26.727°
INDEX RANGES	$-22 \leq h \leq 22$, $-19 \leq k \leq 19$, $-48 \leq l \leq 47$	$-21 \leq h \leq 23$, $-20 \leq k \leq 19$, $-48 \leq l \leq 48$
REFLECTIONS COLLECTED	61110	107782
INDEPENDENT REFLECTIONS	11563 [R(int) = 0.0623]	11732 [R(int) = 0.0579]
COMPLETENESS TO THETA	99.9%	99.9%
ABSORPTION CORRECTION	Semi-empirical from equivalents	Semi-empirical from equivalents
MAX. AND MIN. TRANSMISSION	1.00000 and 0.92073	1.00000 and 0.68575
REFINEMENT METHOD	Full-matrix least-squares on F^2	Full-matrix least-squares on F^2
RESTRAINTS / PARAMETERS	184 / 667	211 / 691
GOODNESS-OF-FIT ON F^2	1.190	1.115
FINAL R INDICES [I>Σ(I)]	R1 = 0.0701, wR2 = 0.1316	R1 = 0.0568, wR2 = 0.1064
R INDICES (ALL DATA)	R1 = 0.0886, wR2 = 0.1378	R1 = 0.0768, wR2 = 0.1135
RESIDUAL ELECTRON DENSITY	0.935 and -0.587 $e \cdot Å^{-3}$	2.170 and -1.472 $e \cdot Å^{-3}$
CCDC NUMBER	2210071	2210072

Comments: a) Two ^{i}Pr substituents (C30 to C36) are disordered with a ratio of 0.55(1):0.45(1). The sample was crystallized from THF with small amounts of *n*-hexane. Attempts to locate disordered solvent molecules with a reasonable accuracy failed. The electron density for these highly disordered solvent molecules has been removed with the SQUEEZE routine implemented in PLATON the squeeze electron count suggests the electron density of approximately 14 poorly defined and diffusely oriented THF molecules in the unit cell [20]. These molecules represent

an electron density of 560 (14 × 40) electrons. This value is in accordance with the estimated "squeezed" electron density of 576 electrons. The "squeezed volume" for this structure is in the range of approximately 2.069 nm^3 (2069 $Å^3$) for the unit cell. A volume of 148 $Å^3$ for one THF molecule (approximately 30 $Å^3$ for each non-hydrogen atom) is acceptable for loosely packed and disordered solvent molecules. The given formula has been corrected for this solvent contribution. Rhodium is most likely coordinated by CO. The molecular formula can be given as $[RhMo(CO)_4(C_{35}H_{43}NP_3)(C_{10}H_{15})]$ $[PF_6]$ · 1.75 THF. [b)]Anisotropic refinement of all non-hydrogen atoms, except the disordered N_2 molecule in the vicinity of iridium and the minor 12% fraction of the disordered PF_6^- anion, with SHELXL-2018 [11,21]. All hydrogen atoms were calculated on idealized positions. The N_2 ligand, two ^{i}Pr substituents and the PF_6^- anion are disordered. In detail: N2, N3: 0.59(3):0.41(3); C30 to C36: 0.49(1):0.51(1) and P4, F1 to F6: 0.88(1):0.12(1). The sample was crystallized from THF with small amounts of *n*-hexane. Attempts to locate disordered solvent molecules with a reasonable accuracy failed. The electron density for these highly disordered solvent molecules has been removed with the SQUEEZE routine implemented in PLATON; the squeeze electron suggests the electron density of approximately 14 poorly defined and diffusely oriented THF molecules from the unit cell [20]. These molecules represent an electron density of 566 electrons. This value is in accordance with the estimated "squeezed" electron density of 560 (14 × 40) electrons. The "squeezed volume" for this structure is in the range of approximately 2.130 nm^3 (2130 $Å^3$) for the unit cell. A volume of 152 $Å^3$ for one THF molecule (approximately 30 $Å^3$ for each non-hydrogen atom) is acceptable for loosely packed and disordered solvent molecules. The given formula has been corrected for this solvent contribution. Iridium was surprisingly not coordinated by the solvent used for the synthesis (THF and/or acetonitrile). From the experimental results we inferred that dinitrogen, which was in use as a protecting gas, is coordinating end-on and side-on to iridium in a disordered fashion. The molecular formula can be given as $[IrMo(CO)_3(N_2)(C_{35}H_{43}NP_3)(C_{10}H_{15})]$ $[PF_6]$ · 1.75 THF.

8. Electrochemical Data

8.1. Cyclic Voltammogram of **M3**[a)]

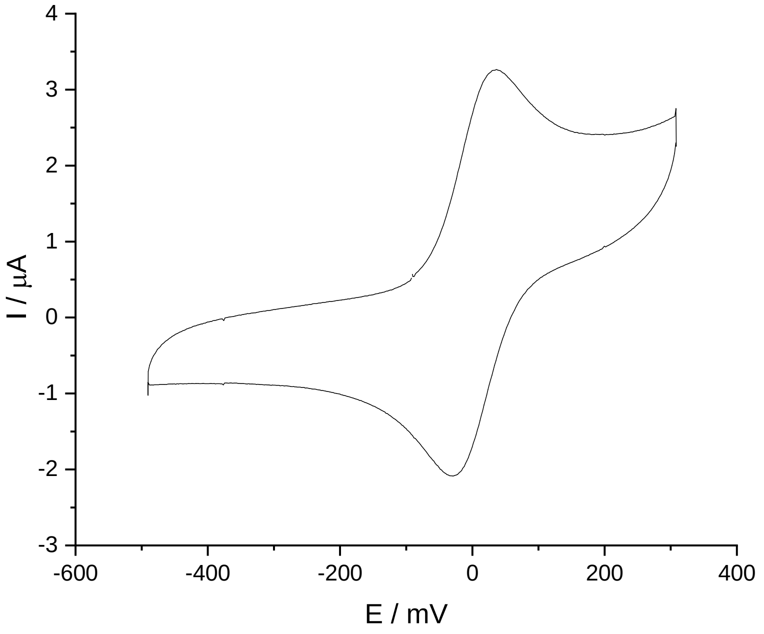

[a)] Reaction conditions: concentration 1 mM in 0.1 M [$^{n}Bu_4N]PF_6$ in MeCN.

8.2. Cyclic Voltammogram of **M3** and **M1**[a)]

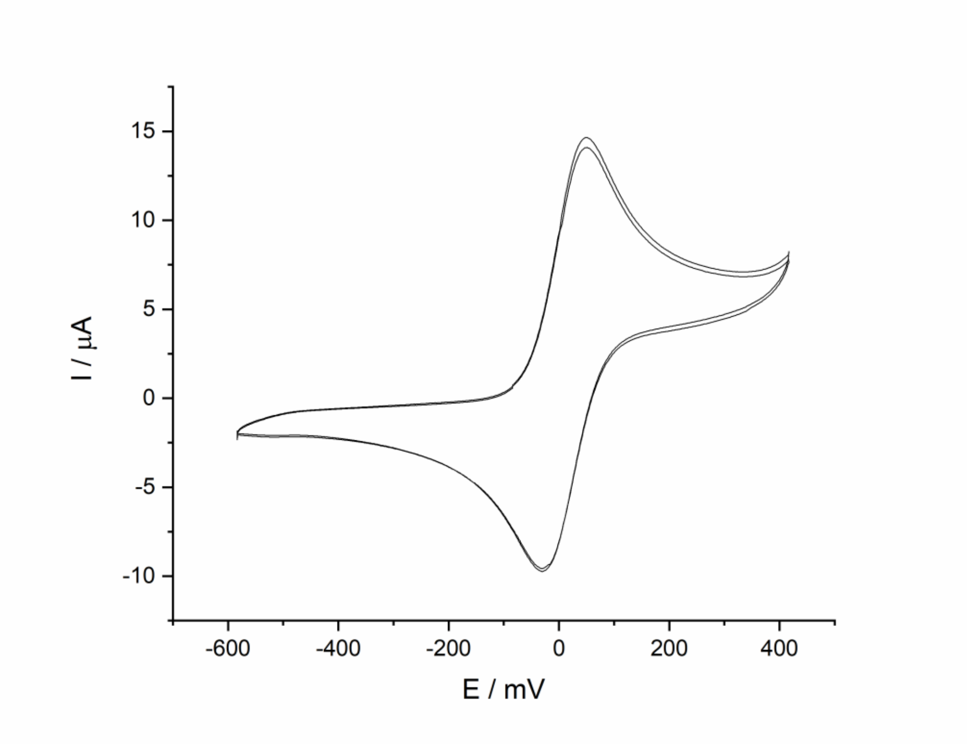

a) Reaction conditions: concentration 3 mM each in 0.1 M $[^{n}Bu_4N]PF_6$ in MeCN.

8.3. Cyclic Voltammogram of **M3** and **M2**[a)]

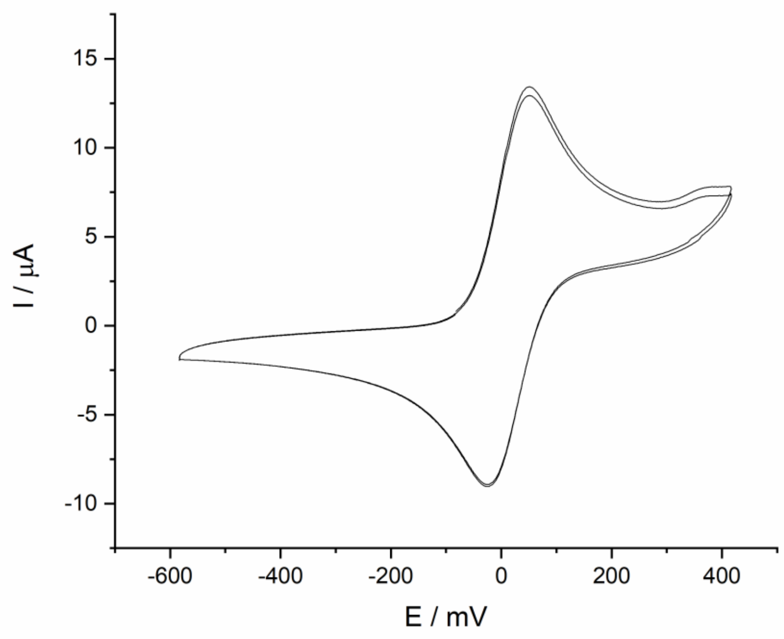

a) Reaction conditions: concentration 3 mM each in 0.1 M [$^n Bu_4 N$]PF_6 in MeCN.

8.4. Cyclic Voltammogram of **C2**[b)]

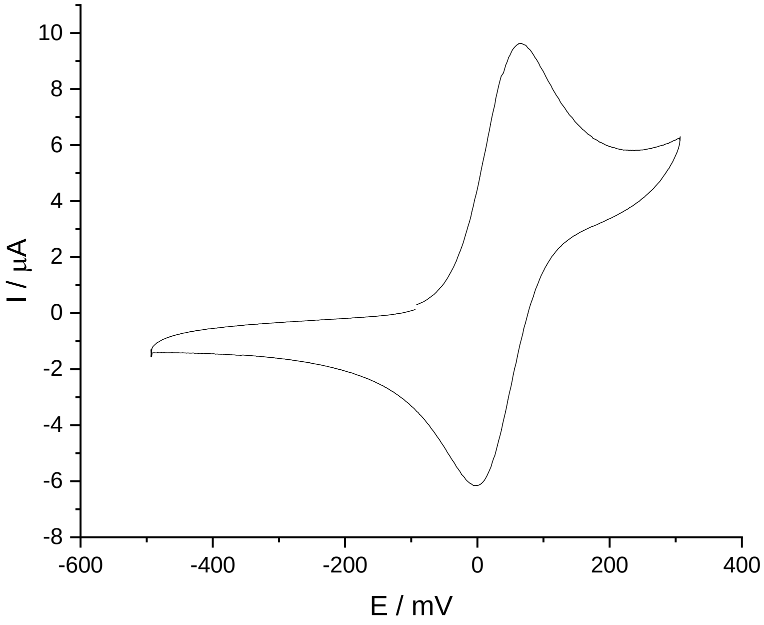

[b)]Reaction conditions: concentration 3 mM in 0.1 M [$^{n}Bu_4N]PF_6$ in MeCN.

8.5. Cyclic Voltammogram of **C1**[c)]

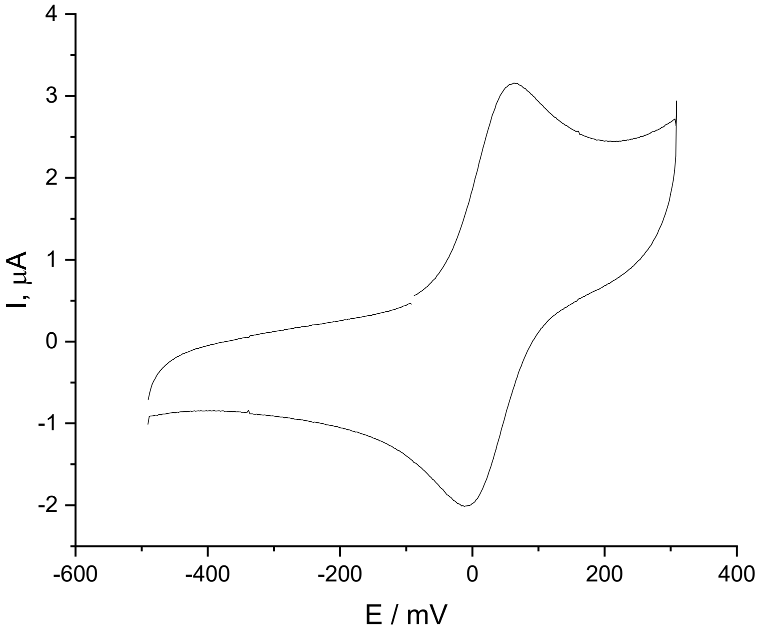

[c)]Reaction conditions: concentration 1 mM in 0.1 M [$^nBu_4N]PF_6$ in MeCN.

9. Gibbs Free Energies

Intermediate	Base	MeCN (M1)	DMSO	H_2O	MeOH
**$[Ir^{III}(solv)]^+$**	DBU	0.0	0.0	0.0	0.0
	NEt_3	0.0	0.0	0.0	0.0
**$[Ir^{III}(base)]^+$**	DBU	-48.4	-25.2	-65.9	-68.9
	NEt_3	15.7	37.8	-2.1	-4.6
**$[Ir^{III}(\eta^2-H_2)]^+$**	DBU	49.4	71.4	31.3	29.2
	NEt_3	49.4	71.4	31.3	29.2
**$[Ir^{III}H]$**	DBU	-64.0	-42.3	-82.8	-84.1
	NEt_3	-16.4	5.0	-35.9	-36.4
**TS3**	DBU	70.7	92.0	52.0	50.7
	NEt_3	118.2	139.3	98.9	98.4
**$[Ir^{III}HCO_2]$**	DBU	-18.8	2.7	-38.1	-38.8
	NEt_3	28.8	50.0	8.9	8.9
**$[Ir^{III}(solv)]^+$ a)**	DBU	5.2	5.3	5.3	5.2
	NEt_3	18.4	18.4	18.3	18.5

a) With considering the formation of the [[DBU(H)]HCO_2 salt.

10. References

1. Bellan, J.; Marre, M.R.; Sanchez, M.; Wolf, R. Nouveaux ylures du phosphore a partir e tris(amino)iminophosphanes et d'esters acetyleniques. etude structurale par RMN de ^{13}C. *Phosphorus Sulfur Silicon Relat. Elem.* **1981**, *12*, 11–18. https://doi.org/10.1080/03086648108078285.
2. Buscagan, T.M.; Oyala, P.H.; Peters, J.C. N_2 -to-NH_3 Conversion by a triphos-Iron Catalyst and Enhanced Turnover under Photolysis. *Angew. Chem. Int. Ed.* **2017**, *56*, 6921–6926. https://doi.org/10.1002/ anie.201703244.
3. Mankad, N.P.; Rivard, E.; Harkins, S.B.; Peters, J.C. Structural snapshots of a flexible Cu_2P_2 core that accommodates the oxidation states Cu^ICu^I, $Cu^{1.5}Cu^{1.5}$, and $Cu^{II}Cu^{II}$. *J. Am. Chem. Soc.* **2005**, *127*, 16032–16033. https://doi.org/10.1021/ja056071l.
4. Li, L.; Brennessel, W.W.; Jones, W.D. C−H Activation of Phenyl Imines and 2-Phenylpyridines with $[Cp^*MCl_2]_2$ (M = Ir, Rh): Regioselectivity, Kinetics, and Mechanism. *Organometallics* **2009**, *28*, 3492–3500. https://doi.org/10.1021/om9000742.
5. Hu, Y.; Li, L.; Shaw, A.P.; Norton, J.R.; Sattler, W.; Rong, Y. Synthesis, Electrochemistry, and Reactivity of New Iridium(III) and Rhodium(III) Hydrides. *Organometallics* **2012**, *31*, 5058–5064. https://doi.org/10.1021/ om300398r.
6. Hu, Y.; Norton, J.R. Kinetics and thermodynamics of $H^-/H^\bullet/H^+$ transfer from a rhodium(III) hydride. *J. Am. Chem. Soc.* **2014**, *136*, 5938–5948. https://doi.org/10.1021/ja412309j.
7. Kang, J.W.; Moseley, K.; Maitlis, P.M. Pentamethylcyclopentadienylrhodium and -iridium halides. I. Synthesis and properties. *J. Am. Chem. Soc.* **1969**, *91*, 5970–5977. https://doi.org/10.1021/ja01050a008.
8. Harris, R.K.; Becker, E.D.; Cabral De Menezes, S.M.; Goodfellow, R.; Granger, P. NMR nomenclature: Nuclear spin properties and conventions for chemical shifts (IUPAC recommendations 2001). *Concepts Magn. Reson.* **2002**, *14*, 326–346. https://doi.org/10.1002/cmr.10035.
9. Clark, R.C.; Reid, J.S. The analytical calculation of absorption in multifaceted crystals. *Acta. Crystallogr. A* **1995**, *51*, 887–897. https://doi.org/10.1107/S0108767395007367.
10. Rigaku Oxford Diffraction. *CrysAlisPro Software Systems, Rigaku Corporation, Oxford, UK* **201-2020**.
11. Sheldrick, G.M. SHELXT - integrated space-group and crystal-structure determination. *Acta. Crystallogr. A* **2015**, *71*, 3–8. https://doi.org/10.1107/S2053273314026370.
12. Gritzner, G.; Kuta, J. Recommendations on reporting electrode potentials in nonaqueous solvents (Recommendations 1983). *Pure Appl. Chem.* **1984**, *56*, 461–466. https://doi.org/10.1351/pac198456040461.
13. Noviandri, I.; Brown, K.N.; Fleming, D.S.; Gulyas, P.T.; Lay, P.A.. The Decamethylferrocenium/ Decamethylferrocene Redox Couple: A Superior Redox Standard to the Ferrocenium/Ferrocene Redox Couple for Studying Solvent Effects on the Thermodynamics of Electron Transfer. *J Phys Chem B* **1999**, *103*, 6713–6722. https://doi.org/10.1021/jp991381+.
14. Burchat, A.F.; Chong, J.; Nielsen, N. Titration of alkyllithiums with a simple reagent to a blue endpoint. *J. Organomet. Chem.* **1997**, *542*, 281–283. https://doi.org/10.1016/S0022-328X(97)00143-5.
15. Krasovskiy, A.; Knochel, P. Convenient Titration Method for Organometallic Zinc, Magnesium, and Lanthanide Reagents. *Synthesis* **2006**, *2006*, 890–891. https://doi.org/10.1055/s-2006-926345.
16. Albinati, A.; Bakhmutov, V.I.; Caulton, K.G.; Clot, E.; Eckert, J.; Eisenstein, O.; Gusev, D.G.; Grushin, V.V.; Hauger, B.E. Reaction of molecular hydrogen (H_2) with chlorohydridoiridium phosphines $IrHCl_2P_2$ (P = P^iPr_3 or P^tBu_2Ph): stereoelectronic control of the stability of molecular H_2 transition metal complexes. *J. Am. Chem. Soc.* **1993**, *115*, 7300–7312. https://doi.org/10.1021/ja00069a032.
17. Desrosiers, P.J.; Cai, L.; Lin, Z.; Richards, R.; Halpern, J. Assessment of the "T1 criterion" for distinguishing between classical and nonclassical transition-metal hydrides: hydride relaxation rates in tris(triarylphosphine)osmium tetrahydrides and related polyhydrides. *J. Am. Chem. Soc.* **1991**, *113*, 4173–4184. https://doi.org/10.1021/ja00011a019.
18. Kubas, G.J. Dihydrogen complexes as prototypes for the coordination chemistry of saturated molecules. *Proc. Natl. Acad. Sci. USA* **2007**, *104*, 6901–6907. https://doi.org/10.1073/pnas.0609707104.
19. Kubas, G.J. Activation of dihydrogen and coordination of molecular H_2 on transition metals. *J. Organomet. Chem.* **2014**, *751*, 33–49. https://doi.org/10.1016/j.jorganchem.2013.07.041.
20. Spek, A.L. PLATON SQUEEZE: a tool for the calculation of the disordered solvent contribution to the calculated structure factors. *Acta Crystallogr. C* **2015**, *71*, 9–18. https://doi.org/10.1107/S2053229614024929.
21. Sheldrick, G.M. A short history of SHELX. *Acta. Crystallogr. A* **2008**, *64*, 112–122. https://doi.org/10.1107/ S0108767307043930.
